# Enhanced electrocatalytic biomass oxidation at low voltage by Ni^2+^-O-Pd interfaces

**DOI:** 10.1038/s41467-024-50325-w

**Published:** 2024-07-13

**Authors:** An Pei, Peng Wang, Shiyi Zhang, Qinghua Zhang, Xiaoyi Jiang, Zhaoxi Chen, Weiwei Zhou, Qizhen Qin, Renfeng Liu, Ruian Du, Zhengjian Li, Yongcai Qiu, Keyou Yan, Lin Gu, Jinyu Ye, Geoffrey I. N. Waterhouse, Wei-Hsiang Huang, Chi-Liang Chen, Yun Zhao, Guangxu Chen

**Affiliations:** 1 School of Environment and Energy, State Key Laboratory of Luminescent Materials and Devices, Guangdong Provincial Key Laboratory of Atmospheric Environment and Pollution Control, South China University of Technology, Guangzhou, China; 2 Institute of Physics, Chinese Academy of Sciences, Beijing, China; 3 School of Materials Science and Engineering, Tsinghua University, Beijing, China; 4 College of Chemistry and Chemical Engineering, Xiamen University, Xiamen, China; 5 School of Chemical Sciences, The University of Auckland, Auckland, New Zealand; 6 National Synchrotron Radiation Research Center (NSRRC), Hsinchu, Taiwan

**Keywords:** Electrocatalysis, Catalyst synthesis, Electrochemistry

## Abstract

Challenges in direct catalytic oxidation of biomass-derived aldehyde and alcohol into acid with high activity and selectivity hinder the widespread biomass application. Herein, we demonstrate that a Pd/Ni(OH)_2_ catalyst with abundant Ni^2+^-O-Pd interfaces allows electrooxidation of 5-hydroxymethylfurfural to 2, 5-furandicarboxylic acid with a selectivity near 100 % and 2, 5-furandicarboxylic acid yield of 97.3% at 0.6 volts (versus a reversible hydrogen electrode) in 1 M KOH electrolyte under ambient conditions. The rate-determining step of the intermediate oxidation of 5-hydroxymethyl-2-furancarboxylic acid is promoted by the increased OH species and low C–H activation energy barrier at Ni^2+^-O-Pd interfaces. Further, the Ni^2+^-O-Pd interfaces prevent the agglomeration of Pd nanoparticles during the reaction, greatly improving the stability of the catalyst. In this work, Pd/Ni(OH)_2_ catalyst can achieve 100% 5-hydroxymethylfurfural conversion and >90% 2, 5-furandicarboxylic acid selectivity in a flow-cell and work stably over 200 h under a fixed cell voltage of 0.85 V.

## Abstract (web summary)

Catalytic oxidation of biomass-derived aldehydes and alcohols into acids is challenging due to low activity and selectivity. Here the authors report a Pd/Ni(OH)_2_ electrocatalyst with abundant Ni^2+^-O-Pd interfaces that enables efficient electrooxidation of 5-hydroxymethylfurfural to 2,5-furandicarboxylic acid with high selectivity and product yield.

## Introduction

2, 5-furandicarboxylic acid (FDCA) is a prized value-added chemical from biomass processing and is finding increasing usage as a feedstock for the manufacture of biodegradable plastics^1,2^. Traditionally, FDCA has been prepared by the aerobic oxidation of biomass-derived 5-hydroxymethylfurfural (HMF), though this approach suffers from poor FDCA selectivity and has environmental impacts^3,4^. The electrocatalytic HMF oxidation reaction (HMFOR) at room temperature with water as the oxygen source has recently emerged as a promising route towards FDCA^5,6^. However, electrooxidation of HMF to FDCA typically requires high oxidation potentials, leading to low FDCA selectivity and poor catalyst stability^7–9^. To date, considerable effort has been directed towards developing improved electrode materials for the selective electrooxidation of HMF to FDCA, with the oxides and hydroxides of certain transition metals (TMs, Ni, Co, etc.) generally being pursued for this reaction^10–13^. HMF oxidation over these oxide and hydroxide-based catalysts generally follows a nucleophile oxidation reaction mechanism, in which the catalysts need to be pre-oxidized to generate high-valence active metal species at high oxidation potentials (>1.4 V versus reversible hydrogen electrode, RHE)^12–16^. For instance, Co^4+^ and Co^3+^ can oxidize the hydroxymethyl and aldehyde groups of HMF to aldehyde and carboxylate groups, respectively. However, Co^2+^ is completely inert towards HMF oxidation^12^. Unfortunately, HMFOR at high oxidation potentials will inevitably result in the oxidation of the carbon substrate of the catalyst/electrode while also promoting other undesirable side reactions^12,13^ such as oxygen evolution reaction (OER) or the dissolution of electrode materials^17,18^. All these processes result in irreversible damage to the catalyst, thereby affecting catalyst activity, selectivity, and long-term durability. Therefore, discovering electrocatalysts that can effectively convert HMF to FDCA at low oxidation potentials (<1 V versus RHE) is paramount.

Pd-based catalysts exhibit activity for the HMFOR at low potentials (approximately 0.3 V versus RHE), but they demonstrate low current densities and poor selectivity towards FDCA^19,20^. A Pd-Au alloy catalyst was reported to deliver a high FDCA selectivity (83%) at 0.9 V versus RHE during the HMFOR, representing good performance at the current stage, but the reaction mechanism was not fully explored^19^. To achieve an even higher FDCA selectivity, optimizing the interaction between active metal nanoparticles and the support is critical^21–26^. Nanoscale interfacial catalysis has drawn increasing attention due to its ability to modify catalytic activity and selectivity^21,27–30^. Previous studies have shown that the rational design of metal-metal (hydro)oxide interfaces^24–26,31,32^ is an effective approach for boosting catalytic activity and tuning the selectivity of multi-step reactions towards a specific product. For example, rationally designed Fe^3+^-OH-Pt interfaces were shown to greatly enhance Pt’s catalytic activity for CO oxidation greatly^24^. Similarly, Pt/Ni(OH)_2_ interfaces were reported to improve the electrooxidation of methanol and enhance CO-tolerance in fuel cells^31^. Researchers have focused on noble metal-TM (hydro)oxides interfaces for HMFOR, including Pt/Ni(OH)_2_^33^, Ir_1_/Co_3_O_4_^34^, Ru_1_/NiO^35^, PdO/CuO^36^, etc. However, the noble metals in these catalysts are not the active centers for oxidation but instead serve as additives to enhance the adsorption of HMF or promote the activity of TM active centers (high valent Co or Ni species) for HMFOR, with these catalysts still requiring high oxidation potentials^33–36^. Recently, the Wang group reported Cu foil as an effective electrocatalyst with low onset potential (about 0.1 V vs. RHE) for HMFOR, achieving 100% selectivity to two-electron product (HMFCA), but the six-electron product of FDCA can not be obtained^37^. Based on the above discussion, we hypothesized that developing Pd-based catalysts with active and functional interfaces should allow HMFOR to generate highly selective FDCA at low oxidation potentials (<1 V versus RHE).

In this work, we report a Pd/Ni(OH)_2_ (with Pd <2 nm in diameter and thin Ni(OH)_2_) catalyst rich in Ni^2+^-O-Pd interface for HMFOR, achieving an FDCA selectivity near 100 % at the potential of 0.6 V versus RHE in an alkaline electrolyte under ambient conditions. Detailed experimental and computational studies showed the electrooxidation of HMF at Ni^2+^-O-Pd interfaces involved a direct oxidation mechanism (HMF and its derived intermediates were oxidized on the Pd sites), with the oxidation of HMFCA intermediate being the rate determining step in FDCA production. The Ni^2+^-O-Pd interfaces enhanced the reaction kinetics of HMFCA oxidation by increasing interfacial OH species and lowering the energy barrier for HMFCA oxidation. In addition, the Ni^2+^-O-Pd interfaces effectively suppressed the competing decarbonylation, thereby improving the yield to FDCA and the Faradaic efficiency (FE) of HMFOR. Finally, the Ni^2+^-O-Pd interfaces (and strong associated metal-support interaction) prevented agglomeration of Pd nanoparticles during HMFOR, thereby giving the catalyst excellent stability. With our Pd/Ni(OH)_2_ catalyst in the flow cell reactor (a two-electrode device with the anodic reaction of HMFOR coupled with the cathodic reaction of HER), 100% HMF conversion and >90% FDCA selectivity were achieved with a stable current density during a 200 h continuous HMFOR operation under a fixed cell voltage of 0.85 V and a fixed electrolyte (5 mM HMF in 1 M KOH) flow rate of 1.0 mL/min. Moreover, the Pd/Ni(OH)_2_ catalyst can also work efficiently and stably for more than 24 h without deactivation under the current density of ~380 mA/cm^2^ in a flow cell reactor (flow cell voltage 1.05 V) by the increased the concentration of HMF (125 mM HMF in 1 M KOH) and the flow rate (2.5 mL/min) of the electrolyte. Results demonstrate the importance of interface engineering in the rational design of electrocatalysts for HMFOR to FDCA, with Ni^2+^-O-Pd interfaces offering great promise for industrial-scale FDCA manufacture.

## Results and discussion

### Density functional theory (DFT) calculations on Pd(111) for HMFOR

**Fig. 1:**
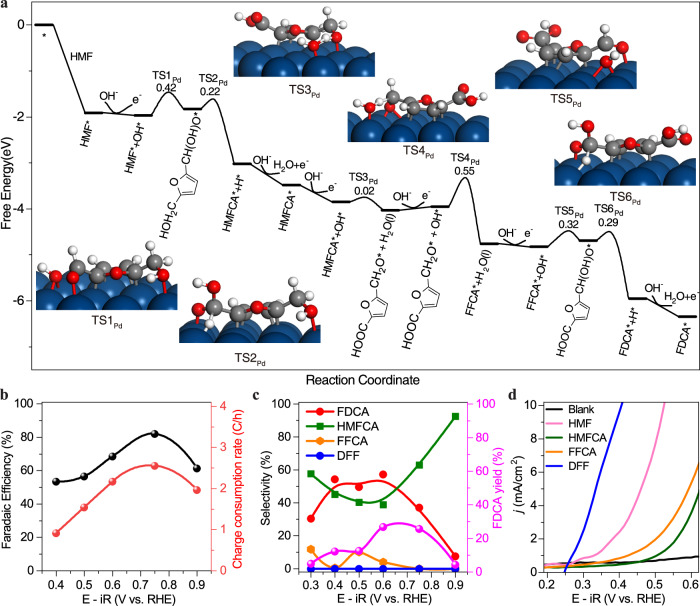
The mechanism and catalytic performance of pristine Pd catalyst for HMFOR. **a** Energies of intermediates and transition states in HMFOR on the Pd(111) surface from DFT calculations. **b** Charge consumption rate on commercial Pd/C catalyst for HMFOR (5 h measurement) at different oxidation potentials and the corresponding FE (the sum of FE from detected liquid products), the corresponding FE lower than 100% were due to the HMF decarbonylation reaction and the additional charge consumption caused by electric double layer capacitance. **c** FDCA yield and the product selectivity of HMFOR on commercial Pd/C catalyst at different oxidation potentials with 95% iR corrected. **d** The LSV measurements for the oxidation of 50 mM substrate (HMF, HMFCA, FFCA, and DFF) in 1 M KOH solution with 95% iR corrected.

We began by modeling the HMFOR on pristine Pd(111) facet in an alkaline electrolyte using DFT calculations to understand why Pd metal catalysts offered such an early on-set potential for HMF oxidation yet also suffered from a low current density and poor selectivity for FDCA. We explored the possible HMFOR pathways and the corresponding reaction energy barriers. Since OH^−^ plays a beneficial role in promoting C–O or C–H bond activation, the electrooxidation of alcohol is usually carried out in alkaline electrolytes, with the reaction rate being closely related to the electrolyte pH^38^. Electrophilic OH* is generated over the Pd (111) with a Gibbs free energy change (ΔG) of −0.17 eV (Supplementary Fig. S1), meaning the HMFOR may proceed through a direct oxidation mechanism rather than the nucleophile oxidation reaction mechanism. The corresponding Gibbs free energy landscapes for HFMOR over Pd(111) were calculated (Fig. 1a and Supplementary Tables S1–S3), with optimized reaction intermediate geometries shown in Fig. 1a and Supplementary Figs. S2–S4. Typically, the reaction started with the electrooxidation of the aldehyde group (CHO), leading to five possible reaction paths (Supplementary Fig. S2): CHO group was first coupled with OH* and converted to gem-diolate anion intermediate (R-CHO-OH*, where R was furylmethanol group), which was then followed by the reaction of C–H bond scission without (I) or with (II) OH* to generate HMFCA*; HMFCA* can also be produced by the coupling of OH* with R-CO* which was generated from the direct dehydrogenation of R-CHO* (III) to R-CO* or OH*-assisted C–H scission of R-CHO* (IV) to R-CO*. The fifth reaction path was the C–C bond breaking (decarbonylation) of R-CO* with CO* formation (V) (Supplementary Fig. S5 and Table S1). Reaction path I was the most favorable because the CHO group had the lowest energy barrier for coupling with OH* (TS1_Pd_, 0.42 eV). Then, HMFCA* was oxidized further to formylfurancarboxylic acid (FFCA) by surface-bound OH* (Fig. 1a and Supplementary Fig. S3), with O–H bond activation via proton transfer having a lower activation barrier than C-H bond scission (TS3_Pd_ vs TS3′_Pd_, 0.02 vs 0.77 eV) (Supplementary Fig. S3, Tables S1 and S2). The generated OH* consecutively attacked the H atoms of R′-CH_2_O* (R′ was 2-Furoic acid group) or R′-CH(OH) to produce FFCA with activation barriers of 0.55 eV (TS4_Pd_) and 0.04 eV (TS4′′_Pd_), respectively (Supplementary Fig. S3, Tables S1 and S2). These indicate that the HMFCA electrooxidation step may occur prior to the elimination of the O-H bond of the hydroxymethyl group under the assistance of OH*. The calculated reaction mechanism for aldehyde group electrooxidation of FFCA follows a similar reaction pathway to HMF oxidation. Based on the above DFT calculations, the C–H bond activation step in HMFCA oxidation was suggested to be the rate-determining step with the highest energy barrier of 0.55 eV (TS4_Pd_) (Fig. 1a, Supplementary Fig. S3 and Tables S1–S3). In addition, we noticed that the electrooxidation of HMF and FFCA on Pd (111) suffered from decarbonylation (Supplementary Figs. S5 and S6). For example, C–C bond cleavage of R-CO* occurred naturally on Pd to yield R* and CO* with modest barriers (${{{{{{\rm{TS}}}}}}1{\prime} }_{{{{{{\rm{Pd}}}}}}}^{{{{{{\rm{CO}}}}}}}$ and ${{{{{{\rm{TS}}}}}}2{\prime} {\prime} }_{{{{{{\rm{Pd}}}}}}}^{{{{{{\rm{CO}}}}}}}$, 0.66 and 0.46 eV (Supplementary Fig. S5 and Table S4), suggesting that CO can be produced during HMFOR. CO formation during HMFOR was experimentally confirmed on Pd/C (Supplementary Figs. S7, S8). CO can poison Pd catalysts, leading to the low activity of HMFOR (Supplementary Fig. S9). CO stripping experiment revealed that CO can be removed from the Pd surface at potentials above 0.67 V versus RHE (Supplementary Fig. S10).

### Electrocatalysis performance of pristine Pd catalyst for HMFOR

To explore the impact of CO poisoning on the kinetics of the HMFOR, we carried out HMFOR with commercial Pd/C catalyst under different oxidation potentials (Fig. 1b,c, and Supplementary Figs. S11–S12). Both the FE and charge consumption rate showed a volcano curve-like potential dependence. The highest charge consumption rate of 2.5 C/h and FE of 82% for HMFOR was achieved at 0.75 V versus RHE. The activity of Pd decreased at potentials higher than 0.90 V versus RHE due to the formation of PdO_x_ (Fig. 1b,c)^39^. A similar trend was observed in the selectivity to FDCA with increasing oxidation potentials. The highest FDCA selectivity (about 58%) was achieved at 0.40 ~ 0.60 V versus RHE. There was a clear trade-off relationship between the selectivity of FDCA and HMFCA (Fig. 1c). In particular, at potentials above 0.75 V versus RHE, the selectivity to HMFCA increased sharply, while that to FDCA decreased sharply. Above 0.75 V versus RHE, CO can be efficiently removed from the Pd surface, demonstrating that HMFCA formation rather than CO poisoning was the main factor responsible for the low selectivity of FDCA at high oxidation potentials. In addition, because of the decarbonylation and self-condensation of HMF (to form humins), the FDCA yield (25.8% at 0.75 V versus RHE) was very low (Fig. 1c). We, therefore, proposed a detailed HMF oxidation process on Pd with possible reaction paths (Supplementary Fig. S13).

To establish that HMFCA oxidation was the rate-determining step (RDS) in HMFOR, we investigated the onset oxidation potentials (at the current density of 1 mA/cm^2^) of HMF and all intermediates including HMFCA, FFCA, and 2, 5-diformylfuran (DFF) on the Pd/C catalyst using linear sweep voltammetry (LSV) (Fig. 1d and Supplementary Fig. S14). HMFCA oxidation showed an onset oxidation potential at 0.50 V versus RHE, much higher than those of HMF (0.33 V versus RHE), FFCA (0.42 V versus RHE) and DFF (0.27 V versus RHE), respectively. Combined with our DFT calculations, we conclude that the key to improving the activity of Pd catalysts for HMFOR and the selectivity of FDCA is to reduce the energy barrier for C-H bond activation during HMFCA oxidation and block decarbonylation on Pd catalysts.

### Preparation and characterization of Ni^2+^-O-Pd interfaces

**Fig. 2:**
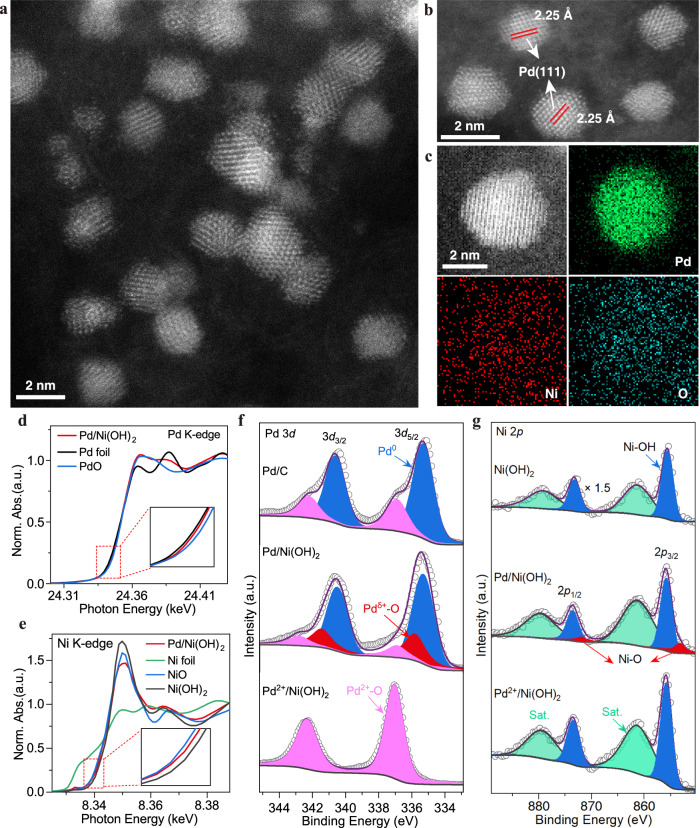
Structural characterizations of the Pd/Ni(OH)_2_ catalyst. **a**, **b** The representative probe-corrected STEM image of Pd/Ni(OH)_2_ catalyst. **c** Probe-corrected STEM image and corresponding EDS mapping of Pd/Ni(OH)_2_ catalyst. **d**, **e** Pd K-edge and Ni K-edge XANES spectra for the Pd/Ni(OH)_2_ catalyst, respectively. **f** Pd 3*d* and (**g**) Ni 2*p* XPS spectra for the Pd/Ni(OH)_2_ catalyst, respectively.

To address these challenges, we proposed a catalytically active site, Ni^2+^-O-Pd interfaces. We serendipitously discovered that the simple physical mixing Ni(OH)_2_ nanosheets (Supplementary Fig. S15) with a Pd/C catalyst can significantly promote the reaction kinetics of HMFOR (Supplementary Fig. S16). To take full advantage of this discovery, we then prepared a Pd/Ni(OH)_2_ catalyst with well-defined and abundant Ni^2+^-O-Pd interfaces. To maximize the utilization of Pd, we prepared the Pd/Ni(OH)_2_ catalyst using a selective pre-deposition and in-situ electrochemical reduction strategy (See Methods in supplementary information). In this strategy, Ni(OH)_2_ nanosheets were pre-deposited on carbon black (Supplementary Fig. S17), after which an acid-base neutralization reaction with sodium tetrachloropalladate solution (pH ~ 4.0) was used to obtain Pd^2+^ supported on thin Ni(OH)_2_ (Pd^2+^/Ni(OH)_2_). This precursor was then electrochemically reduced to give a Pd/Ni(OH)_2_ catalyst. Powder X-ray diffraction (XRD) (Supplementary Fig. S18) and wavelet transformation analysis of the Ni K-edge spectra (Supplementary Fig. S19) revealed that the thin Ni(OH)_2_ nanosheets were obtained after being etched in the sodium tetrachloropalladate solution, with the obtained Pd^2+^/Ni(OH)_2_ precursor then being successfully electrochemically reduced into a Pd/Ni(OH)_2_ catalyst (Supplementary Fig. S18). Probe-corrected scanning transmission electron microscopy (STEM) images (Fig. 2a,b, and Supplementary Fig. S20) of Pd/Ni(OH)_2_ revealed that small Pd nanoparticles (NPs) were well-dispersed on thin Ni(OH)_2_. Even though the size of Pd NPs was only 2 nm, well-defined lattice fringes with interplanar distances of 0.225 nm, corresponding to Pd(111) planes, were clearly observable. Energy dispersive spectroscopy (EDS) analysis showed that Pd NPs were in intimate contact with the thin Ni(OH)_2_, forming abundant Ni^2+^-O-Pd interfaces (Fig. 2c and Supplementary Fig. S21). Pd 3*d* X-ray photoelectron spectroscopy (XPS) spectra suggested that Pd^0^ was the dominant palladium species in the Pd/Ni(OH)_2_ catalyst (62% of the total Pd), with around 26% of the total palladium in the form of a Pd^δ+^ species and 12% was Pd^2+^ species (Fig. 2f). We associate this Pd^δ+^ species with Ni^2+^-O-Pd interfaces. Pd K-edge X-ray absorption near-edge structure (XANES) and the wavelet transformation analysis of the Pd K-edge spectra verified that the Pd NPs were slightly oxidized (Fig. 2d and Supplementary Fig. S22), consistent with the Pd 3*d* XPS data. Extended X-ray absorption fine structure (EXAFS) revealed that the Pd-O and Pd-Pd bond distances for Pd/Ni(OH)_2_ catalyst were 2.01 ± 0.02 Å and 2.72 ± 0.03 Å (Supplementary Fig. S23 and Table S5), respectively, with Pd–O and Pd–Pd coordination numbers of 1.2 ± 0.2 and 3.6 ± 0.7 (Supplementary Table S5). The Ni 2*p* XPS spectrum of Pd/Ni(OH)_2_ showed two distinct Ni^2+^ species, 83% as Ni^2+^ in Ni(OH_2_) and 17% as Ni^2+^ in Ni^2+^-O-Pd interfaces (Fig. 2g). Further, the Ni K-edge for Ni in Pd/Ni(OH)_2_ was shifted to lower energy compared to the Ni(OH)_2_ reference (Fig. 2e), corresponding to a reduction in Ni oxidation state owing to the creation of Ni^2+^-O-Pd interfaces, revealing the strong electronic interaction between Ni(OH)_2_ and Pd at Ni^2+^-O-Pd interfaces^40^. Ni K-edge EXAFS showed Ni-O and Ni-OH bond distances in the Pd/Ni(OH)_2_ catalyst were 1.83 ± 0.02 Å and 2.04 ± 0.02 Å (Supplementary Fig. S23 and Table S6), respectively, and the Ni-O and Ni-OH coordination numbers 2.1 ± 0.4 and 3.9 ± 0.8, respectively (Supplementary Table S6). Evidence for a strong electronic interaction between Pd and the thin Ni(OH)_2_ was also seen in electrochemical CO stripping experiments (Supplementary Fig. S24). The electrochemically active surface area (ECSA) of Pd on the Pd/Ni(OH)_2_ catalyst measured by CO stripping was 30.34 m^2^/g_Pd_, which increased to 56.15 m^2^/g_Pd_ after the Ni(OH)_2_ was acid-leached (Supplementary Fig. S24 and Table S7). The data confirms a Pd/Ni(OH)_2_ catalyst with abundant Ni^2+^-O-Pd interfaces was successfully prepared.

### Electrocatalysis performance of Ni^2+^-O-Pd interfaces for HMFOR

**Fig. 3:**
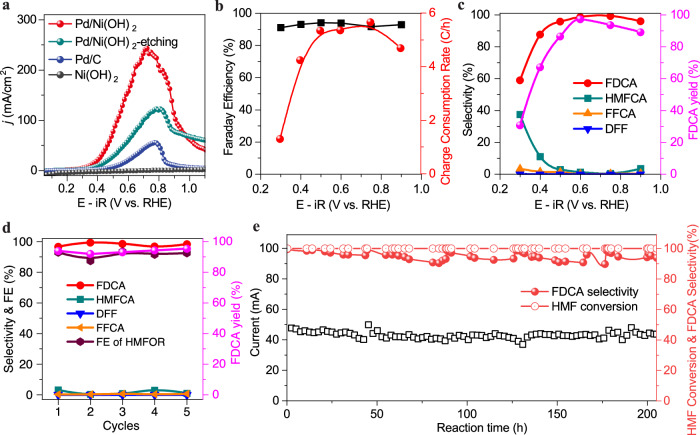
Electrochemical HMFOR performance on Pd/Ni(OH)_2_ catalyst. **a** Polarization curves collected on different catalysts for HMFOR, with 95% iR corrected. **b** Charge consumption rate on the Pd/Ni(OH)_2_ catalyst for HMFOR (5 h measurement) at different oxidation potentials and the corresponding FE (the sum of FE from detected liquid products) with 95% iR corrected. **c** The FDCA yield and the product selectivity of HMFOR on Pd/Ni(OH)_2_ catalyst at different oxidation potentials. The HMFOR was carried out under an argon atmosphere (without the oxygen reduction reaction), and the other part of FE was ascribed to the decarbonylation. **d** Selectivity, FEs, and FDCA yield at 0.75 V for continuous 5 cycles of HMFOR on Pd/Ni(OH)_2_. **e** Stability test of Pd/Ni(OH)_2_ catalyst under a fixed cell voltage of 0.85 V. The cathode and anode were separated by a Nafion membrane. To prevent HMF self-condensation in the alkaline solution, the 2 M KOH solution and 10 mM HMF solution were mixed evenly before being pumped into the anode side of the flow cell reactor (The catalyst was activated by CV cycle in the potential between −1 and −2.5 V every 40 h). There were no apparent changes to the cell current density or conversion of HMF or FE of FDCA during this 200-h continuous HMFOR operation. Reaction conditions: 1.0 M KOH + 5 mM HMF with a flow rate of 1.0 mL/min.

We then focused on studying the performance of the Pd/Ni(OH)_2_ catalyst for HMFOR, investigating the selectivity and FE toward different products. Carbon-supported Ni(OH)_2_ nanosheets displayed negligible activity for HMFOR (Fig. 3a). The Pd/Ni(OH)_2_ catalyst displayed high activity and stability relative to commercial Pd/C (Figs. 3a, c, and 1b–d). A very low HMFOR onset potential of 0.22 V versus RHE was achieved on the Pd/Ni(OH)_2_ catalyst, which is 0.11 V versus RHE earlier than that of Pd/C (0.33 V versus RHE). A peak current density of 236.1 mA/cm^2^ and specific current density (normalized to ECSA) of 1.71 mA/cm^2^ were achieved at 0.75 V versus RHE, about 4.4 and 6.3 times higher than Pd/C (53.3 mA/cm^2^ and 0.27 mA/cm^2^, respectively) (Fig. 3a and Supplementary Fig. S25). The charge consumption rate for HMFOR on Pd/Ni(OH)_2_ reached as high as 5.6 C/h at 0.75 V versus RHE (Fig. 3b), which was greater than that of Pd/C (2.5 C/h) by a factor of 2.2. The FEs of HMFOR on Pd/Ni(OH)_2_ were all above 90% in the potential range of 0.3 ~ 0.9 V versus RHE (Fig. 3b and Supplementary Fig. 26). More importantly, an FDCA selectivity of almost 100% was observed at 0.6 ~ 0.75 V versus RHE (Fig. 3c), which was 39% higher than that of Pd/C. An FDCA yield of 97.3% was achieved at 0.6 V versus RHE (Figs. 3c), 70.5% higher than that of Pd/C, due to the fast reaction rate of HMFOR overcoming HMF self-condensation (Supplementary Fig. S27). Furthermore, the FE of HMFOR, FDCA selectivity, FDCA yield, and Pd/Ni(OH)_2_ catalyst stability were maintained even after 5 reaction cycles with the same catalyst (Fig. 3d and Supplementary Figs. S28–30). The Pd/Ni(OH)_2_ catalysts for the electrooxidation of HMF to FDCA showed good performance at low voltage (Supplementary Table S8). In addition, the potential for practical HMFOR application was further demonstrated by equipping the Pd/Ni(OH)_2_ catalyst into a two-electrode flow cell reactor (Fig. 3e and Supplementary Figs. S31–33), in which 100% HMF conversion and >90% FDCA selectivity were achieved during a 200 h continuous HMFOR operation under a fixed cell voltage of 0.85 V with 95% iR corrected and a fixed electrolyte (5 mM HMF in 1 M KOH) flow rate of 1 mL/min (Fig. 3e). Moreover, the Pd/Ni(OH)_2_ catalyst can also work efficiently and stably for more than 24 h without degradation under the current density of ~380 mA/cm^2^ in the similar flow cell reactor with similar operation parameters except for the increased flow cell voltage (to 1.05 V), concentration of HMF (125 mM HMF in 1 M KOH), and the flow rate (2.5 mL/min) of the electrolyte (Supplementary Fig. S34), which suggests that the Pd/Ni(OH)_2_ catalyst was robust under the practical catalytic conditions.

To investigate the active site of the Pd/Ni(OH)_2_ catalyst for HMFOR, Pd^2+^/Ni(OH)_2_ (Fig. 2f–g, and Supplementary Fig. S35) without electrochemical reduction was tested under the same reaction conditions. Notably, Pd^2+^/Ni(OH)_2_ had negligible activity towards HMFOR, indicating that Pd^2+^ was inert to HMF activation (Supplementary Fig. S36). In addition, the enhanced activity and stability of Pd/Ni(OH)_2_ were lost when the catalyst was treated with acid to remove Ni(OH)_2_ (denoted as Pd/Ni(OH)_2_-etching) (Fig. 3a, and Supplementary Figs. S25 and S37–41), with the performance of Pd/Ni(OH)_2_-etching catalyst being similar to Pd/C (Fig. 1b, c and Supplementary Figs. S10, 11). Electrochemical impedance spectroscopy (EIS) measurements demonstrated that the Pd/Ni(OH)_2_ catalyst showed a much smaller charge transfer resistance (R_ct_) for HMFOR than Pd/Ni(OH)_2_-etching and Pd/C (Supplementary Fig. S42), firmly establishing that Ni^2+^-O-Pd interfaces were the active sites for HMFOR.

### Study on HMFOR mechanism of Ni^2+^-O-Pd interfaces

**Fig. 4:**
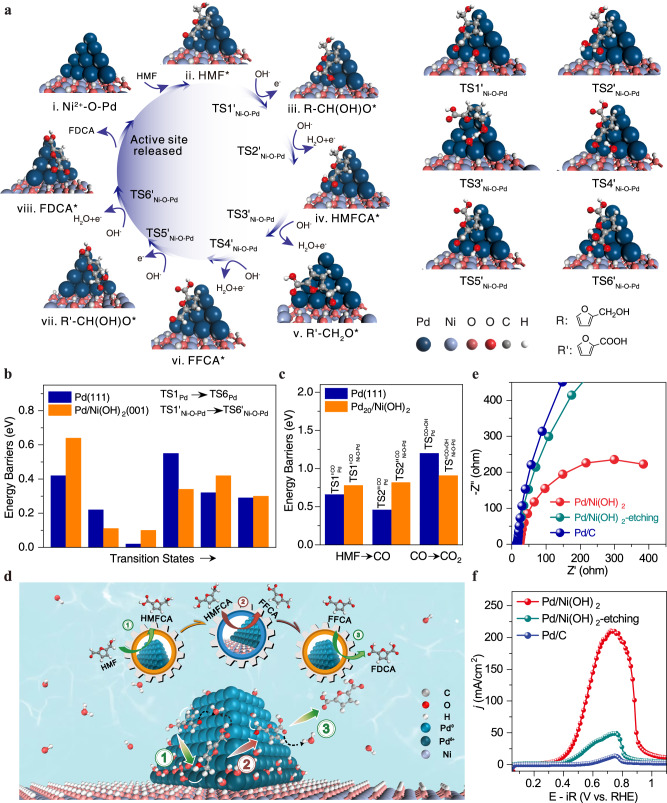
Mechanism of HMFOR on Ni^2+^-O-Pd interfaces. **a** The catalytic cycle for HMFOR to FDCA on Ni^2+^-O-Pd interfaces derived from DFT calculations, including the intermediates and transition states. **b** Comparison of the energy barriers for different transition states on the Pd(111) surface and Ni^2+^-O-Pd interface. **c** Comparison of the energy barriers of the transition states for decarbonylation and CO oxidation on Pd(111) surface and Ni^2+^-O-Pd interface. **d** Proposed synergistic reaction process of HMFOR at Ni^2+^-O-Pd interfaces. Pd(111) favors aldehyde group electrooxidation, while the Ni^2+^-O-Pd interface favors HMFCA electrooxidation. **e** Electrochemical impedance spectroscopy measurements for HMFCA oxidation. **f** Kinetic studies of HMFCA oxidation over different catalysts. Polarization curves were measured in 1 M KOH + 50 mM HMFCA solution with a scanning rate of 5 mV/s with 95% iR corrected.

To better understand how Ni^2+^-O-Pd interfaces promoted the performance of HMFOR, a structural model consisting of periodic Ni(OH)_2_ (001) supporting a Pd_20_ cluster was built (Supplementary Fig. S43) to simulate the Ni^2+^-O-Pd interface in the Pd/Ni(OH)_2_ catalyst. The Bader charge analysis showed that the Pd atoms at the Ni^2+^-O-Pd interface carried considerable positive charges (Pd^δ+^, +0.14 ~ 0.35 |e|) (Supplementary Table S9), indicating significant electron transfer from interfacial Pd atoms to Ni(OH)_2_, consistent with the XPS and XANES findings (Fig. 2d–g). Compared with pristine Pd, the adsorption energy of HMF on Pd/Ni(OH)_2_ was greatly reduced to −1.28 eV (c.f. −1.91 eV for Pd) (Supplementary Fig. S44 and Table S1) owing to the positive charged Pd^δ+^ at the interface. DFT calculations showed that HMFOR at Ni^2+^-O-Pd interfaces followed an appropriate reaction path similar to that of the pristine Pd catalyst, namely HMF*→R-CHO-OH*→HMFCA*→R’-CH_2_O*→FFCA*→R’-CHO-OH*→FDCA*. The catalytic cycle and Gibbs free energy diagram of HMFOR over the Pd/Ni(OH)_2_ catalyst, derived from the DFT calculations, are displayed in Fig. 4a, Supplementary Fig. S45, and Table S1. Comparing Pd(111) and the Ni^2+^-O-Pd interface, we find that the former favors aldehyde group electrooxidation while the latter favors HMFCA electrooxidation. As shown in Fig. 4b, the RDS barriers for aldehyde group electrooxidation in HMF and FFCA on Pd(111) increased from 0.42 (TS1_Pd_) to 0.64 eV (TS1’_Ni-O-Pd_) and from 0.32 (TS5_Pd_) to 0.42 eV (TS5’_Ni-O-Pd_), respectively, when compared with the Ni^2+^-O-Pd interface. In contrast, the RDS barrier (C-H bond activation) for the HMFCA conversion to FFCA over the Ni^2+^-O-Pd interface (0.34 eV) was much lower than on the Pd(111) surface (0.55 eV). These results show that the oxidation of the aldehyde group of HMF may preferentially occur on the Pd^0^ atoms of the Pd/Ni(OH)_2_ catalyst. In contrast, the oxidation of the hydroxylmethyl group of HMFCA will preferentially occur at Ni^2+^-O-Pd interfaces. Our results suggest that the prepared Pd/Ni(OH)_2_ catalyst has combined the advantages of Pd^0^ sites and Ni^2+^-O-Pd interfaces, both of which may work synergetically with a tandem mechanism to selectively produce FDCA from HMFOR, as schematically depicted in Fig. 4d. Kinetic studies together with EIS measurements (Fig. 4e), potential step curves (Supplementary Fig. S46) and polarization curves (Fig. 4f and Supplementary Fig. S47) for HMFCA oxidation suggested the Ni^2+^-O-Pd interfaces overcame the high energy barrier for C-H bond activation, thereby allowing efficient HMFCA oxidation.

For HMFCA conversion to FFCA, the cleavage of the C-H bond (R’-CH_2_O*) occurred at the Ni^2+^-O-Pd interface with the assistance of a hydroxyl group (OH*, TS4’_Ni-O-Pd_) (Supplementary Fig. S45 and Table S1). Experimental studies confirmed that HMFOR was a pH-dependent and potential-dependent reaction (Supplementary Fig. S48). Obviously, generating OH* active species played an important role in promoting the HMFOR performance. Compared with the Pd(111) facet (−0.17 eV), OH^−^ can be effectively oxidized to OH* active species with a lower energy barrier at Ni^2+^-O-Pd interfaces (−0.37 eV) (Supplementary Fig. S1). Cyclic voltammetry studies showed a much higher OH adsorption/desorption current density on the Pd/Ni(OH)_2_ catalyst relative to the Pd/C at 0.32 and 0.25 V versus RHE (Supplementary Fig. S49), indicating that Ni^2+^-O-Pd interface can accumulate OH at the interfaces. Therefore, the efficient generation of OH* at Ni^2+^-O-Pd interfaces facilitated the conversion of R’-CH_2_O* intermediates to FFCA (Fig. 4a, b, and Supplementary Fig. S45), improving overall HMFOR efficiency.

Another important reason was that Ni^2+^-O-Pd interfaces could inhibit the cleavage of the C–H bond (CHO) of HMF (FFCA) and suppress the formation of R-CO* (R’-CO*) intermediates, whose next step was the C–C bond cleavage to form CO. As shown in Fig. 4c, Supplementary Fig. S50, and Table S10, the energy barriers for CO formation at Ni^2+^-O-Pd interfaces are higher than those on the Pd(111) surface (0.70 (${{{{{{\rm{TS}}}}}}1{\prime} }_{{{{{{\rm{Ni}}}}}}-{{{{{\rm{O}}}}}}-{{{{{\rm{Pd}}}}}}}^{{{{{{\rm{CO}}}}}}}$), 0.82 eV (${{{{{{\rm{TS}}}}}}2{\prime} {\prime} }_{{{{{{\rm{Ni}}}}}}-{{{{{\rm{O}}}}}}-{{{{{\rm{Pd}}}}}}}^{{{{{{\rm{CO}}}}}}}$) vs. 0.66 (${{{{{{\rm{TS}}}}}}1{\prime} }_{{{{{{\rm{Pd}}}}}}}^{{{{{{\rm{CO}}}}}}}$), 0.46 eV (${{{{{{\rm{TS}}}}}}2{\prime} {\prime} }_{{{{{{\rm{Pd}}}}}}}^{{{{{{\rm{CO}}}}}}}$)) (Supplementary Fig. S4b), indicating that the Pd(111) surface favors the cleavage of the C–H bond (CHO) and C–C bond (R-CO* or R’-CO*) at lower potentials than Ni^2+^-O-Pd interfaces. Further, we also considered the CO removal process. The adsorbed CO* can be removed from the Pd surface by coupling with OH* to form CO_2_ via a COOH* intermediate (Supplementary Fig. S51). The Ni^2+^-O-Pd interfaces displayed higher activity than the Pd(111) surface towards CO oxidation with lower oxidation potential (Supplementary Fig. S24) as well as the lower energy barrier (0.91 (${{{{{{\rm{TS}}}}}}{\prime} }_{{{{{{\rm{Ni}}}}}}-{{{{{\rm{O}}}}}}-{{{{{\rm{Pd}}}}}}}^{{{{{{\rm{CO}}}}}}+{{{{{\rm{OH}}}}}}}$) vs. 1.20 eV (${{{{{{\rm{TS}}}}}}}_{{{{{{\rm{Pd}}}}}}}^{{{{{{\rm{CO}}}}}}+{{{{{\rm{OH}}}}}}}$)) (Supplementary Fig. S51 and Table S11). These results suggest that introducing the Ni^2+^-O-Pd interfaces can block undesirable decarbonylation, prevent CO generation and catalyst poisoning, and thus improve FDCA yield and the FE of HMFOR (Supplementary Figs. S7–8).

Based on our systematic understanding of the mechanism of HMFOR on Ni^2+^-O-Pd interfaces, we expected that our Pd/Ni(OH)_2_ catalyst should be able to efficiently catalyze the conversion of all the possible intermediates of HMFOR to FDCA. As shown in Supplementary Figs. S52–S54, Pd/Ni(OH)_2_ catalyst exhibited very high activity for the electrooxidation of HMFCA, FFCA, and DFF, respectively, to FDCA, outperforming the Pd/C and Pd/Ni(OH)_2_-etching reference catalysts in all these selective oxidations. As a demonstration of the versatility of the Pd/Ni(OH)_2_ catalyst, we show that the catalyst can selectively electrooxidize many other alcohols into high-value-added products (Supplementary Fig. S55). Immobilizing palladium metal nanoparticles on oxide/hydroxide support to create multifunctional interfaces is a very effective method for preparing efficient, highly selective, and durable electrocatalysts for biomass valorization.

## Methods

### Materials

The catalyst support carbon black (BLACK PEARLS 2000 LOT-1366221) was purchased from Cabot Corporation. Pd/C was purchased from Adamas, Nickel (II) nitrate hexahydrate (Ni(NO_3_)_2_·6H_2_O, Analytical Reagent, AR), sodium hydroxide (NaOH, 99.9%), potassium hydroxide (KOH, 85%), anhydrous ethanol (C_2_H_5_OH, 99.5%), methanol (CH_3_OH, >99.9%), concentrated nitric acid (65%), ammonium formate (CH_5_NO_2_, 99%) were purchased from Aladdin Reagent Company. Sodium tetrachloropalladate (II) (Na_2_PdCl_4_, 99.9%) was supplied by Sino-Platinum Co. Ltd. 5-Hydroxymethyl (HMF, 99%), 5-Formylfuran-2-carboxylic acid (HMFCA, 98%) 2,5-Furandicarboxaldehyde (DFF, 98%), 5-Formylfuran-2-carboxylic acid (FFCA, 98%),2,5-Furandicarboxylic acid (FDCA, 99%) were obtained from Aladdin. The pure water used in all experiments was obtained using a pure water system (Milli-Q, 18.2 MΩ). The above-mentioned reagents were used as received without further purification. Electrolyte KOH and HMF were dissolved in pure water, respectively. To avoid HMF self-polymerization, the electrolyte was used immediately after preparation.

### Preparation of Ni(OH)_2_/C

Firstly, Ni(NO_3_)_2_·6H_2_O (0.877 g) was sonically dissolved in pure water (82.5 mL) for 15 min, followed by vigorous magnetic stirring for 10 min. Then, anhydrous ethanol (12.5 mL) was added dropwise into the solution, followed by vigorous magnetic stirring for another 10 min. Next, carbon black (0.125 g) was added, and the resulting dispersion was sonicated for 15 min, followed by stirring for 1 h to adsorb the nickel ions. Subsequently, 3.6 M NaOH (12.5 mL) was quickly injected into the dispersion under vigorous magnetic stirring to nucleate Ni(OH)_2_ rapidly. The beaker was then covered with plastic wrap, and the dispersion stirred for another 20 h. Finally, the solid product was collected by filtration and then dried under vacuum at 60 °C. The obtained sample is denoted herein as Ni(OH)_2_/C.

### Preparation of Pd/Ni(OH)_2_

Ni(OH)_2_/C (0.084 g) was dispersed in pure water (200 mL) under vigorous magnetic stirring for 30 min. Then, a specific volume of Na_2_PdCl_4_ solution (19 mg/mL) was added to the dispersion under continuous stirring, with the resulting dispersion then stirred for 20 h. Next, the mixture was collected by filtration and dried under a vacuum (60 °C). The obtained sample was denoted herein as Pd^2+^/Ni(OH)_2_. By this approach, Pd^2+^/Ni(OH)_2_ with different Pd loadings was obtained by controlling the volume of the Na_2_PdCl_4_ solution. The Pd^2+^/Ni(OH)_2_ was electrochemically reduced by cyclic voltammetry between 0 V and 0.8 V (vs. RHE) at a scan rate of 200 mV s^−1^ for 100 cycles. This transformed the adsorbed Pd^2+^ to Pd^0^. The obtained catalysts are denoted herein as Pd/Ni(OH)_2_.

### Preparation of Pd/Ni(OH)_2_-etching

The as-prepared Pd/Ni(OH)_2_ (0.035 g) was dispersed in a 0.5 M nitric acid solution (25 mL) under continuous stirring for 1.5 h. After filtration and drying in a vacuum (60 °C) overnight, a Pd/Ni(OH)_2_-etching catalyst was successfully synthesized (in which most of the Ni(OH)_2_ support had been removed).

### Inductively coupled plasma optical emission spectrometry (ICP-OES)

The standardization curves of different Pd and Ni concentrations were collected before sample measurements. Moreover, the sample pretreatment was conducted by microwave digestion in aqua regia with hydrofluoric acid. Then, Pd and Ni loadings in the synthesized catalysts were determined by inductively coupled plasma optical emission spectrometry (ICP-OES) on an iCAP 7200 Duo (Thermofisher Scientific).

### X-ray diffraction (XRD)

The catalysts’ X-ray diffraction (XRD) patterns were collected on an X-ray diffractometer (PANayltical Empyreanquipped with a Cu *K*_*α*_ source (60 mA and 60 kV, 5 ~ 90°).

### X-ray photoelectron spectroscopy (XPS)

X-ray photoelectron spectroscopy (XPS) data were collected on a Thermo Fisher Scientific Escalab Xi+ XPS spectrometer (Al *K*_*a*_ radiation-1486.6 eV).

### Transmission electron microscopy (TEM)

TEM images were obtained on a Talos F200X and JEM 2100 instrument operating at an acceleration voltage of 200 kV.

### Aberration-corrected scanning transmission electron microscopy (AC-STEM)

AC-STEM images and energy-dispersive X-ray measurements (STEM-EDS elemental maps) were collected on an ARM-200CF (JEOL, Tokyo, Japan) operating at a voltage of 200 kV.

### X-ray absorption spectra (XAS)

X-ray absorption spectra (XAS), including X-ray absorption near-edge structure (XANES) and extended X-ray absorption fine structure (EXAFS) data of the catalysts, were collected at the XAS station (BL14W1) of the Shanghai Synchrotron Radiation Facility (SSRF). The Ni K-edge (~8333 eV) and Pd K-edge (~24350 eV) XAS spectra were collected in transmission mode, using a metallic Ni foil and a Pd foil for energy scale calibration reference. The electron storage ring was operated at 3.5 GeV, and a Si (111) crystal was used as the monochromator.

### Electrochemical experiments in three-electrode H-type Cell

An electrochemical workstation (BioLogic EC-Lab) with a traditional three-electrode H-cell was used for electrochemical measurements. HMFOR performance test was conducted in a three-electrode H-type Cell, with a Pt-foil as the counter electrode and Hg/HgO as the reference electrode, and the voltages were 95% iR-corrected. Catalyst inks were prepared by dispersing a catalyst (Pd/Ni(OH)_2_-5.0 mg, Pd/Ni(OH)_2_-etching-5.0 mg, Pd/C-11.11 mg) in a mixture of isopropanol (0.5 mL) and pure water (0.45 mL) and Nafion (0.05 mL) followed by sonication for 0.5 h. Subsequently, 300 *µ*L of catalyst ink was dropped onto the carbon paper (1 × 0.5 cm^2^, 3.0 mg cm^2^). After drying at room temperature, the catalyst-covered carbon paper was utilized as the working electrode in HMFOR measurements. The pH of the electrolytes of 1 M KOH + 50 mM HMF solution was 13.8 ± 0.2 (determined by pH meter). Chronoamperometry tests were performed at different working potentials in an Ar-saturated 1 M KOH + 5 mM HMF solution (10 mL). The cathode and anode were separated by a proton exchange membrane (PEM, Nafion 117). PEM was suitable for a wide pH range (0 ~ 14), and we found through controlled experiments that it was more effective than anion exchange membranes (AEM) in preventing HMF and products from crossing the membrane to the cathode. Moreover, the theoretical maximum transfer coulomb amount was 28.95 C and would not cause a change in pH value. The curves of charge consumption (Q) versus reaction time (t) at each working potential were collected until the reaction finished. An inert gas (Ar) was purged through the electrolyte during the chronoamperometry test to avoid the oxygen reduction reaction (ORR) side reaction. The volume of the reaction solution was measured and collected after the performance test.

### Two-electrode flow cell

An electrochemical workstation (BioLogic EC-Lab) was used for two-electrode flow cell measurements. For the HMFOR performance test in the two-electrode flow cell reaction, the cathode and anode were separated by a proton exchange membrane (Nafion 117), and the voltages were 95% iR-corrected. Commercial 20 wt% Pt/C (10.0 mg) was sprayed on carbon paper (2.0 × 2.0 cm^2^, 2.5 mg/cm^2^) and used as the cathode electrode. An inert gas of Ar was purged through the cathodic electrolyte to avoid unwanted competition from the ORR. The electrolyte of 1.0 M KOH was circulated through the cathode chamber by a peristaltic pump. The Pd/Ni(OH)_2_ catalyst loaded on a carbon felt was utilized as the anode in the two-electrode flow cell. Catalyst inks were prepared by dispersing Pd/Ni(OH)_2_ catalyst (100.0 mg) in a mixture of isopropanol (5.0 mL), pure water (5.0 mL), and Nafion (0.3 mL). Subsequently, carbon felt was immersed in catalyst ink and dried at room temperature. The anodic electrolyte of 5 mM HMF in 1.0 M KOH was prepared by mixing 10 mM HMF aqueous solution with 2.0 M KOH before pumping into the anode chamber with a flow rate of 1.0 mL/min. The flow cell voltage was fixed at 0.85 V to drive the cathodic HER and anodic HMFOR. The electrolyte pumping out from the anode was collected, and the product was analyzed using HPLC.

### Cyclic voltammetry (CV)

Before the measurements, the reference electrode (Hg/HgO) was calibrated with the standard saturated calomel electrode (SCE) at open-circuit voltage. The wire of the working electrode was connected to the Hg/HgO electrode, and then the wire of the reference and counter electrode were connected to the SCE, the open-circuit voltage was tested and was closed (<10 mV) to the differentials of the standard electrode potential of the Hg/HgO and SCE electrodes. Catalyst inks were prepared by dispersing catalyst (Pd/Ni(OH)_2_-5.0 mg, Pd/Ni(OH)_2_-etching-4.6 mg, Pd/C-9.6 mg, the catalyst mass was calculated according to the Pd loading determined by ICP-OES, to maintain the same quality of Pd on the surface of the working electrode) in a mixture of isopropanol (0.5 mL) and pure water (0.45 mL) and Nafion (0.05 mL) followed by sonication for 0.5 h. Subsequently, 300 *µ*L of catalyst ink was dropped onto the carbon paper (1 × 0.5 cm^2^) as the working electrode. After complete drying at room temperature, the catalyst-covered carbon paper was utilized as the working electrode in HMFOR measurements, and the voltages were 95% iR-corrected. Potentials were corrected to the RHE using the following equation:

1 $${{{{{\rm{E}}}}}}({{{{{\rm{RHE}}}}}})={{{{{\rm{E}}}}}}({{{{{\rm{Hg}}}}}}/{{{{{\rm{HgO}}}}}})+0.0591\times {{{{{\rm{pH}}}}}}+0.098$$

The pH value of the HMFOR electrolyte was measured to 13.8 using a pH meter. Ar gas was purged through the electrolyte to avoid unwanted competition from the ORR. HMFOR polarization curves were obtained in 1 M KOH + 0.05 M HMF by continuous CV at a sweep rate of 5 mV s^−1^ by using a BioLogic EC-Lab workstation.

### In-situ electrochemical impedance spectroscopy (in-situ EIS)

An electrochemical workstation (BioLogic EC-Lab) with a traditional three-electrode H-cell was used for in-situ electrochemical impedance spectroscopy measurements. Work electrodes for in-situ EIS measurements were obtained using the same method as cyclic voltammetry measurements. An Ar gas was purged through the electrolyte to avoid unwanted competition from the ORR. The in-situ electrochemical impedance spectroscopy (in-situ EIS) data for the as-prepared catalysts were collected in the frequency range of 0.1–100,000 Hz in 1 M KOH solution with/without 0.05 M HMF or HMFCA.

### CO-stripping

The electrochemical surface areas (ECSAs) of the different catalysts were estimated using CO-stripping experiments. The CO-stripping measurements involved forming a monolayer of adsorbed CO on the electrocatalysts by holding the electrode potential at 0.1 V (vs. RHE) while bubbling 100% CO through the 1 M KOH electrolyte for 30 min. After that, the working electrode was characterized by cyclic voltammetry for two continuous cycles from 0 V to 1.20 V (vs. RHE) in a pure 1 M KOH solution without added CO, and the voltages were 95% iR-corrected by employing BioLogic EC-Lab workstation.

### In-situ FTIR spectra

Electrochemical in-situ Fourier transform infrared (FTIR) reflection spectroscopy measurements used a liquid-nitrogen-cooled MCT-A detector (Nicolet-8700 spectrometer). The IR beam was passed through a thin solution layer between the working electrode and a CaF_2_ window, allowing both adsorbed and dissolved species to be detected. The catalyst (2.0 mg) was dispersed in an ethanol-water mixture (1:1) under sonication for 0.5 h. 10.0 *μ*L of the obtained ink was applied to a carbon paper working electrode, after which 5.0 *μ*L of a 0.25 wt% Nafion solution was applied. A carbon rod was used as the counter electrode and a Hg/HgO as the reference electrode, respectively. Before the electrochemical in-situ FTIR measurements in 1 M KOH + 0.05 M HMF, the working electrode was first electrochemically cleaned until stable in a N_2_-saturated 1 M KOH solution. Multi-stepped FTIR spectroscopy (MS-FTIRS) was used to collect spectra from 0.1 V to 1.1 V (vs. RHE) at 0.1 V intervals by using a 263 A potentiostat/galvanostat (EG&G) workstation.

### Product analysis

Catalyst inks were prepared by dispersing a catalyst (Pd/Ni(OH)_2_-10.0 mg, Pd/Ni(OH)_2_-etching-9.2 mg, Pd/C-19.2 mg (the catalyst mass was calculated according to the Pd loading determined by ICP-OES, to maintain the same quality of Pd on the surface of the working electrode) in a mixture of isopropanol (1.0 mL) and pure water (0.90 mL) and Nafion (0.10 mL) followed by sonication for 0.5 h. Subsequently, 2 mL of catalyst ink was dropped onto the carbon paper (2 × 1.5 cm^2^) as the working electrode. After drying at room temperature, the catalyst-covered carbon paper was utilized as the working electrode in HMFOR measurements. A continuous inert gas (Ar) was purged through the electrolyte to avoid unwanted competition from the oxygen reduction reaction (ORR). Moreover, suppose the oxygen was not removed cleanly. In that case, the oxygen activation oxidation of HMF on the Pd surface will occur, rather than the electrocatalytic oxidation process, which will cause problems in product detection results and Faraday efficiency calculations. The HMFOR measurements under different oxidation potentials were conducted, and the voltages were 95% iR-corrected by employing a BioLogic EC-Lab workstation. Cycle stability of Pd/C and Pd/Ni(OH)_2_-etching for HMFOR at 0.75 V versus RHE. were repeated independently three times, and Pd/Ni(OH)_2_ was performed independently five times at 0.75 V versus RHE. After a certain period of electrocatalysis, the concentrations of organic compounds in the electrolytes were analyzed by HPLC (LC-20AD, Shimadzu) equipped with a photo-diode array (PDA) detector with a detector wavelength of 265 nm. Samples were prepared for HPLC: 50 *µ*L of electrolyte and 950 *µ*L of an aqueous 5 mM ammonium formate solution (ammonium formate/methanol = 7:3) were mixed. A 5 *µ*m C18 column (WondaSil C18-WR, 4.6 × 250 nm) was used to separate different organic compounds, with the analysis requiring 10 min/sample. The mobile phase component A (70%) was a 5 mM ammonium formate aqueous solution, and component B (30%) was methanol with a total flow rate of 0.6 mL/min (Supplementary Fig. S56).

The Faradaic efficiency (FE) of HMFOR was calculated as follows:

2 $${{{{{\rm{FE}}}}}}(\%)=100\%\times \frac{{charge\; for\; product}}{{total\; charge\; passed}}=100\%\times \frac{n\times m\times 96485{{{{{\rm{C}}}}}}\,{{{{{{\rm{mol}}}}}}}^{-1}}{{total\; charge\; passed}}$$

where n is the number of electron transfers from HMF electrooxidation to each product for DFF and HMFCA (n = 2), FFCA (n = 4), FDCA (n = 6); m is the mole of each product; 96485 C mol^−1^ is the Faraday constant.

3 $${{{{{\rm{Selectivity}}}}}}(\%)=100\%\times \frac{{mol}{es\; of}a{certain\; product}}{{moles\; of\; all\; the\; detected\; products}}$$

4 $${{{{{\rm{Y}}}}}}{{{{{\rm{ield}}}}}}(\%)=100\%\times \frac{{mol}{es\; of}a{certain\; product}}{\,{initial\; moles\; of\; HMF}}$$

### DFT calculations

Ab initio density functional theory calculations were carried out using the Vienna ab initio simulation program (VASP)^41^. All calculations were carried out using a plane-wave cutoff of 400 eV. The exchange and correlation energies were described by the Perdew–Burke–Ernzerhof (PBE) form of the generalized gradient approximation^42^, with dispersive interactions modeled by Grimme’s D3 correction^43^. The self-consistent total energy convergence criteria was less than 10^−5 ^eV per atom, and atom positions were optimized until the Hellman–Feynman force on each atom was smaller than 0.03 eV/Å. A vacuum layer of 15 Å between periodically repeated slabs was set to avoid interactions among the surface and its periodic images. Kinetic barriers were computed using climbing-image nudged elastic band (CI-NEB) and dimer methods with the same optimization criteria^44,45^. Transition states were confirmed through frequency analysis to ensure only one imaginary frequency existed.

The Pd/Ni(OH)_2_ interface was constructed using periodic boundary conditions based on optimized Pd and Ni(OH)_2_ bulk structures. A (5 × 5) supercell of Ni(OH)_2_ (001) slab model with one layer was adopted as the support for loading a Pd_20_ cluster with exposed (111) surface facets (Supplementary Fig. S40). The Pd_20_ cluster and Ni(OH)_2_ were fully relaxed during Pd/Ni(OH)_2_ optimization. The Pd(111) surface slab model was built with a (5 × 5) supercell and four layers. The upper two Pd(111) layers were relaxed, and the bottom two were fixed at the bulk geometry in structure optimization. The Brillouin zone was sampled using a 3 × 3 × 1 k-point mesh in the Monkhorst–Pack setups for the Pd/Ni(OH)_2_ and Pd(111) calculations^46^.

The Gibbs free energy of the species involved in HMFOR was defined as

5 $$\Delta G={\Delta E}_{{{{{{\rm{DFT}}}}}}}+{\Delta E}_{{{{{{\rm{ZPE}}}}}}}-T\varDelta S$$

where ∆*E*_DFT_, ∆*E*_ZPE_, and ∆S denote the DFT-calculated electronic energy difference between reactants and products of reactions, the zero-point energy correction, and the vibrational entropy at room temperature (T = 298.15 K), respectively. The steps involving an electron transfer from OH^−^ to e^−^ in HMFOR, the corresponding free energy change was calculated based on the standard hydrogen electrode method proposed by ref. ^47^. The free energy of OH^−^ was derived as *G*(OH^−^) = *G*(H_2_O(*l*)) − *G*(H^+^), where *G*(H^+^) = 1/2 *G*(H_2_(*g*)) − *k*_B_*T*ln 10 × pH. The pH was set to 14, and *k*_B_ is the Boltzmann constant.

### Supplementary information

Supplementary Information

Peer Review File

## Data Availability

Data generated in this study are provided in the Source Data file. Source data are provided with this paper. 10.6084/m9.figshare.25682826
