# Supplementary material for: Enhanced electrocatalytic biomass oxidation at low voltage by Ni2+-O-Pd interfaces
Source: Nat Commun. 2024 Jul 13;15:5899. doi: 10.1038/s41467-024-50325-w (PMC11246419; doi:10.1038/s41467-024-50325-w)
Supplement: Supplementary file 1 — Supplementary Information [file 41467_2024_50325_MOESM1_ESM.pdf]

Supplementary Information

Enhanced electrocatalytic biomass oxidation at low voltage by Ni^{2+}-O-Pd interfaces

An Pei[1†], Peng Wang[1†], Shiyi Zhang[1], Qinghua Zhang[2], Xiaoyi Jiang[1], Zhaoxi Chen[1], Weiwei Zhou[1], Qizhen Qin[1], Renfeng Liu[1], Ruian Du[1], Zhengjian Li[1], Yongcai Qiu[1], Keyou Yan[1], Lin Gu[2,3*], Jinyu Ye[4], Geoffrey I.N. Waterhouse[5], Wei-Hsiang Huang[6], Chi-Liang Chen[6], Yun Zhao[1*] and Guangxu Chen[1*]

Affiliations:

[1] School of Environment and Energy, State Key Laboratory of Luminescent Materials and Devices, Guangdong Provincial Key Laboratory of Atmospheric Environment and Pollution Control, South China University of Technology; Guangzhou 510006, China.

[2] Institute of Physics, Chinese Academy of Sciences; Beijing 100190, China.

[3] School of Materials Science and Engineering, Tsinghua University; Beijing 100083, China.

[4]College of Chemistry and Chemical Engineering, Xiamen University; Xiamen 361005, China.

[5] School of Chemical Sciences, the University of Auckland; Auckland 1142, New Zealand.

[6] National Synchrotron Radiation Research Center (NSRRC); Hsinchu 30076, Taiwan.

[†]These authors contributed equally to this work.

*Corresponding author.

Email: cgx08@scut.edu.cn (G.C.); yunzhao@scut.edu.cn (Y.Z.); lingu@mail.tsinghua.edu.cn (L.G.)

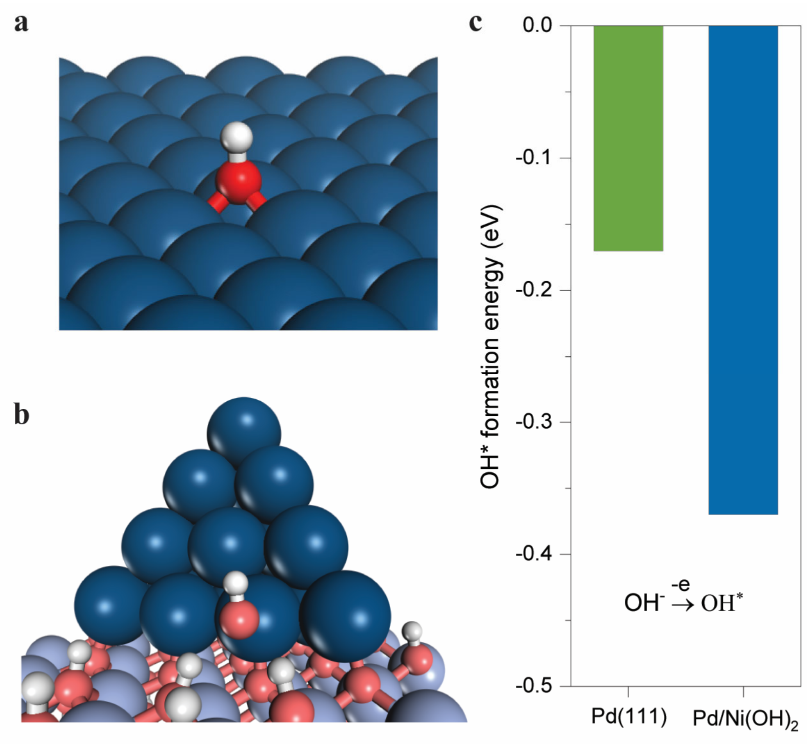

Supplementary Fig. S1 | Formation OH* on pristine Pd(111) and Ni^{2+}-O-Pd interface.
The configurations of OH* adsorption on Pd(111) surface (**a**) and Ni^{2+}-O-Pd interface (**b**), respectively. **c,** Comparison of the OH* free energy changeon the Pd(111) surface and the Ni^{2+}-O-Pd interface.

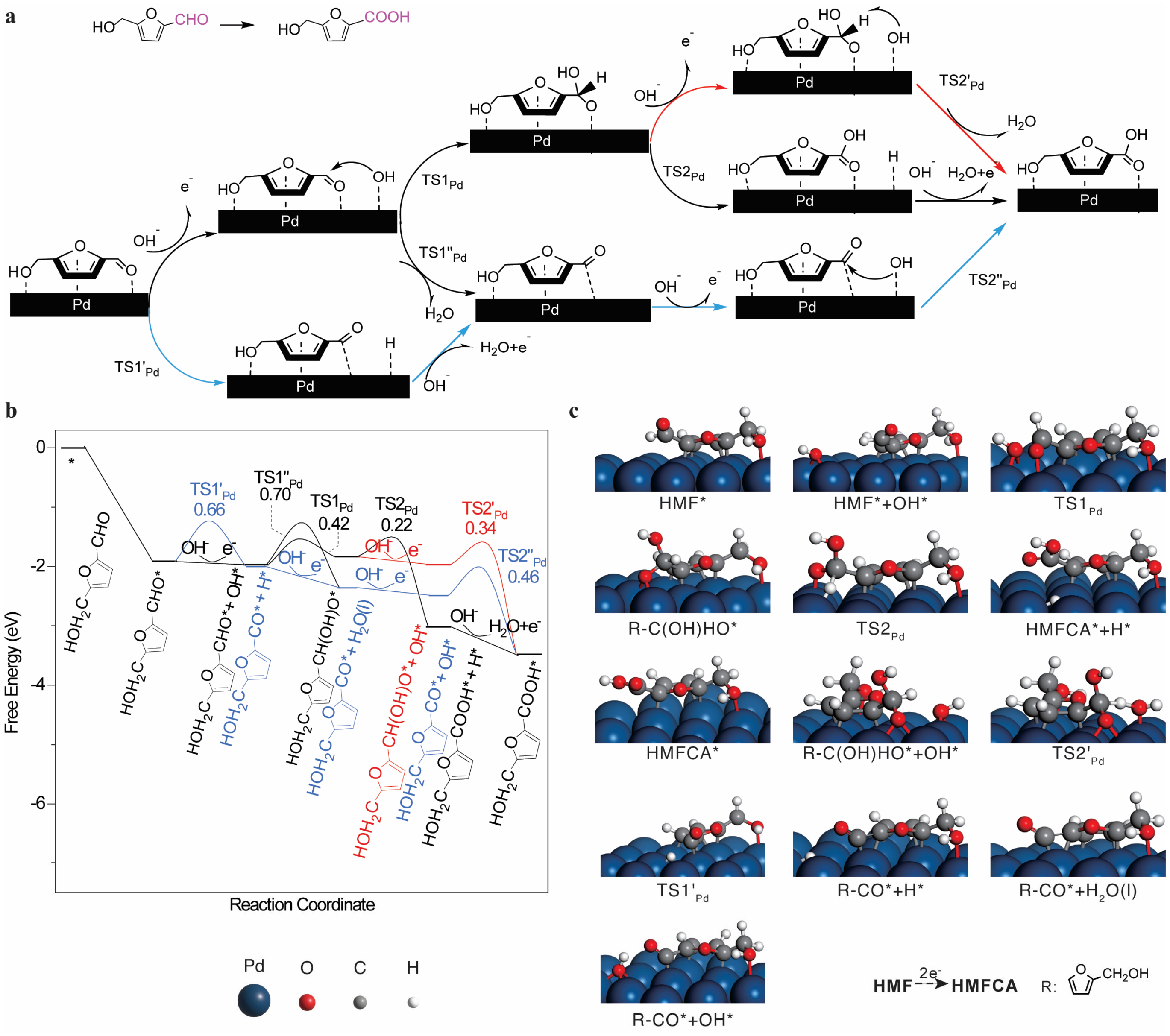

Supplementary Fig. S2 | Electrooxidation of HMF to HMFCA on pristine Pd(111) surface. a, The possible reaction pathways for HMF oxidation to HMFCA on the Pd(111) surface. **b**, The corresponding energies of the intermediates and transition states from DFT calculation. **c**, The corresponding structures of the intermediates and transition states. Typically, the reaction started with the electrooxidation of the aldehyde group (CHO), leading to five possible reaction paths: CHO group was first coupled with OH* and converted to gem-diolate anion intermediate (R-CHO-OH*, where R was furylmethanol group), which was then followed by the reaction of C-H bond scission without (I) or with (II) OH* to generate HMFCA*; HMFCA* can also be produced by the coupling of OH* with R-CO* which was generated from the direct dehydrogenation of R-CHO* (III) to R-CO* or OH*-assisted C-H scission of R-CHO* (IV) to R-CO*. The fifth reaction path was the C-C bond breaking (decarbonylation) of R-CO* with CO* formation (V) (Supplementary Fig. S5). Reaction path I was the most favorable path because the CHO group had the lowest energy barrier for coupling with OH* ($TS1_{Pd}$, 0.42 eV).

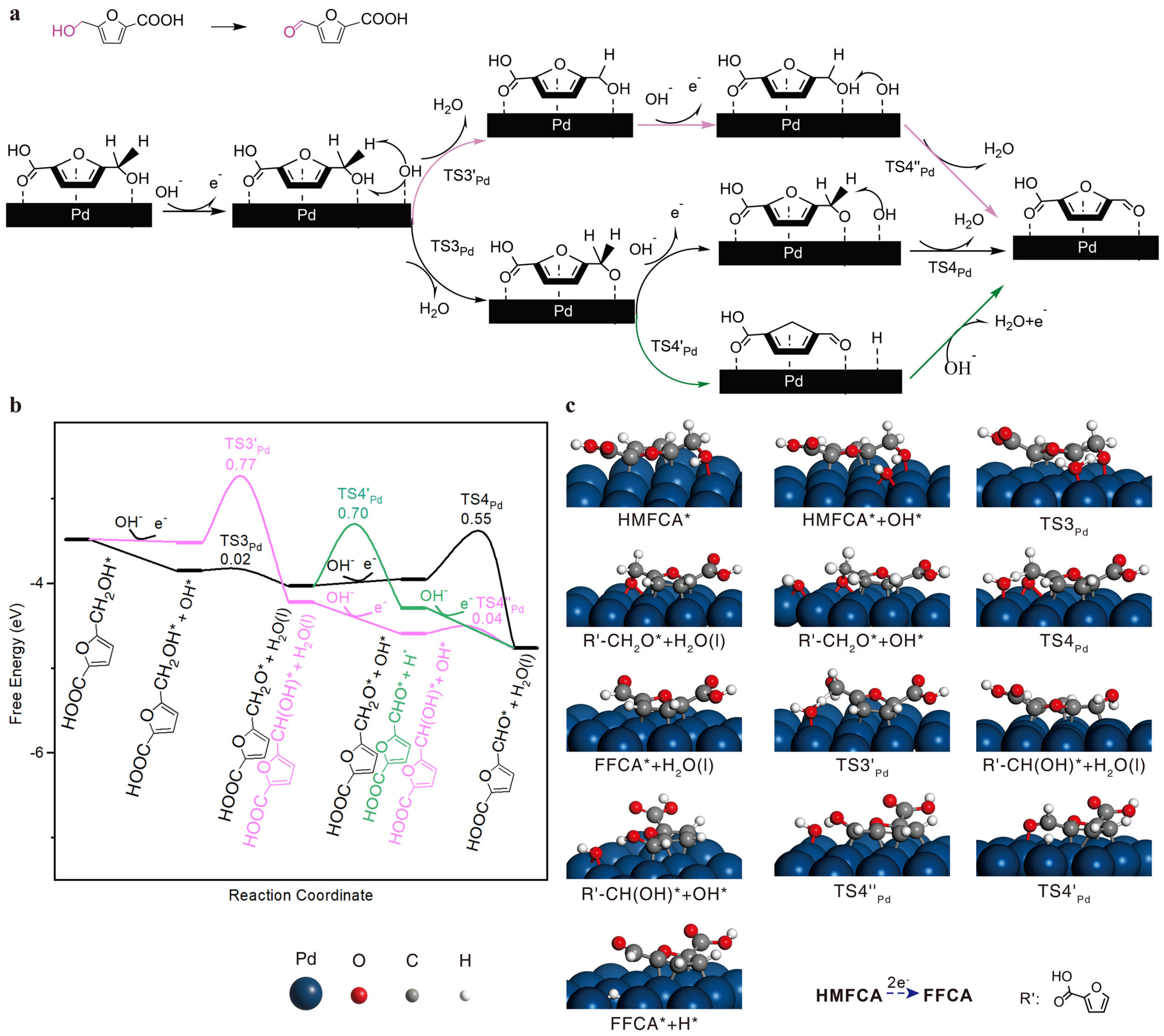

Supplementary Fig. S3 | Electrooxidation of HMFCA to FFCA on pristine Pd(111) surface. a, The possible reaction pathways for HMFCA oxidation to FFCA on the Pd(111) surface. **b,** The corresponding energies of the intermediates and transition states from DFT calculation. **c,** The corresponding structures of the intermediates and transition states.

HMFCA* was oxidized to formylfurancarboxylic acid (FFCA) by surface-bound OH*, with O-H bond activation via proton transfer having a lower activation barrier than C-H bond scission ($TS3_{Pd}$ vs $TS3'_{Pd}$, 0.02 vs 0.77 eV). The generated OH* consecutively attacked the H atoms of R′-CH_2O* (R′ was 2-Furoic acid group) or R′-CH(OH) to produce FFCA with activation barriers of 0.55 eV ($TS4_{Pd}$) and 0.04 eV ($TS4''_{Pd}$), respectively. These indicate that the HMFCA electrooxidation step occurs prior to the elimination of the O-H bond of the hydroxymethyl group under the assistance of OH*.

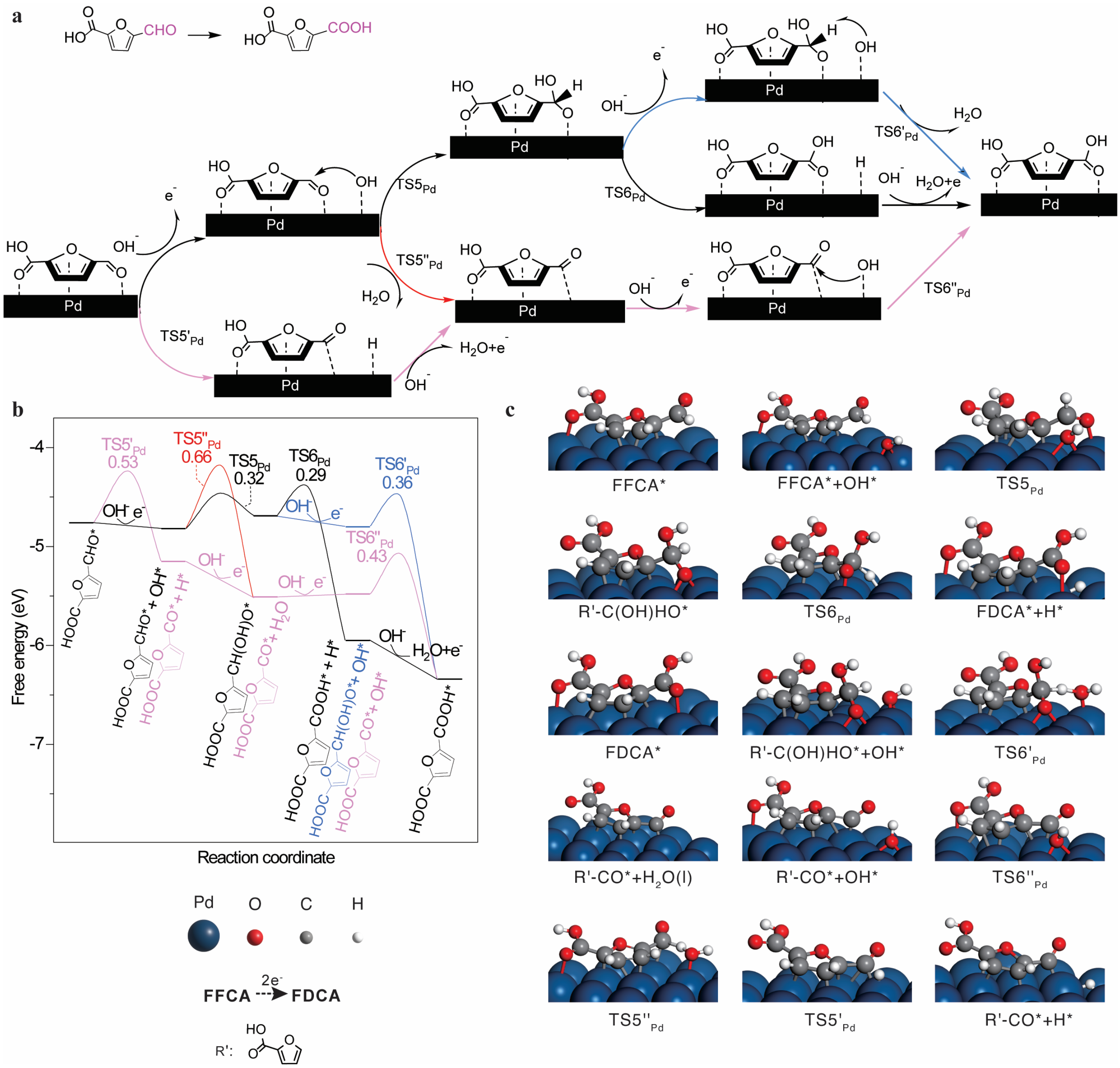

Supplementary Fig. S4 | Electrooxidation of FFCA to FDCA on pristine Pd(111) surface. a, The possible reaction pathways for FFCA oxidation to FDCA on the Pd(111) surface. **b**, The corresponding energies of the intermediates and transition states from DFT calculation. **c**, The corresponding structures of the intermediates and transition states.

The aldehyde group electrooxidation of FFCA follows a similar reaction pathway to HMF oxidation but has a lower energy barrier for coupling CHO with OH* ($TS1_{Pd}$, 0.42 eV vs $TS5_{Pd}$, 0.32 eV).

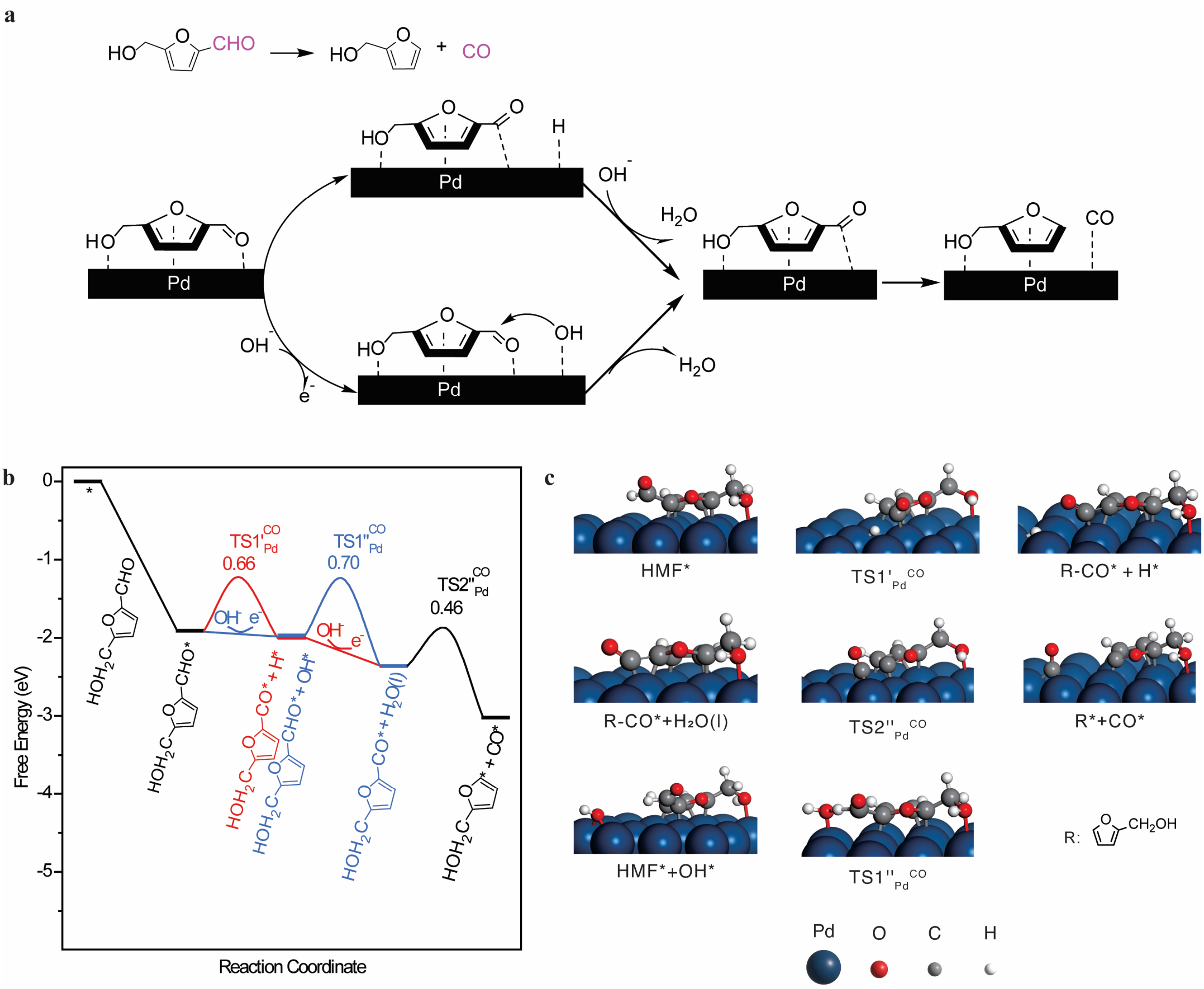

Supplementary Fig. S5 | HMF decarbonylation to generate CO on pristine Pd(111) surface. a, The possible reaction pathways for HMF decarbonylation on the Pd(111) surface. **b**, The corresponding energies of the intermediates and transition states from DFT calculation. **c**, The corresponding structures of the intermediates and transition states.

There are two reaction pathways for HMF decarbonylation to produce the intermediate of R-CO*, which is followed by the C-C bond breaking (decarbonylation) of R-CO* to generate CO* with an energy barrier of 0.46 eV ($TS2''^{CO}_{Pd}$). R-CO* can be generated from the direct dehydrogenation of R-CHO* with an energy barrier of 0.66 eV ($TS1'^{CO}_{Pd}$) or OH*-assisted C-H scission of R-CHO* with an energy barrier of 0.70 eV ($TS1''^{CO}_{Pd}$).

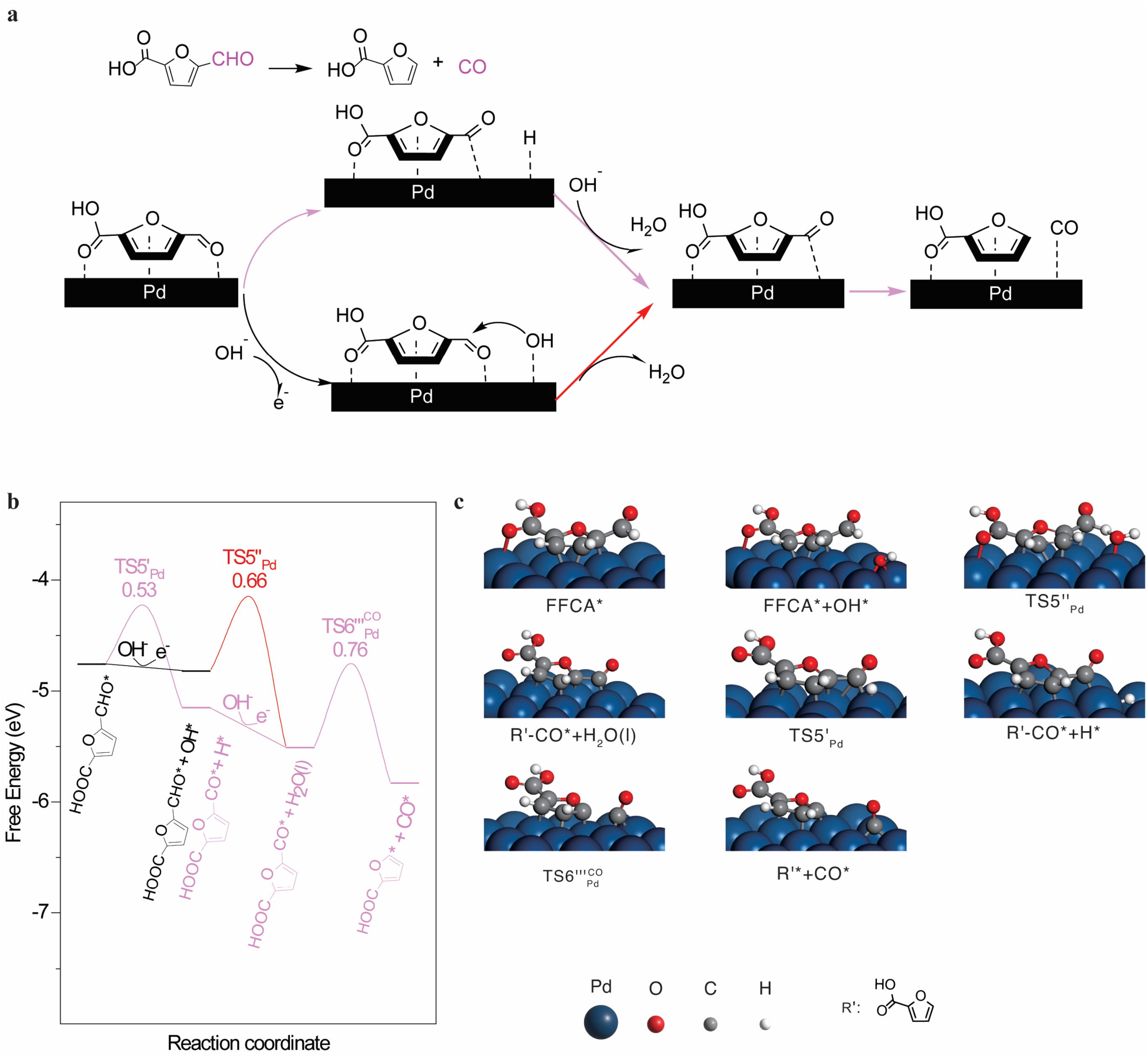

Supplementary Fig. S6 | FFCA decarbonylation to generate CO on pristine Pd(111) surface. a, The possible reaction pathways for FFCA decarbonylation on the Pd(111) surface. **b**, The corresponding energies of the intermediates and transition states from DFT calculation. **c**, The corresponding structures of the intermediates and transition states.

There are also two reaction pathways for FFCA decarbonylation to produce the intermediate of R′-CO*, which is followed by the C-C bond breaking (decarbonylation) of R′-CO* to generate CO* with an energy barrier of 0.76 eV ($TS6'''^{CO}_{Pd}$). R′-CO* can be generated from the direct dehydrogenation of R′-CHO* with energy barrier of 0.53 eV ($TS5'_{Pd}$) or OH*-assisted C-H scission of R′-CHO* with an energy barrier of 0.66 eV ($TS5''_{Pd}$).

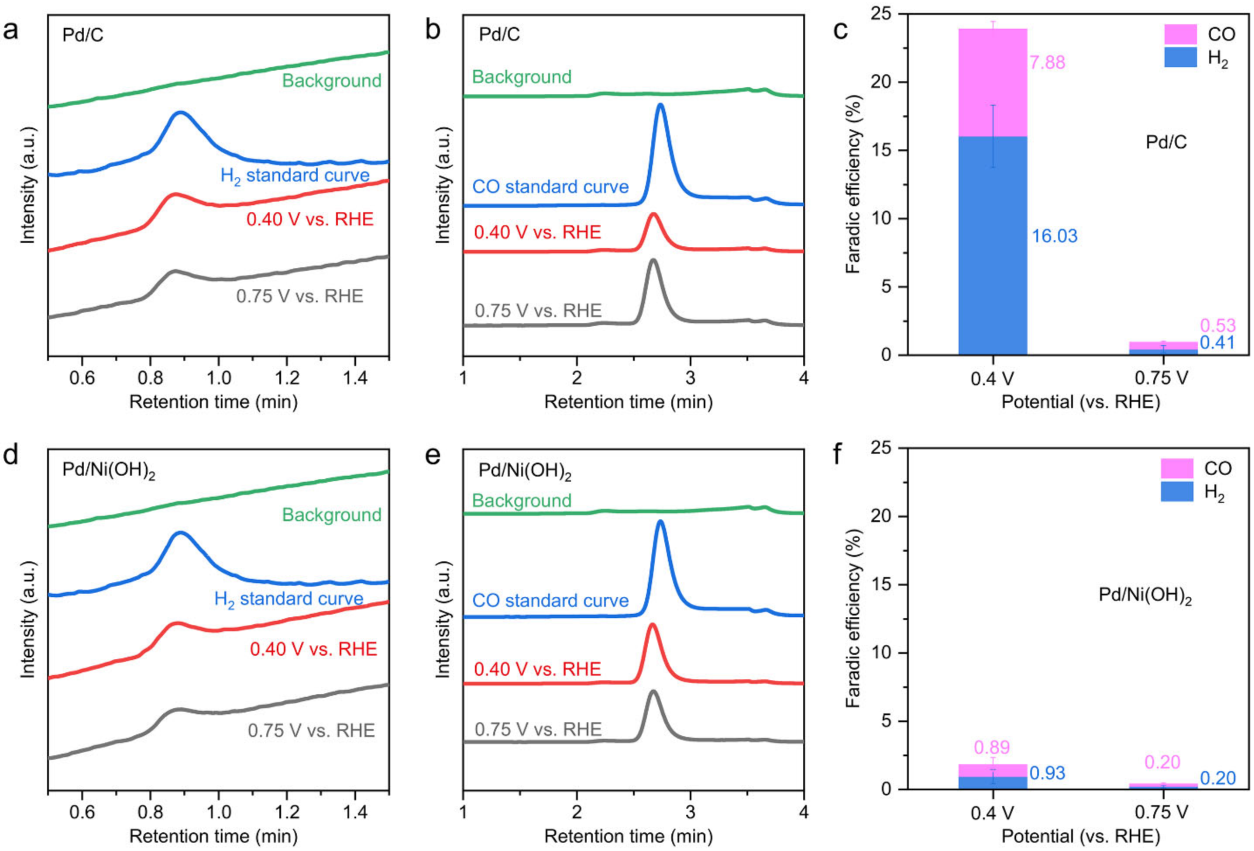

Supplementary Fig. S7 | In-situ detection of CO and H_2 during HMFOR on Pd/C and Pd/Ni(OH)$_2$ catalysts. The generation of CO and H_2 was detected in-situ by gas chromatography during running chronoamperometry (CA) at 0.40 and 0.75 V versus RHE in 1 M KOH solution + 50 mM HMF for HMFOR on both Pd/C and Pd/Ni(OH)$_2$ catalyst. **a**, the GC signal of H_2 from the Pd/C catalyst. **b**, the GC signal of CO from the Pd/C catalyst. **c**, the FE of both CO and H_2 from Pd/C catalyst under different potentials. **d**, the GC signal of H_2 from the Pd/Ni(OH)$_2$ catalyst. **e**, the GC signal of CO from the Pd/Ni(OH)$_2$ catalyst. **f**, the FE of both CO and H_2 from Pd/Ni(OH)$_2$ catalyst under different potentals.

Note: The generation of CO suggested that a decarbonylation reaction occurred during HMFOR on the Pd/C catalyst, potentially leading to the poisoning of the Pd catalyst surface by CO. To ascertain the Faraday efficiency of CO, indicating the actual number of CO molecules formed relative to the expected amount of HMF molecules during the HMFOR experiment, in-situ gas chromatography detection was conducted at different potentials to detect CO. Additionally, the presence of H_2 was also evaluated. Background measurements were initially taken, where no CO was detected during chronoamperometry in a solution of 1 M KOH with 0 mM HMF. In contrast, the intensity of the CO signal increased significantly in the electrolyte containing 1 M KOH with 50 mM HMF, as shown in Supplementary Fig. S7. As shown in Supplementary Fig. S7a-c, the generation of both CO and H_2 were in-situ detected by GC during running chronoamperometry (CA) at 0.40 and 0.75 V versus RHE in 1 M KOH solution + 50 mM HMF for HMFOR on Pd/C and Pd/Ni(OH)$_2$ catalysts. The Faradaic efficiency of both CO and H_2 at 0.40 V is higher than at 0.75 V in a solution of 1 M KOH + 50 mM HMF for HMFOR on both Pd/C and Pd/Ni(OH)$_2$ catalyst. It is noteworthy that the Faradic efficiency of CO generation reaction on Pd/Ni(OH)$_2$ ($FE_{(CO)}$=0.89% at 0.4 V, $FE_{(CO)}$=0.20% at 0.75 V) catalyst was much lower than that on Pd/C ($FE_{(CO)}$=7.88% at 0.4 V, $FE_{(CO)}$=0.53% at 0.75 V). This demonstrates that the CO generation reaction on Pd was suppressed by the formation of the Ni^{2+}-O-Pd interface, as depicted in Supplementary Fig. S8d-f.

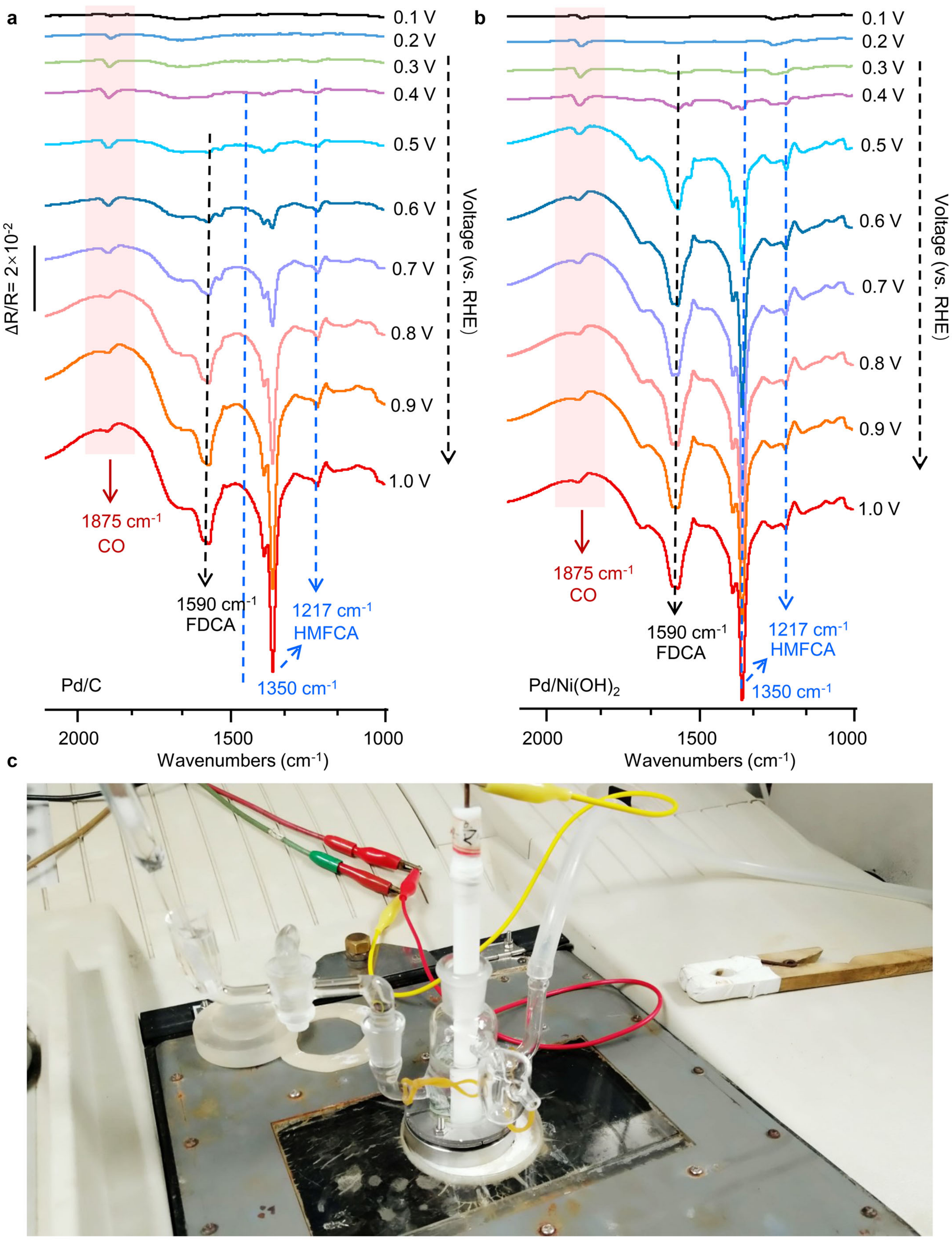

Supplementary Fig. S8 | Electrochemical in-situ FTIR studies on Pd/C and Pd/Ni(OH)$_2$ catalysts during HMFOR. a, b, In-situ FTIR spectra of Pd/C (**a**) and Pd/Ni(OH)$_2$ (**b**) during HMFOR, respectively. **c,** Figure of the experimental set-up.

Note: During chronoamperometry at different potentials (from 0.1 ~ 1.0 V versus RHE) (with 0.1 V intervals) in 1 M KOH solution containing HMF, in-situ FTIR analysis demonstrated the production of CO

species (1875 cm^{-1}) on both Pd/C and Pd/$Ni(OH)_2$ catalysts, indicating that the decarbonylation reaction occurred on both Pd/C and Pd/$Ni(OH)_2$ catalysts. Moreover, the adsorption of intermediates and the formation of FDCA products during different voltages were further identified. As shown in Supplementary Fig. S8 and referenced to the preliminary study[8], the adsorption of HMFCA intermediate (1217 cm^{-1}, 1350 cm^{-1}) and formation of FDCA (1590 cm^{-1}) were observed on both Pd/C and Pd/$Ni(OH)_2$ catalysts. It is worth noting that the adsorption signal of HMFCA intermediate (1217 cm^{-1}, 1350 cm^{-1}) was firstly observed at a lower voltage of 0.3 V on Pd/$Ni(OH)_2$ catalyst than on the Pd/C catalyst (beginning at 0.4 V), illustrating the higher HMFOR efficient on Pd/$Ni(OH)_2$ catalyst than Pd/C catalyst. Additionally, the product signal of FDCA (1590 cm^{-1}) also emerged at 0.3 V on the Pd/$Ni(OH)_2$ catalyst, indicating that the oxidation of HMFCA was more facile compared to Pd/C, a finding consistent with the results obtained from HPLC analysis. The signal intensity of HMFCA and FDCA were enhanced by increasing HMFOR voltage, illustrating that HMFOR was a potential dependent electrooxidation reaction. The absence of the FFCA intermediate detection, consistent with the HPLC findings, can be attributed to its susceptibility to oxidation on the Pd catalyst during HMFOR.

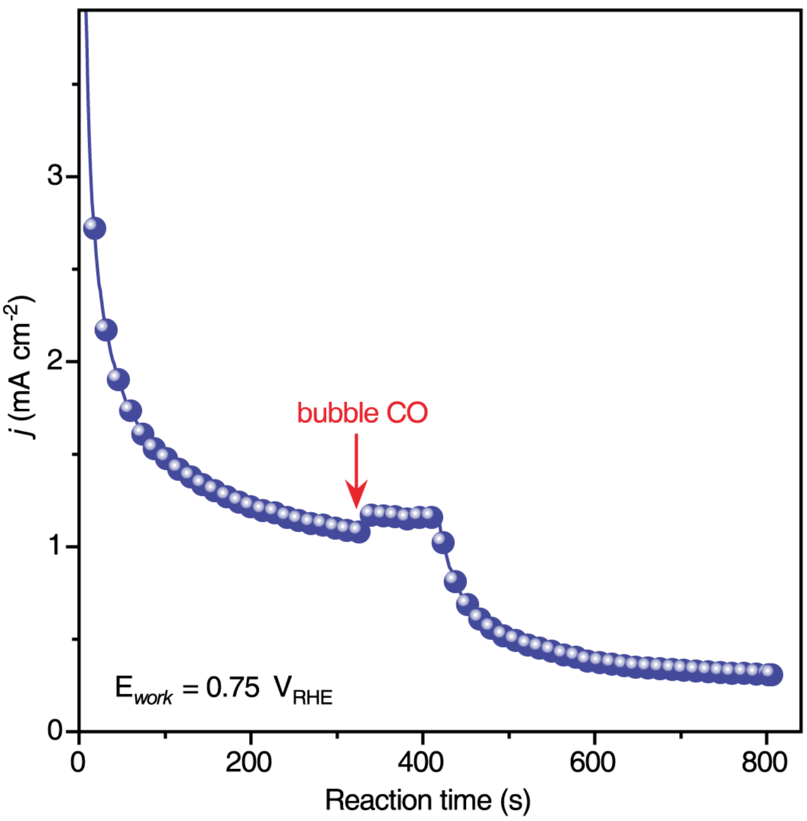

Supplementary Fig. S9 | CO poisoning experiment for HMF electrooxidation reaction on Pd/C in 1M KOH + 5 mM HMF. During the chronoamperometric measurements at 0.75 V versus RHE with 95% iR corrected, CO gas was bubbled through the electrolyte at the time indicated by the arrows. After CO bubbling for a short time, the current density of the HMF electrooxidation reaction dramatically decreased, indicating CO poisoning occurred on Pd/C.

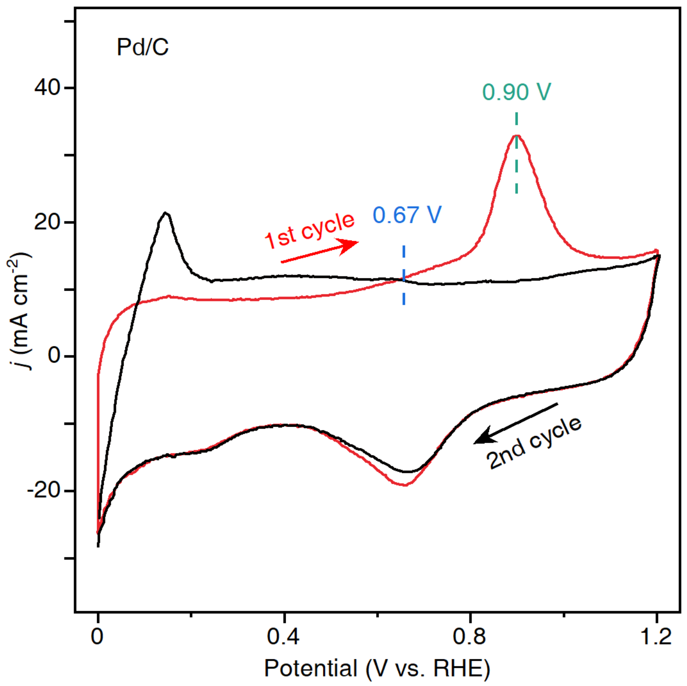

Supplementary Fig. S10 | CO-stripping experiment performed on Pd/C. The CO-stripping experiment evaluated the oxidation potential for CO removal on the Pd surface with 95% iR corrected. CO stripping experiment revealed that CO can be removed from the Pd surface at potentials above 0.67 V versus RHE.

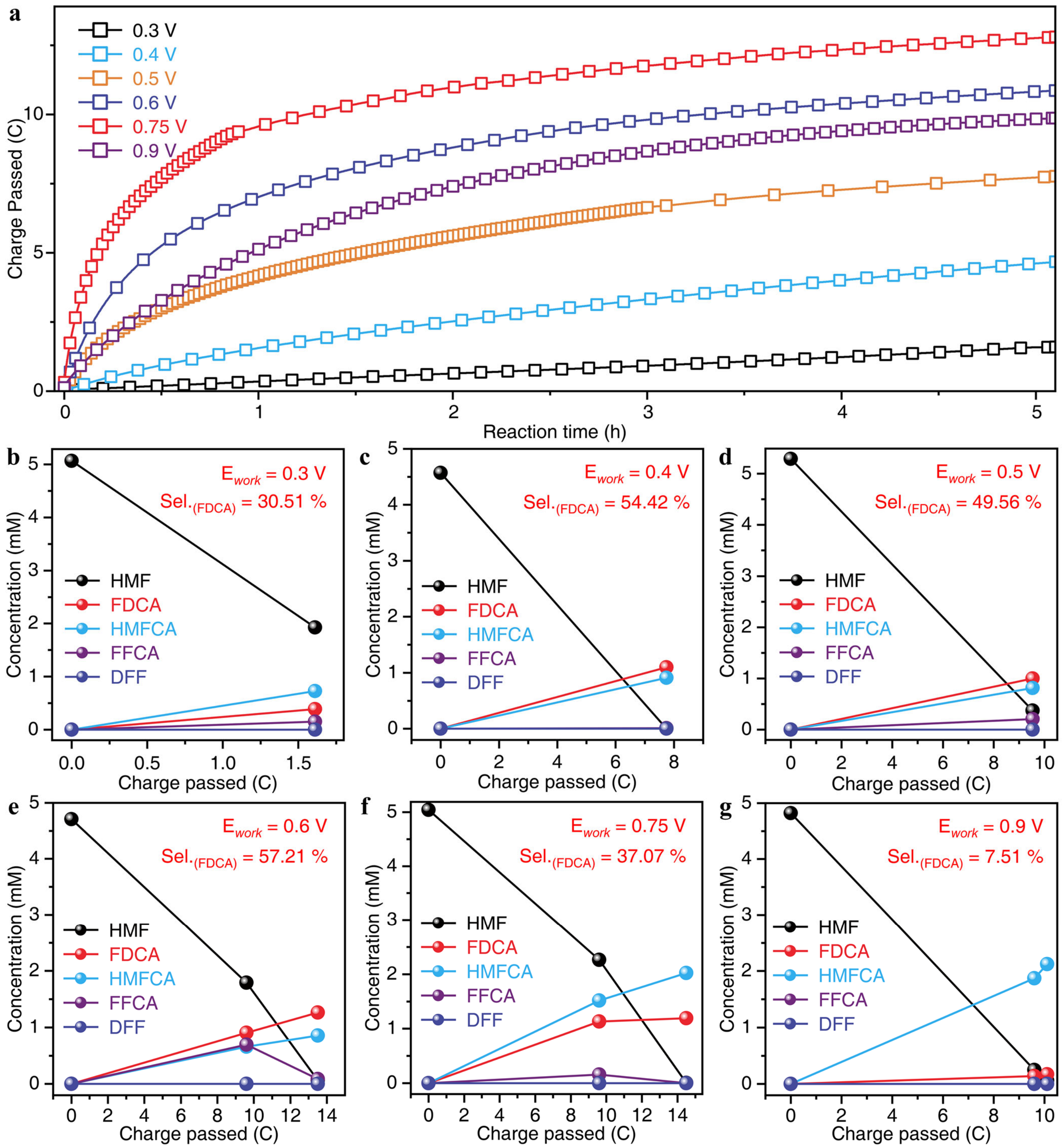

Supplementary Fig. S11 | HMFOR performance on Pd/C catalyst under different oxidation potentials. **a,** Charge consumption on Pd/C catalyst under different oxidation potentials in Ar-saturated 1 M KOH + 5 mM HMF. The curves of charge passed (Q) versus reaction time (t) at each working potential were collected till the end of the reaction (indicated by the reaction current approaching zero). Concentration versus passed charge plots for HMF, FDCA, and the reaction intermediates such as HMFCA, DFF, and FFCA during HMFOR on Pd/C under different potentials: **b,** 0.3 V, **c,** 0.4 V, **d,** 0.5 V, **e,** 0.6 V, **f,** 0.75 V, **g,** 0.9 V versus RHE.

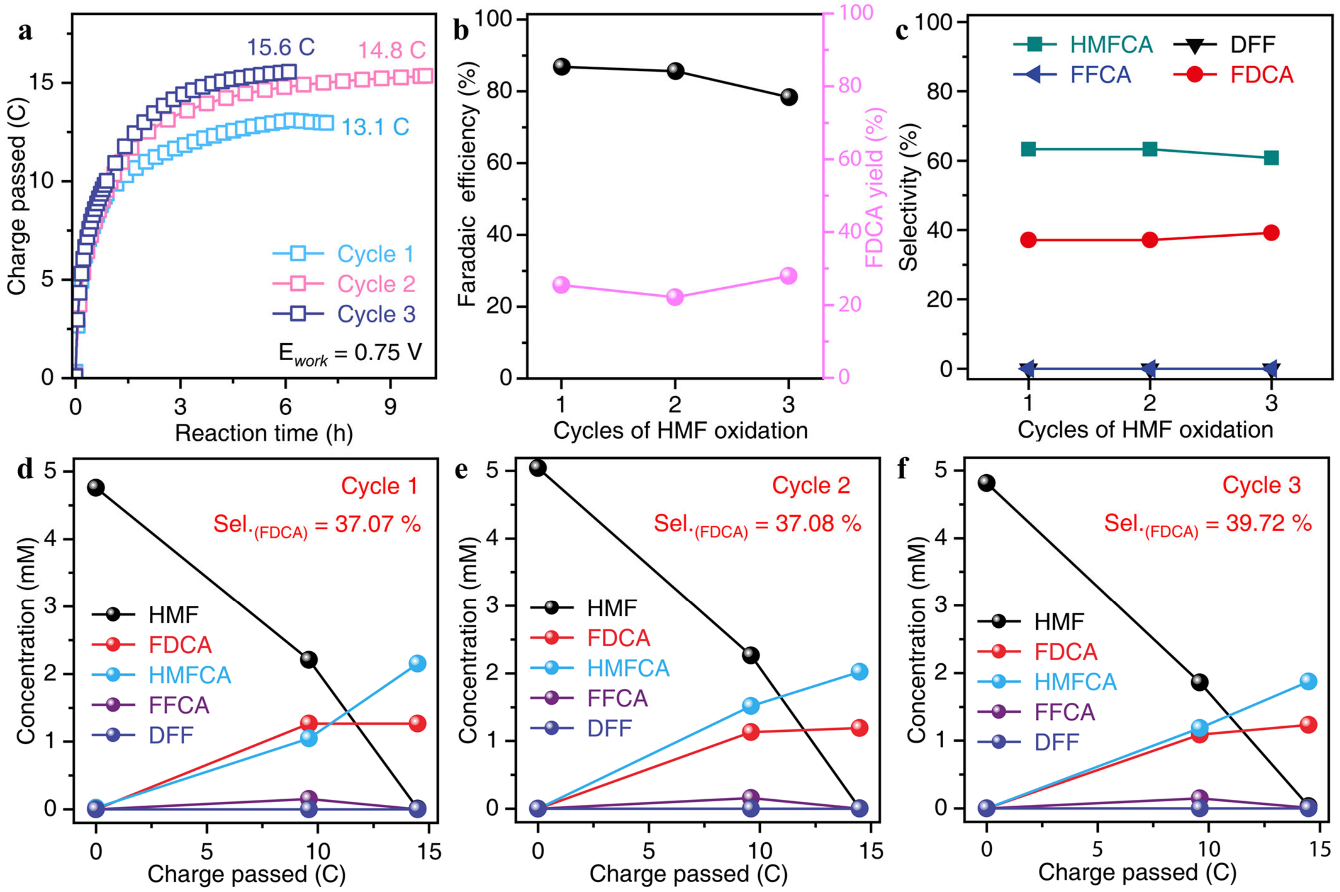

Supplementary Fig. S12 | Recycle stability test and product analysis for HMFOR on Pd/C at 0.75 V versus RHE. a, Charge passed to Pd/C at 0.75 V versus RHE over 3 cycles. **b,** Faradaic efficiency and FDCA yield for HMF OR over Pd/C at 0.75 V versus RHE during the 3-cycle test. **c,** Selectivity of HMFCA, DFF, FFCA, and FDCA over the 3-cycle test. Plots of concentration versus charge passed for HMF, FDCA, and the intermediates such as HMFCA, DFF, and FFCA for HMFOR at 0.75 V versus RHE on Pd/C. **d,** Cycle 1; **e,** Cycle 2; **f,** Cycle 3 in Ar-saturated 1 M KOH + 5 mM HMF electrolyte.

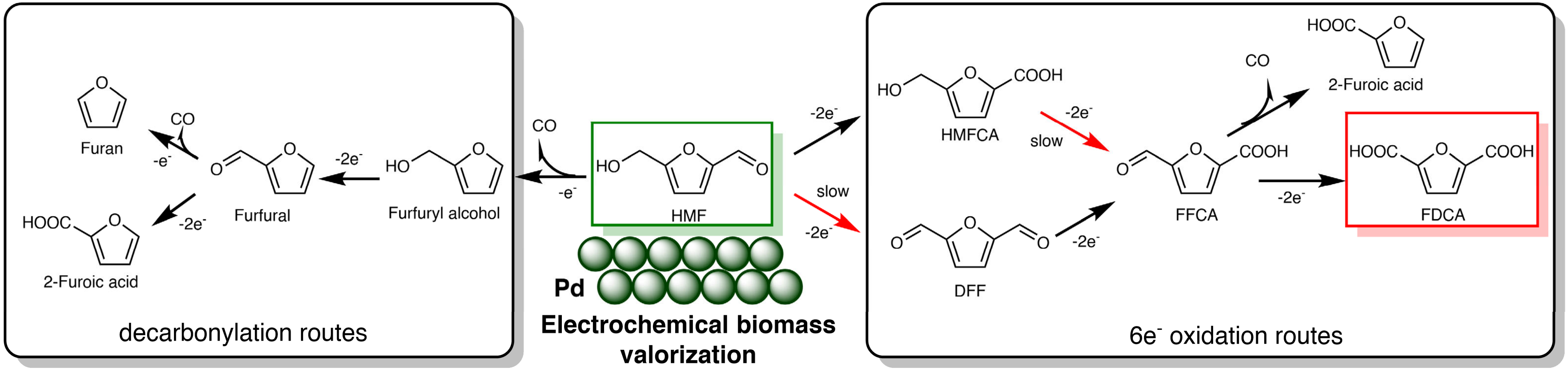

Supplementary Fig. S13 | Possible reaction pathways for HMFOR on Pd/C. In addition to the classical 6 electron transfer pathways reported to FDCA, the decarbonyl pathway also occurred on the Pd/C electrode during the HMF electrooxidation reaction. The generation of CO was confirmed by gas chromatography (Supplementary Fig. S7).

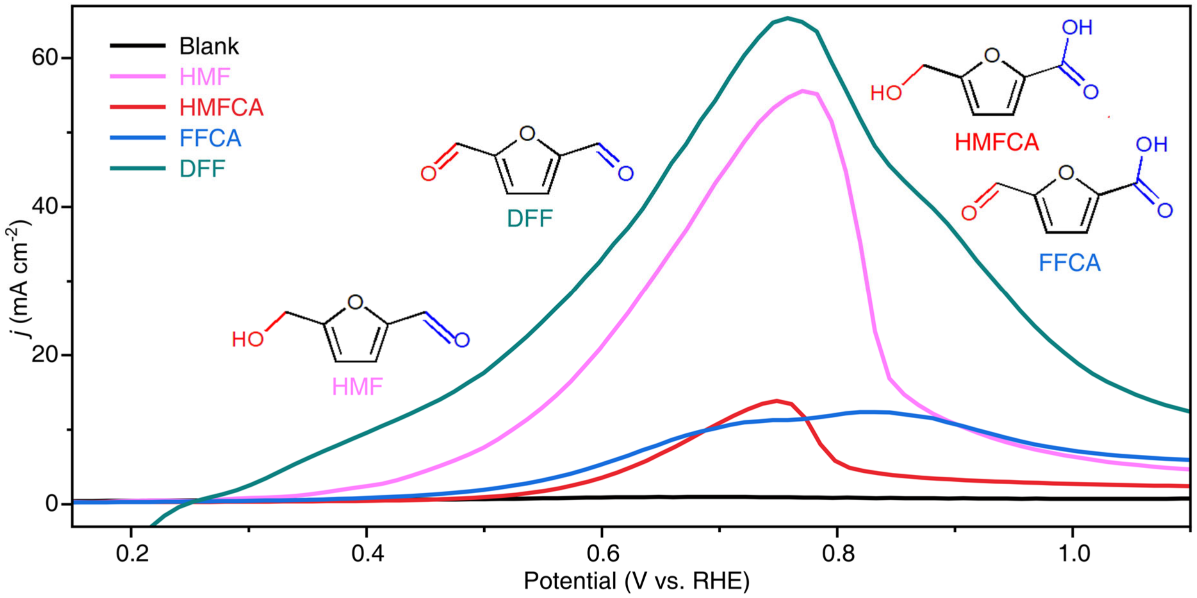

Supplementary Fig. S14 | Polarization curves of Pd/C catalyst for electrooxidation of HMF, HMFCA, FFCA, and DFF. The electrooxidation activity of Pd/C for HMF, HMFCA, DFF, and FFCA was tested in an Ar-saturated 1 M KOH + 50 mM substance (HMF, HMFCA, DFF, and FFCA) solution and compared with data collected by using an Ar-saturated 1 M KOH solution with 95% iR corrected.

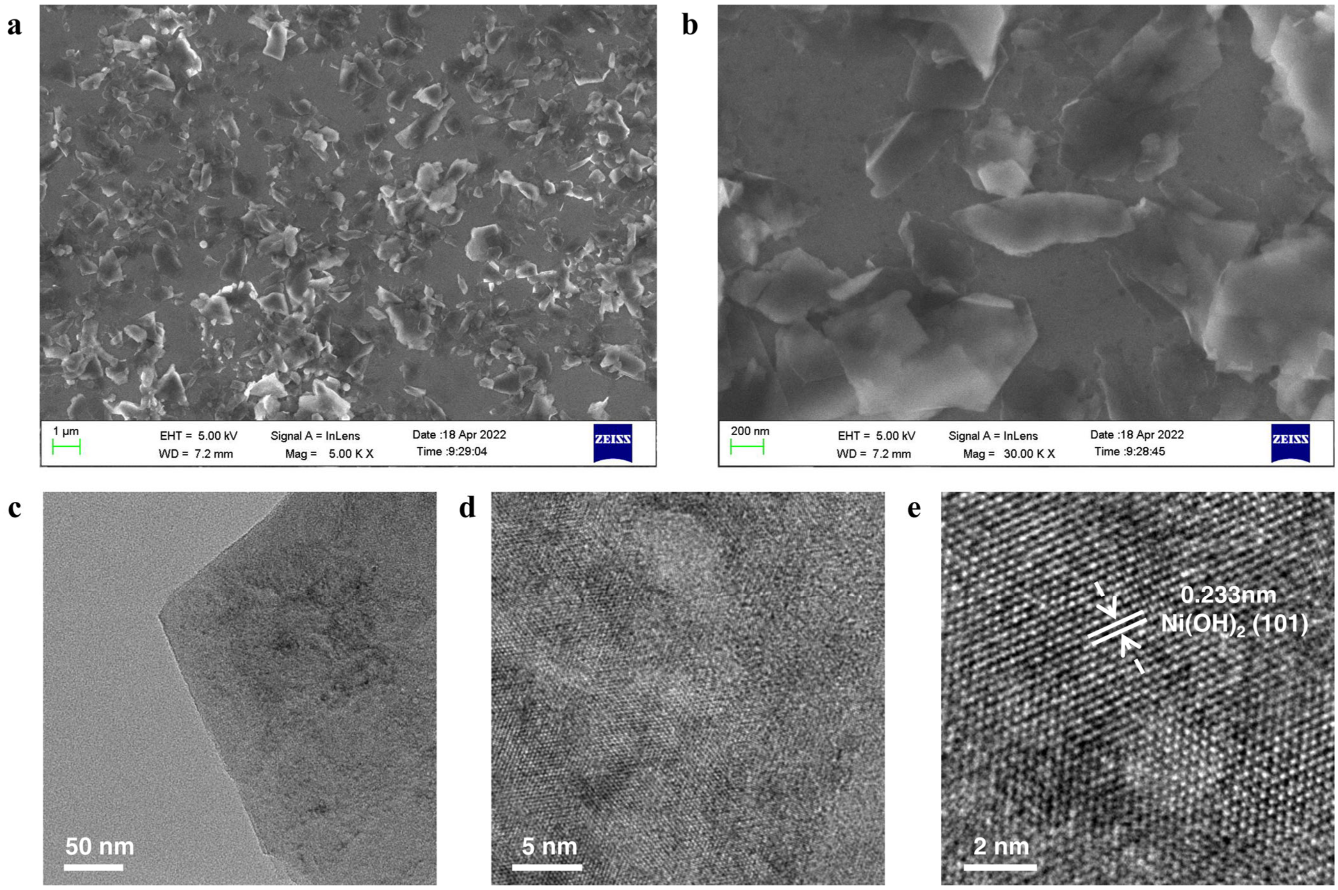

Supplementary Fig. S15 | Characterization of the $Ni(OH)_2$ nanosheets. a, **b**, Representative SEM images of the $Ni(OH)_2$ nanosheets. **c**-**e**, Representative TEM and HRTEM images of $Ni(OH)_2$ nanosheets.

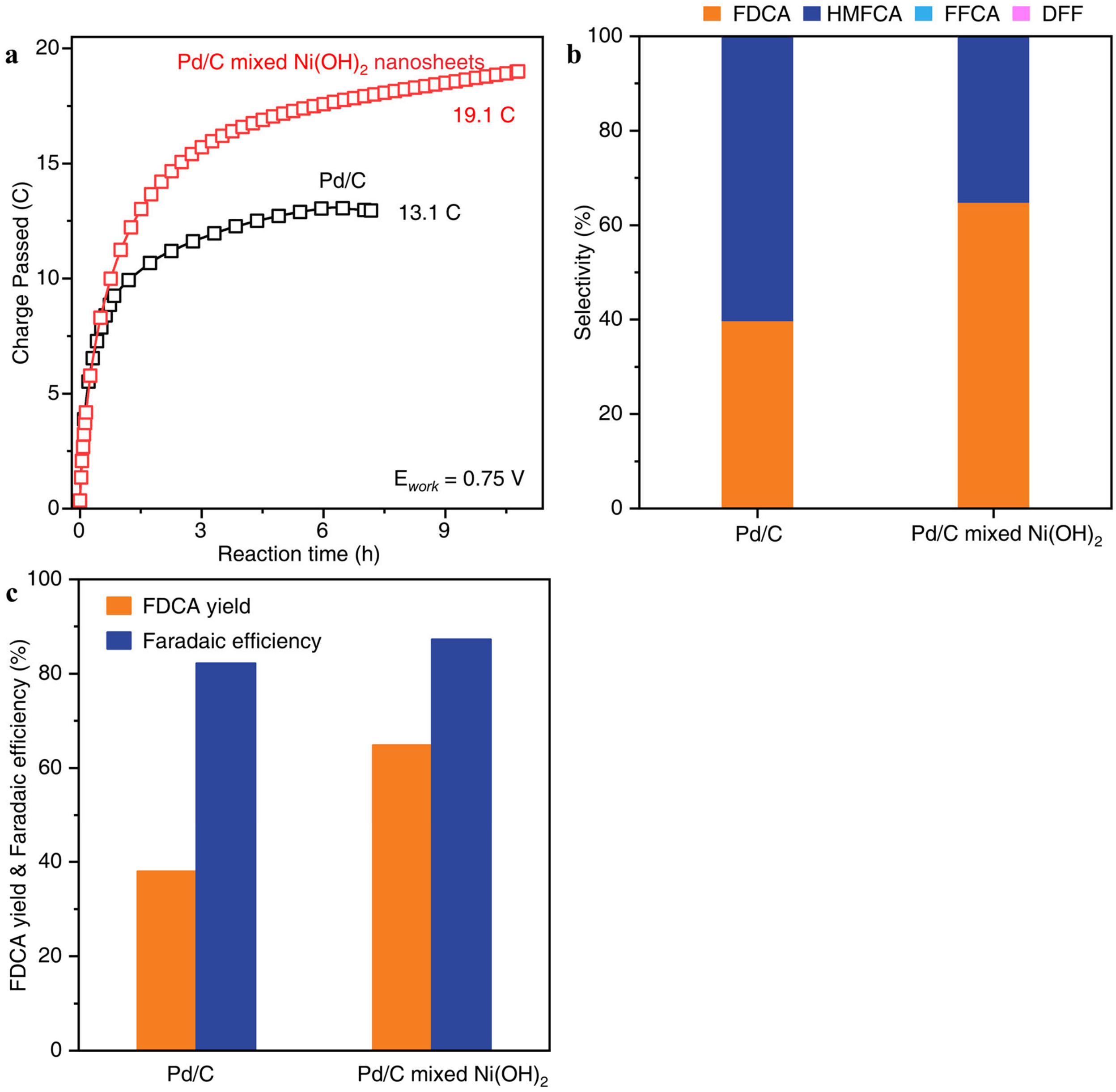

Supplementary Fig. S16 | Comparison of HMFOR over Pd/C and Pd/C physically mixed with $Ni(OH)_2$ nanosheets at 0.75 V versus RHE. a, Curves of charge passed (Q) versus reaction time (t) at 0.75 V versus RHE during HMFOR in Ar-saturated 1 M KOH+ 5 mM HMF solution. **b,** Comparison of the selectivity to HMFCA, DFF, FFCA, and FDCA over different catalysts. **c,** FDCA yield and Faradaic efficiency of Pd/C and Pd/C mixed with $Ni(OH)_2$ nanosheets during HMFOR at 0.75 V versus RHE. The Pd/C physically mixed with $Ni(OH)_2$ nanosheets showed enhanced HMFOR performance, including FDCA selectivity, FE, and FCDA yield.

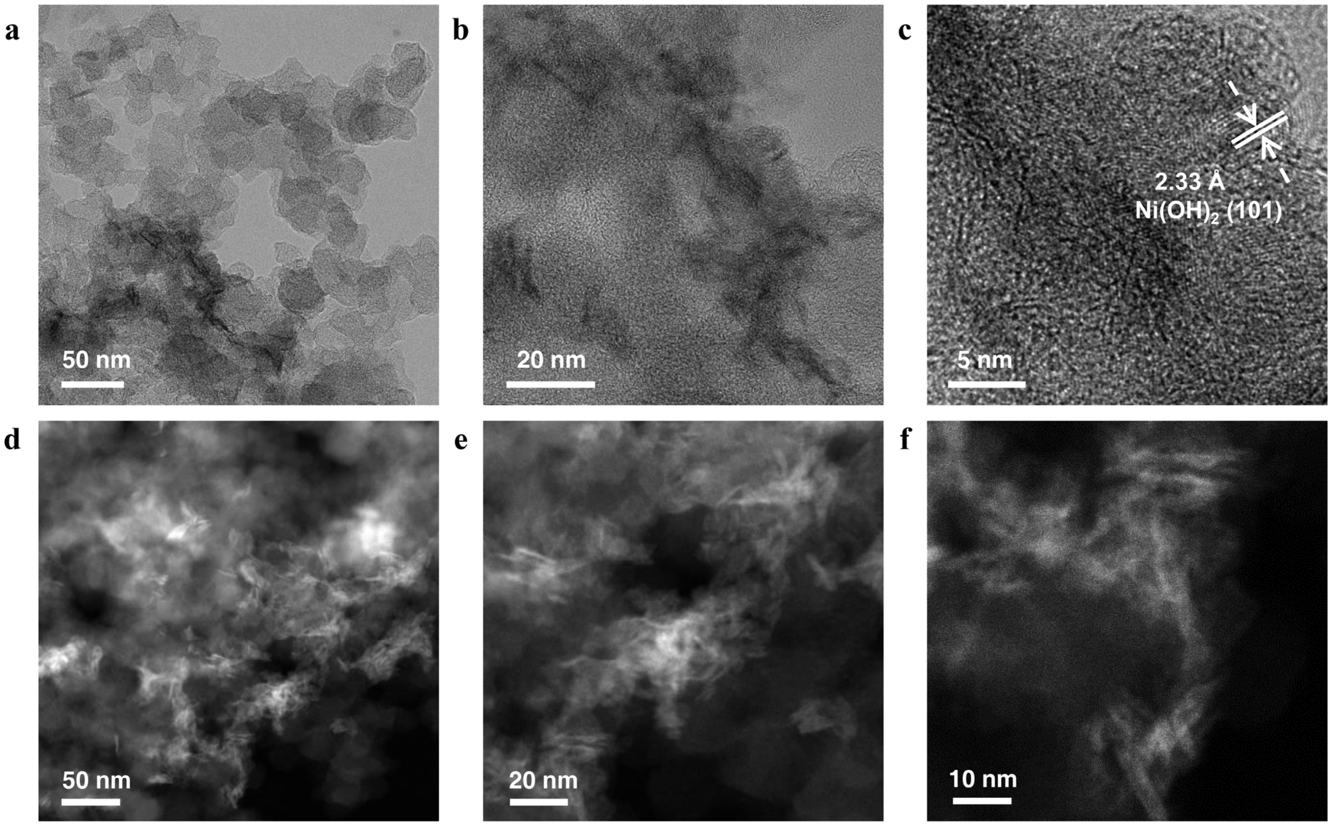

Supplementary Fig. S17 | Structural characterization of thin $Ni(OH)_2$ nanosheets deposited on carbon black. a-c, Representative TEM and HRTEM images of $Ni(OH)_2$ nanosheets. **d-f,** Representative HADDF-STEM images of $Ni(OH)_2$ nanosheets.

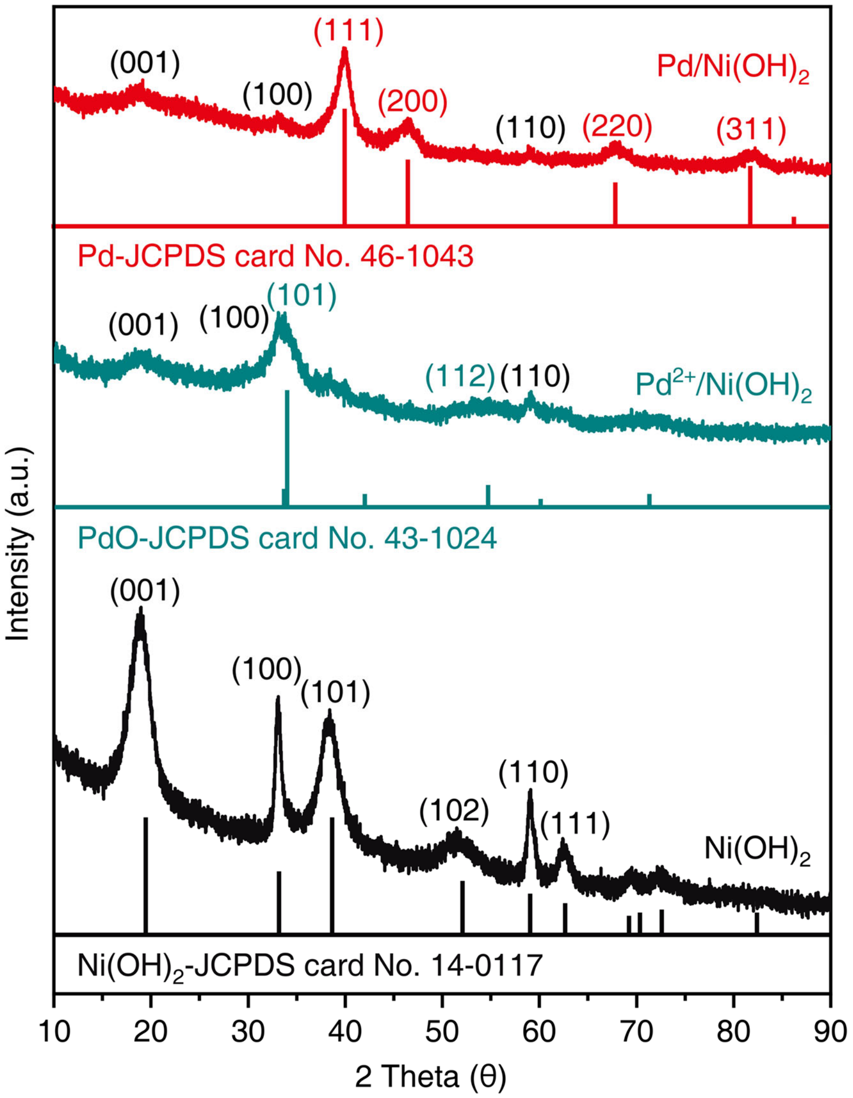

Supplementary Fig. S18 | X-ray diffraction patterns for $Ni(OH)_2$, $Pd^{2+}/Ni(OH)_2$, and $Pd/Ni(OH)_2$ catalysts.

The $Ni(OH)_2$ nanosheets deposited on carbon black showed strong and representative diffraction peaks (bottom) of (001), (100), (101), (110), and so on. After being reacted with the solution of sodium tetrachloropalladate, the diffraction peaks of $Ni(OH)_2$ were lost or weakened, indicating that the $Ni(OH)_2$ nanosheets were etched or leached to the thin $Ni(OH)_2$ species.

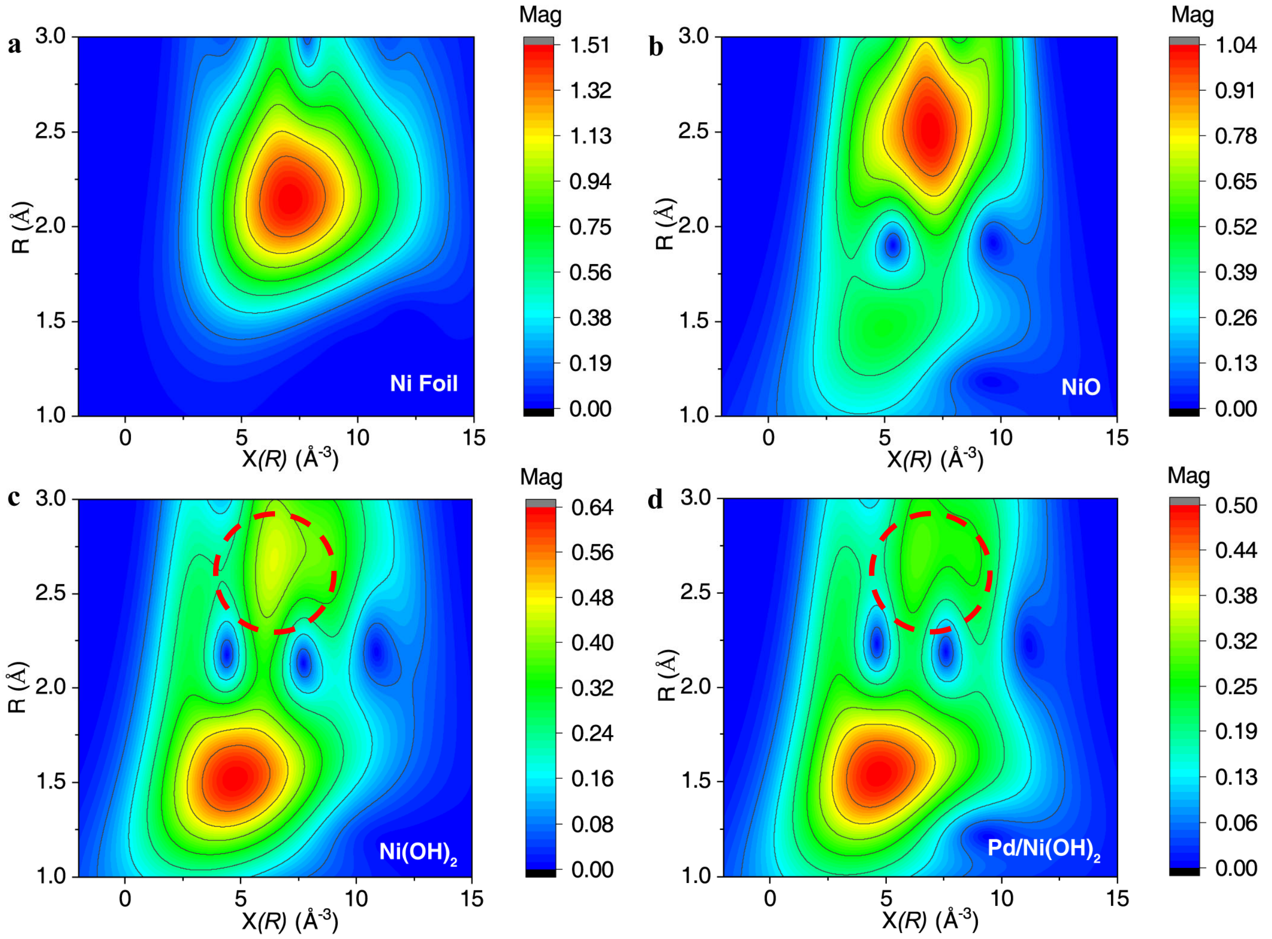

Supplementary Fig. S19 | Wavelet transform of Ni K-edge. Ni K-edge k^2-weighted wavelet transformed contour maps for **a,** Ni foil, **b,** NiO, **c,** $Ni(OH)_2$, and **d,** Pd/$Ni(OH)_2$. Compared with pristine $Ni(OH)_2$, the wavelet transform intensity of Ni K-edge at R = 2.75 Å (in the red dotted circle) is significantly reduced, indicating that Ni-Ni scattering becomes weaker, revealing that $Ni(OH)_2$ was etched into thin $Ni(OH)_2$.

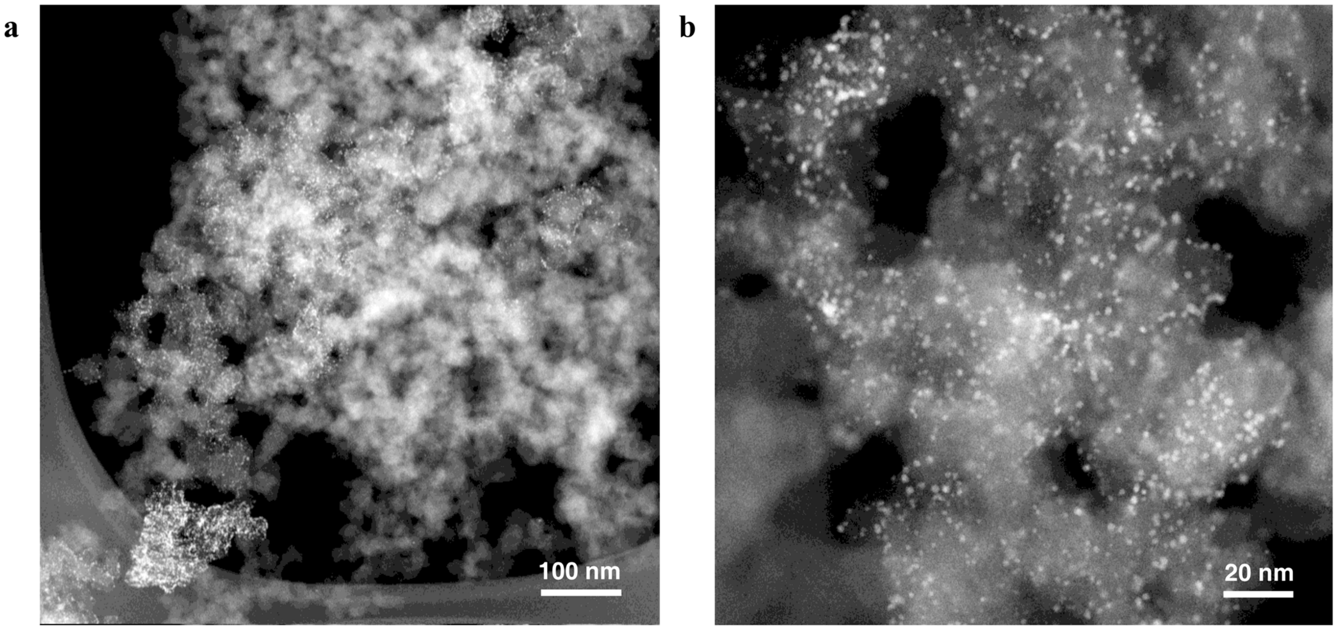

Supplementary Fig. S20 | Probe-corrected STEM images of Pd/Ni(OH)$_2$ catalyst. a, b, Representative STEM images of Pd/Ni(OH)$_2$ catalyst on a large scale. As shown in the STEM images above, the Pd nanoparticles were well-dispersed on Ni(OH)$_2$/C with a size < 2nm.

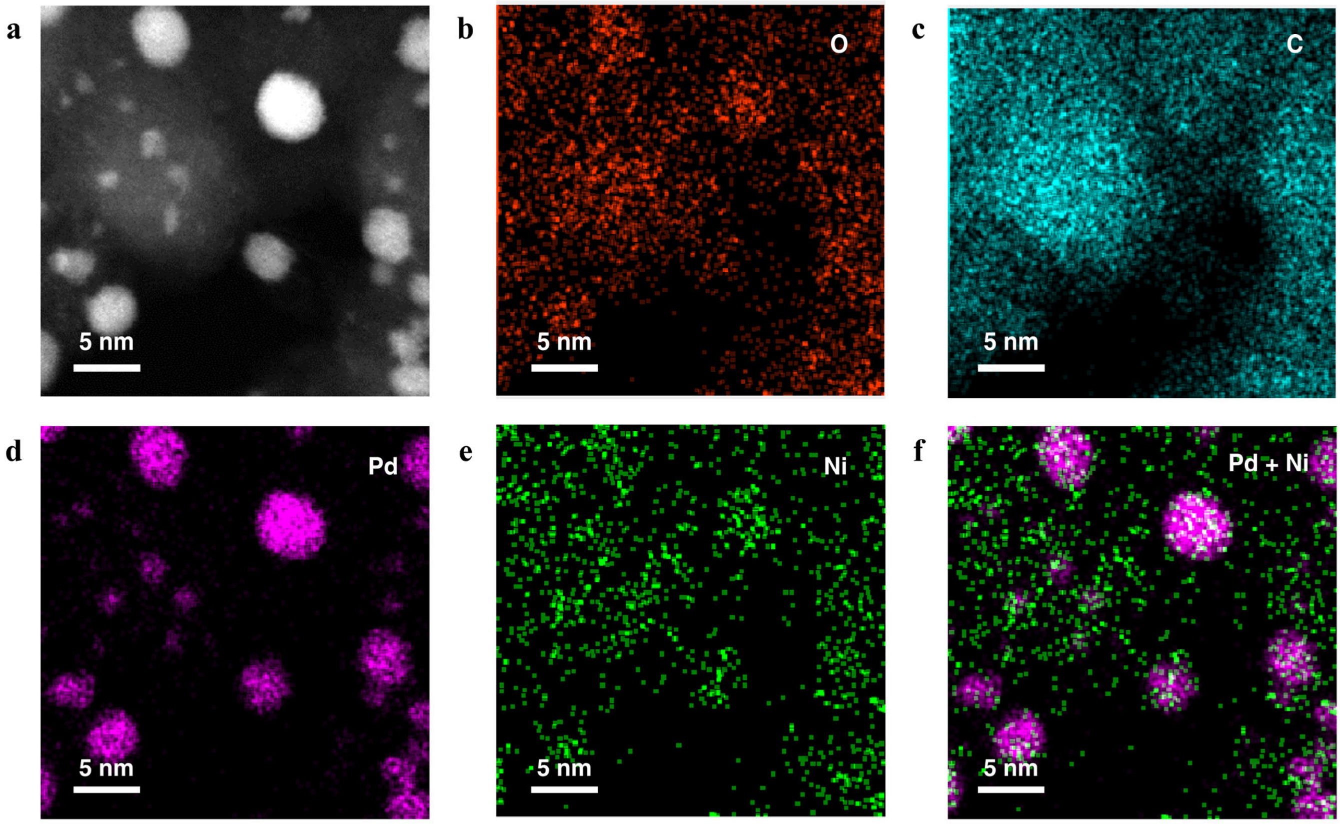

Supplementary Fig. S21 | Energy dispersive spectroscopy mapping of Pd/Ni(OH)$_2$ catalyst on a large scale. a, STEM image. **b**, O. **c**, C. **d**, Pd. **e**, Ni. **f**, overlap of **d** and **e**.

Energy dispersive spectroscopy analysis showed that Pd NPs were in intimate contact with the thin Ni(OH)$_2$, forming abundant Ni^{2+}-O-Pd interfaces.

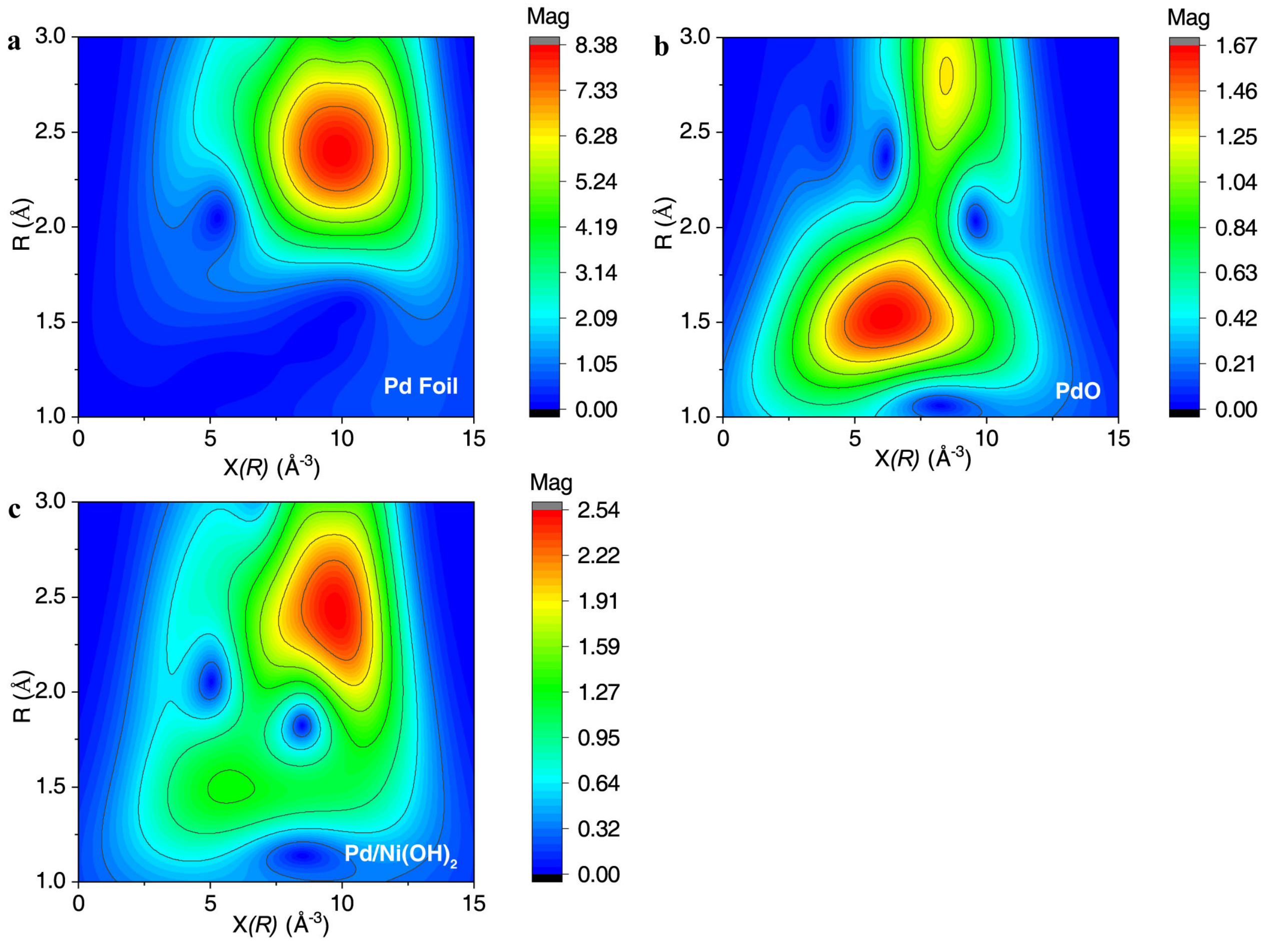

Supplementary Fig. S22 | Wavelet transform of Pd K-edge. Pd K-edge k^2-weighted wavelet transformed contour maps for **a,** Pd foil, **b,** PdO, and **c,** Pd/Ni(OH)$_2$. Compared with Pd and PdO, the wavelet transform of the Pd K-edge of Pd/Ni(OH)$_2$ reveals that Pd^0 was the dominant palladium species in the Pd/Ni(OH)$_2$ catalyst, with part of palladium in the form of a $Pd^{\delta+}$ species. We associate this $Pd^{\delta+}$ species with Ni^{2+}-O-Pd interfaces.

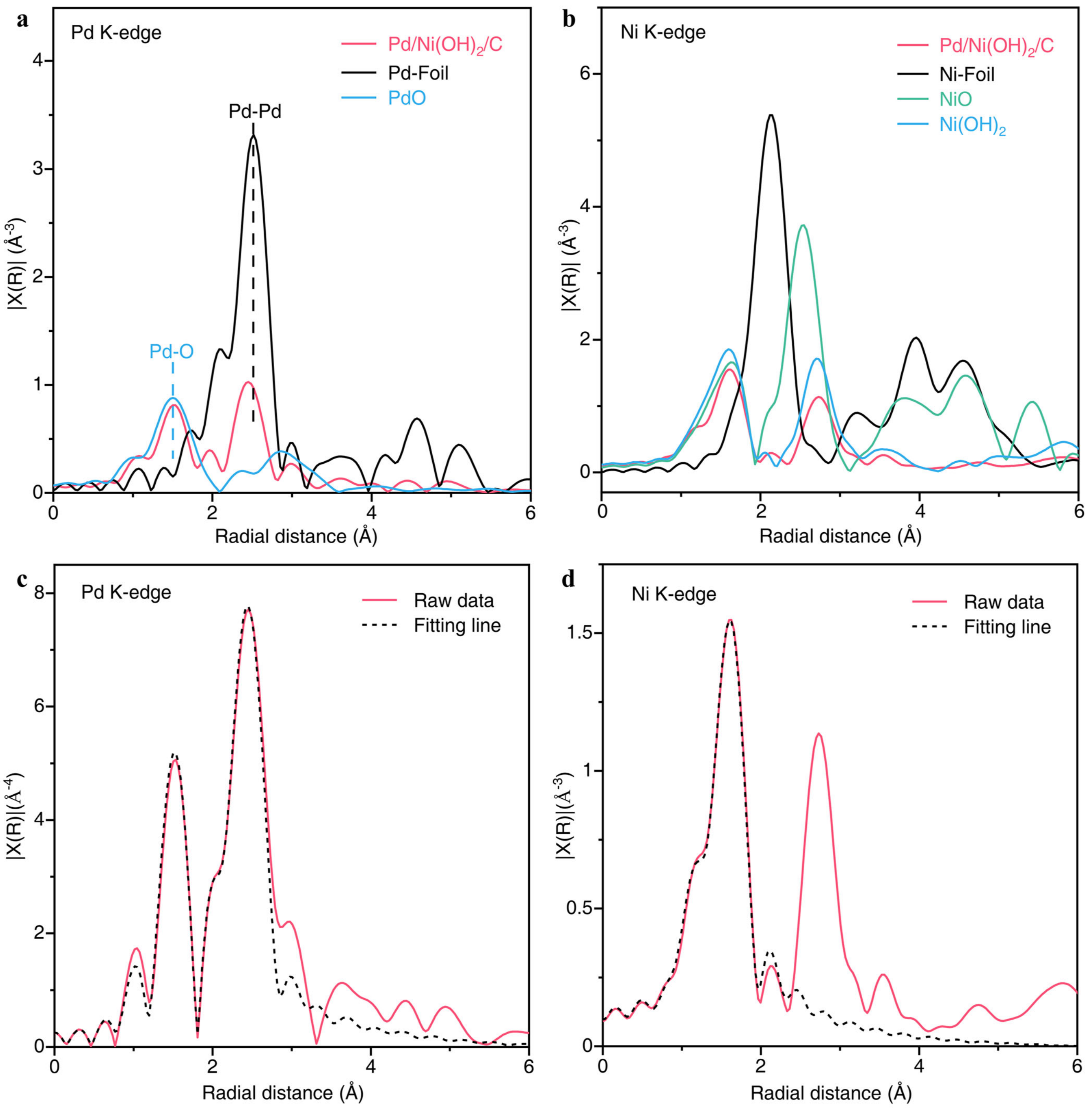

Supplementary Fig. S23 | Extended X-ray absorption fine structure (EXAFS) of Pd/$Ni(OH)_2$ catalyst. **a,** Pd *K*-edge. **b,** Ni *K*-edge of the Pd/$Ni(OH)_2$ catalyst. **c, d** show R spaces of Pd *K*-edge and Ni *K*-edge for Pd/$Ni(OH)_2$ catalyst and the fitting of scattering paths. The Pd *K*-edge spectrum in (**c**) was fitted by a mixed Pd-O and Pd-Pd (metal) scattering path, whereas the Ni K-edge spectrum in (**d**) was fitted by a Ni-O scattering path (i.e., Ni-O first coordination shell). Extended X-ray absorption fine structure revealed that the Pd-O and Pd-Pd bond distances for Pd/$Ni(OH)_2$ catalyst were 2.01 ± 0.02 Å and 2.72 ± 0.03 Å, respectively, with Pd-O and Pd-Pd coordination numbers of 1.2 ± 0.2 and 3.6 ± 0.7, respectively. Ni K-edge EXAFS showed Ni-O and Ni-OH bond distances in the Pd/$Ni(OH)_2$ catalyst were 1.83 ± 0.02 Å and 2.04 ± 0.02 Å, respectively, and the Ni-O and Ni-OH coordination numbers 2.1 ± 0.4 and 3.9 ± 0.8, respectively.

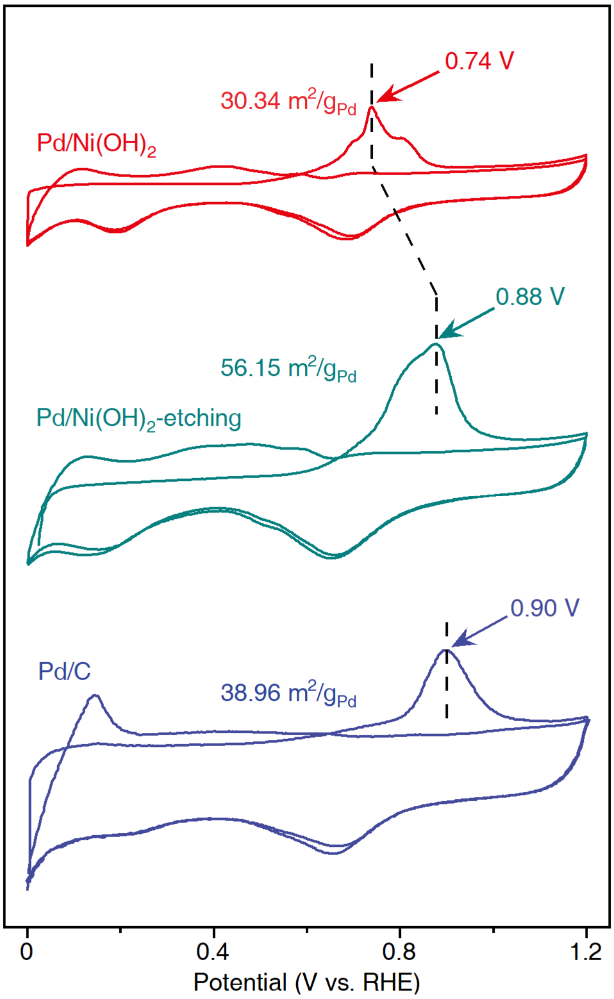

Supplementary Fig. S24 | CO stripping experiments for different catalysts. CO stripping tests were performed to evaluate the CO oxidation potential of different catalysts and to determine the specific electrochemical surface areas of the catalysts with 95% iR corrected. The CO-stripping experiments determined that $Pd/Ni(OH)_2$ required a lower potential (0.74 V versus RHE, top) to remove CO while compared with $Pd/Ni(OH)_2$-etching (0.88 V versus RHE, middle) and Pd/C (0.90 V versus RHE, bottom), suggesting the better CO tolerance of $Pd/Ni(OH)_2$ catalyst.

We calculated the electrochemical specific surface area (ECSA, m^2/g_{Pd}) based on Pd loadings measured by ICP-OES and the CO-stripping experiments. The calculations were performed as follows:

$$\mathrm{ECSA} = \frac{Q}{M \times 0.42} \qquad (6)$$

$$Q = \mathrm{I} \times \mathrm{t} = \frac{\int IdE}{v} \qquad (7)$$

where M was the mass of Pd (mg) on the electrode, 0.42 mC/cm^2 was the charge consumed during electrooxidation of a CO monolayer, Q was the total charge (C) used for CO electrooxidation, v was the scanning rate (0.02 V s^{-1}), and ∫IdE was the integrated area of the cyclic voltammetry curve of CO-stripping corrected for the double layer current.

Calculations of the ECSA of the samples were provided as follows：

The ECSA of Pd/C catalyst：

(Catalyst weight = (11.11 mg/mL × 0.30 mL)/0.5 cm^2 = 6.67 mg/cm^2, Pd loading =15.52 wt.%)

$ECSA_{catalyst} = \frac{Q}{M\times 0.42}$ = 84.65 mC /(3.33 mg × 15.52% × 0.42 mC/cm^2) = 390.09 cm^2/mg_{Pd} = 39.01 m^2/g_{Pd}

$ECSA_{electrode} = \frac{Q}{0.42}$ = 84.65 mC/0.42 mC/cm^2 = 201.91 cm^2

The ECSA of Pd/$Ni(OH)_2$ catalyst：

(Catalysts weight = (5.00 mg/mL × 0.30 mL)/0.50 cm^2 = 3.00 mg/cm^2, Pd loading =29.98 wt%)

$ECSA_{catalyst} = \frac{Q}{M\times 0.42}$ = 57.30 mC/(1.50 mg × 29.98% × 0.42 mC/cm^2) = 303.37 cm^2/mg_{Pd} = 30.34 m^2/g_{Pd}

$ECSA_{electrode} = \frac{Q}{0.42}$ = 57.30 mC/0.42 mC/cm^2 = 136.42 cm^2

The ECSA of Pd/$Ni(OH)_2$-etching catalyst:

(Catalysts weight = (5.00 mg/mL × 0.30 mL)/0.50 cm^2 = 3.00 mg/cm^2, Pd loading =32.61 wt%)

$ECSA_{catalyst} = \frac{Q}{M\times 0.42}$ = 115.35 mC/(1.50 mg × 32.61% × 0.42 mC/cm^2) = 561.46 cm^2/mg_{Pd} = 56.15 m^2/g_{Pd}

$ECSA_{electrode} = \frac{Q}{0.42}$ = 115.35 mC/0.42 mC/cm^2 = 274.64 cm^2

5

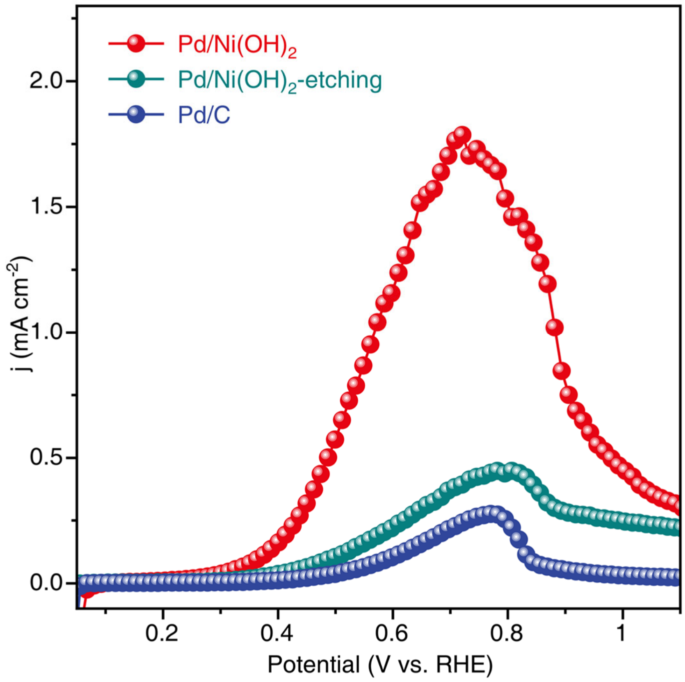

Supplementary Fig. S25 | Specific activity of different catalysts for HMFOR. Cyclic voltammetry (CV) curves were normalized by ECSA to evaluate specific activity for HMFOR.

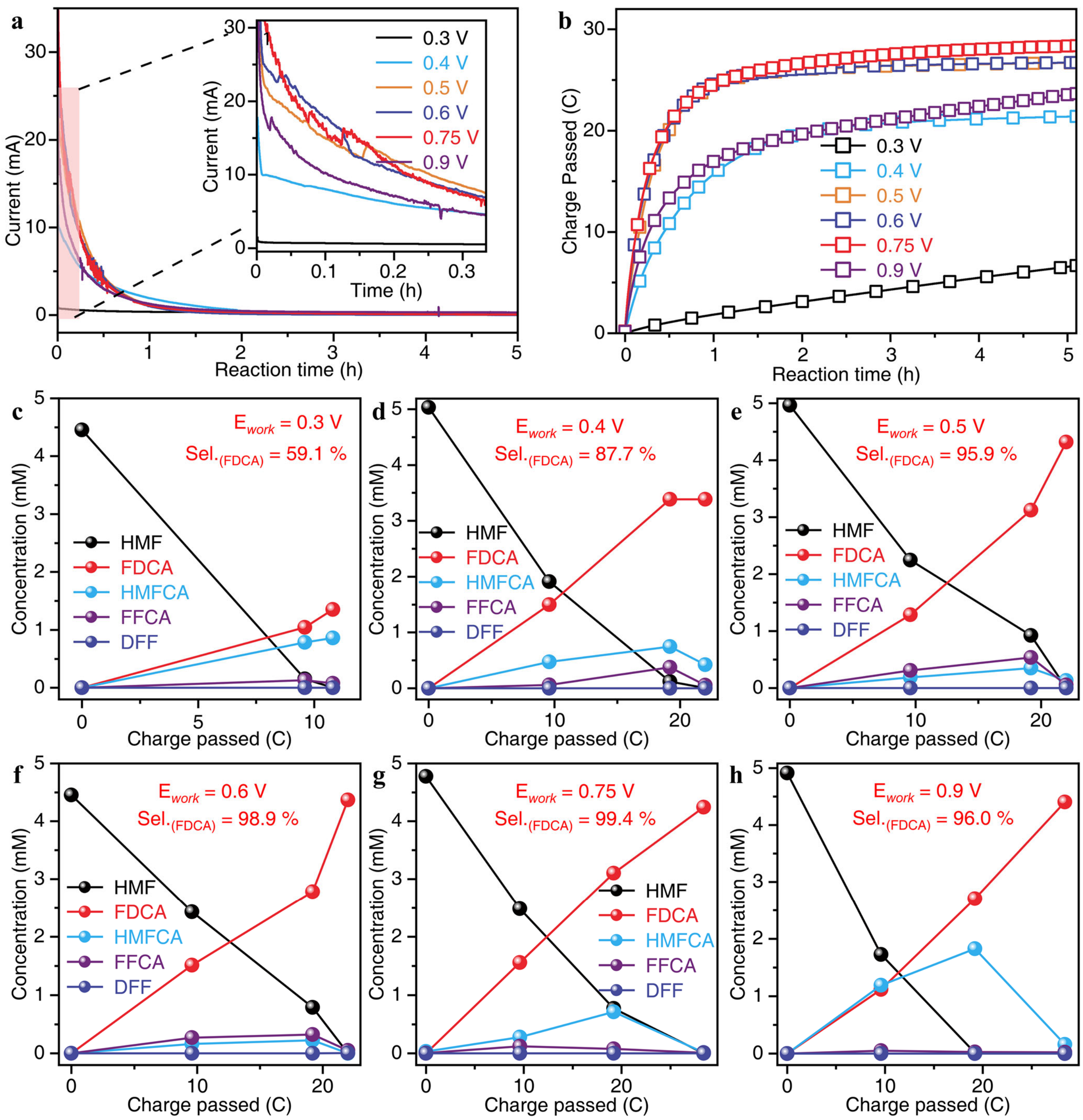

Supplementary Fig. S26 | HMFOR performance of Pd/Ni(OH)$_2$ catalyst under different potentials. a, Plots of current (I) versus reaction time (t). **b,** Plots of charge passed (Q) versus reaction time (t) for Pd/Ni(OH)$_2$ at the different potentials in Ar-saturated 1 M KOH + 5 mM HMF solution. **c-h,** Concentration versus charge passed plots for HMF, FDCA, and the intermediates such as HMFCA, DFF, and FFCA for Pd/Ni(OH)$_2$ at the different potentials of (**c**) 0.3 V, (**d**) 0.4 V, (**e**) 0.5 V, (**f**) 0.6 V, (**g**) 0.75 V, (**h**) 0.9 V.

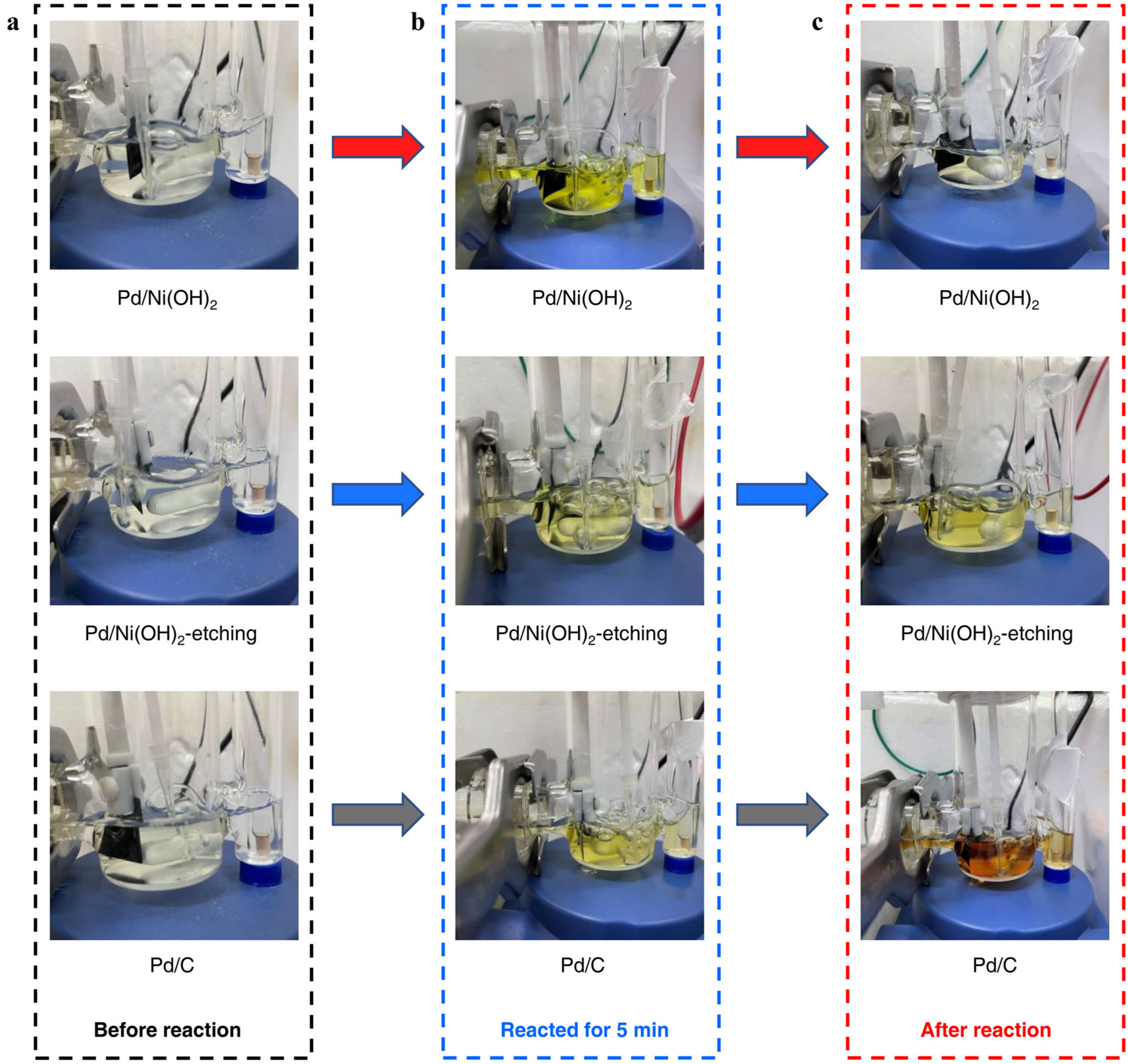

Supplementary Fig. S27 | Photos of the electrolyte in the anode cell during the HMFOR with different catalysts. a, Photos of 1.0 M KOH +5 mM HMF electrolyte containing $Pd/Ni(OH)_2$, $Pd/Ni(OH)_2$-etching, and Pd/C electrodes before the HMFOR. **b,** Photos of the electrolyte after working at 0.75 V versus RHE for 5 min. **c,** Photos of the electrolyte working at 0.75 V versus RHE at the end of the reaction. As shown in the photos above, the electrolytes of $Pd/Ni(OH)_2$-etching and Pd/C were orange and dark orange, respectively, from humins (the self-condensation products of HMF). On the contrary, the electrolyte of $Pd/Ni(OH)_2$ was almost colorless, indicating that the side reaction of HMF self-condensation was blocked and leading to high selectivity of HMFOR and FDCA yield. The fast reaction rate of HMFOR over the $Pd/Ni(OH)_2$ catalyst overcame the reaction of HMF self-condensation.

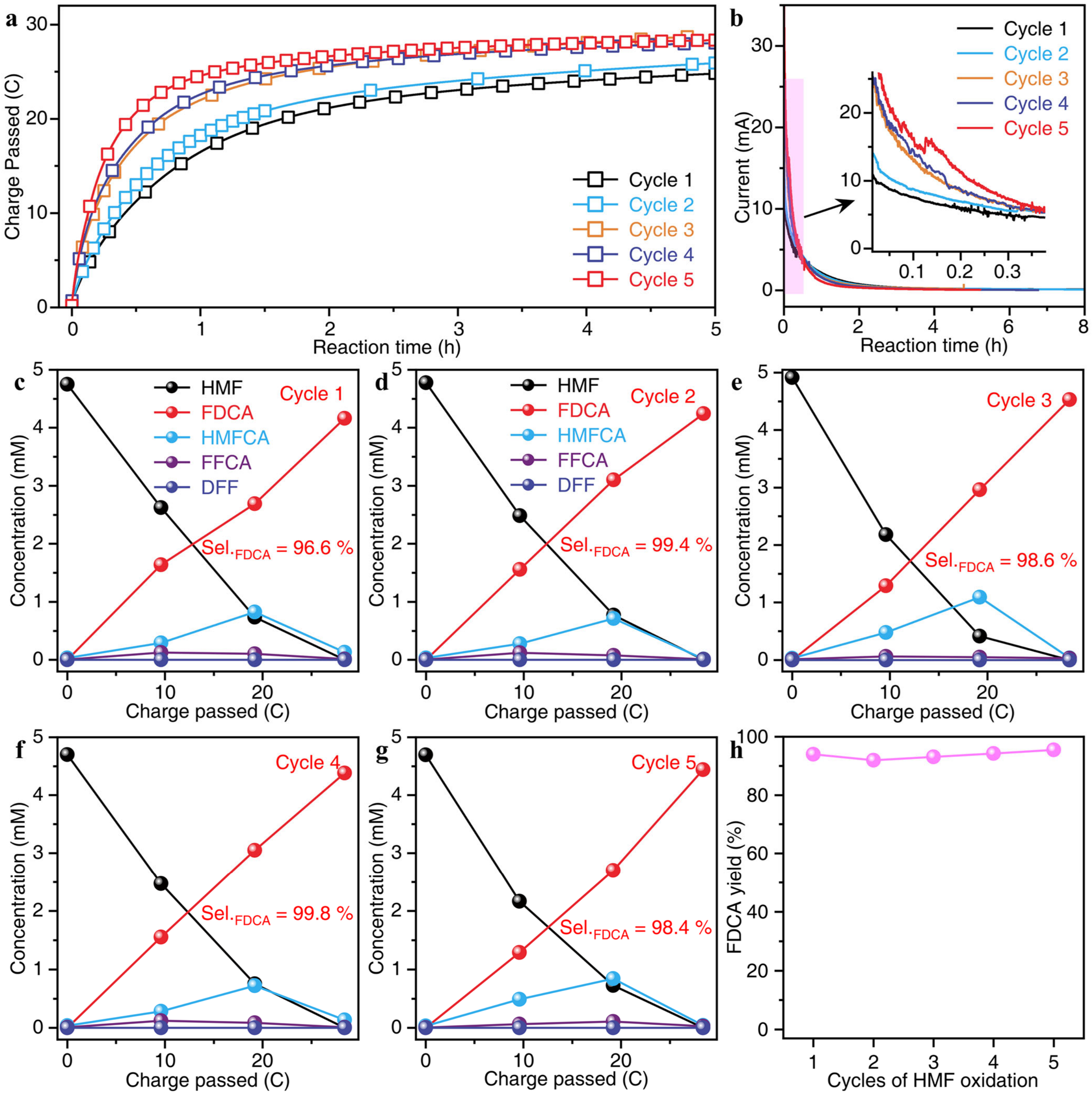

Supplementary Fig. S28 | Cycle stability of Pd/Ni(OH)$_2$ for HMFOR at 0.75 V versus RHE. (**A**) The charge passed to Pd/Ni(OH)$_2$ during HMFOR at 0.75 V versus RHE over 5 continuous cycles in an Ar-saturated 1 M KOH + 5 mM HMF solution. **b,** Chronoamperometry curves measured at 0.75 V versus RHE in Ar-saturated 1 M KOH + 5 mM HMF solution for 5 cycles. **c-g,** Plots of concentration versus charge passed for HMF, FDCA, HMFCA, DFF, and FFCA measured after (**c**) Cycle 1, (**d**) Cycle 2, (**e**) Cycle 3, (**f**) Cycle 4, (**g**) Cycle 5. **h,** Yield of FDCA over 5 continuous cycles. The FDCA yield of each cycle test is more than 90%, illustrating the great cycle stability of the Pd/Ni(OH)$_2$ catalyst for HMFOR in 1M KOH solution.

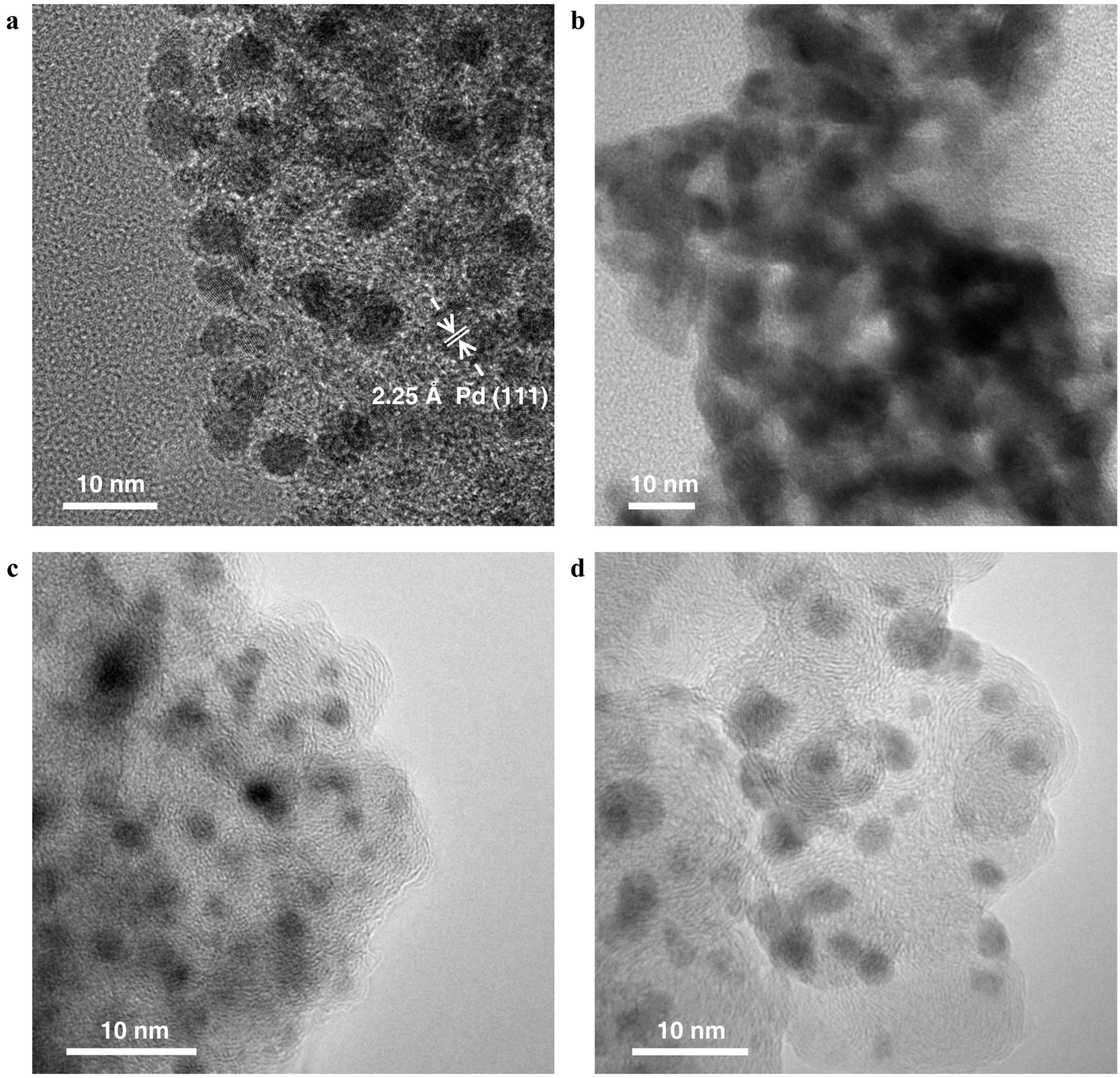

Supplementary Fig. S29 | Representative TEM images of Pd/C and Pd/Ni(OH)$_2$ catalysts after HMFOR. Representative TEM images of Pd/C **a,** before and **b,** after reaction at 0.75 V versus RHE for 3 cycles of chronoamperometry tests. TEM images of Pd/Ni(OH)$_2$ **c,** before and **d,** after reaction at 0.75 V versus RHE for 5 cycles of chronoamperometry tests. As shown in TEM images of **a** and **b**, Pd/C was heavily aggregated after HMFOR. In contrast, the Pd/Ni(OH)$_2$ catalyst did not change significantly, indicating that the Ni^{2+}-O-Pd interfaces prevented the agglomeration of Pd nanoparticles during the reaction, greatly improving the cycle stability of the catalyst.

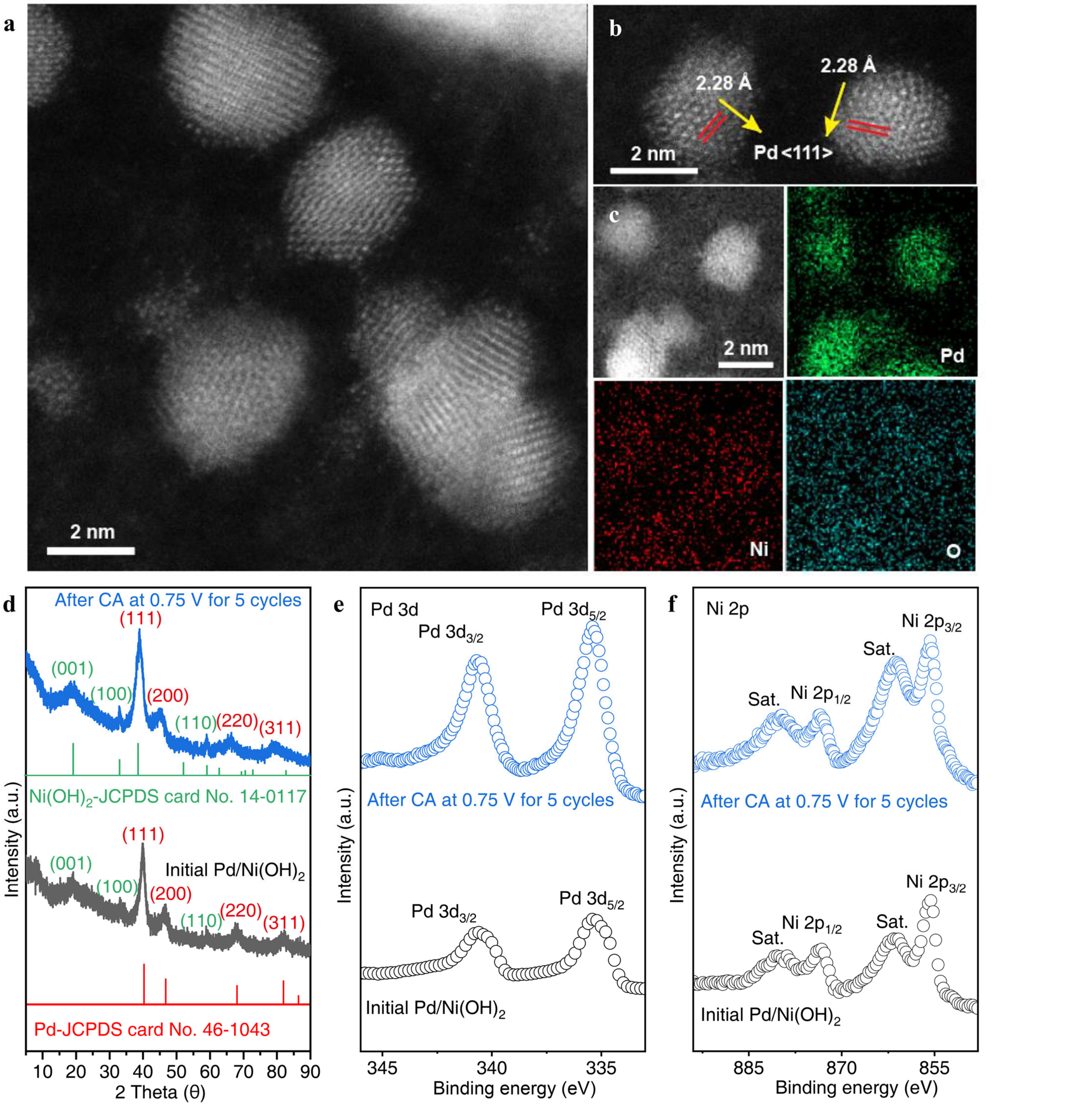

Supplementary Fig. S30 | Structural characterization of the Pd/Ni(OH)$_2$ catalyst after 5 cycles of chronoamperometry test at 0.75 V versus RHE. a, b, Representative probe-corrected STEM image of Pd/Ni(OH)$_2$ catalyst after 5 cycles. **c,** Probe-corrected STEM image and corresponding EDS mapping of Pd/Ni(OH)$_2$ catalyst after 5 cycles test. **d,** X-ray diffraction patterns for Pd/Ni(OH)$_2$ before and after 5 cycles test. **e**,**f**, XPS spectra of Pd 3d (**e**) and Ni 2p (**f**) for Pd/Ni(OH)$_2$ catalyst before and after 5 cycles of chronoamperometry test at 0.75 V versus RHE. There were no significant changes in the size, dispersion, and valence state of Pd and Ni in the Pd/Ni(OH)$_2$ catalyst after the cycle stability test.

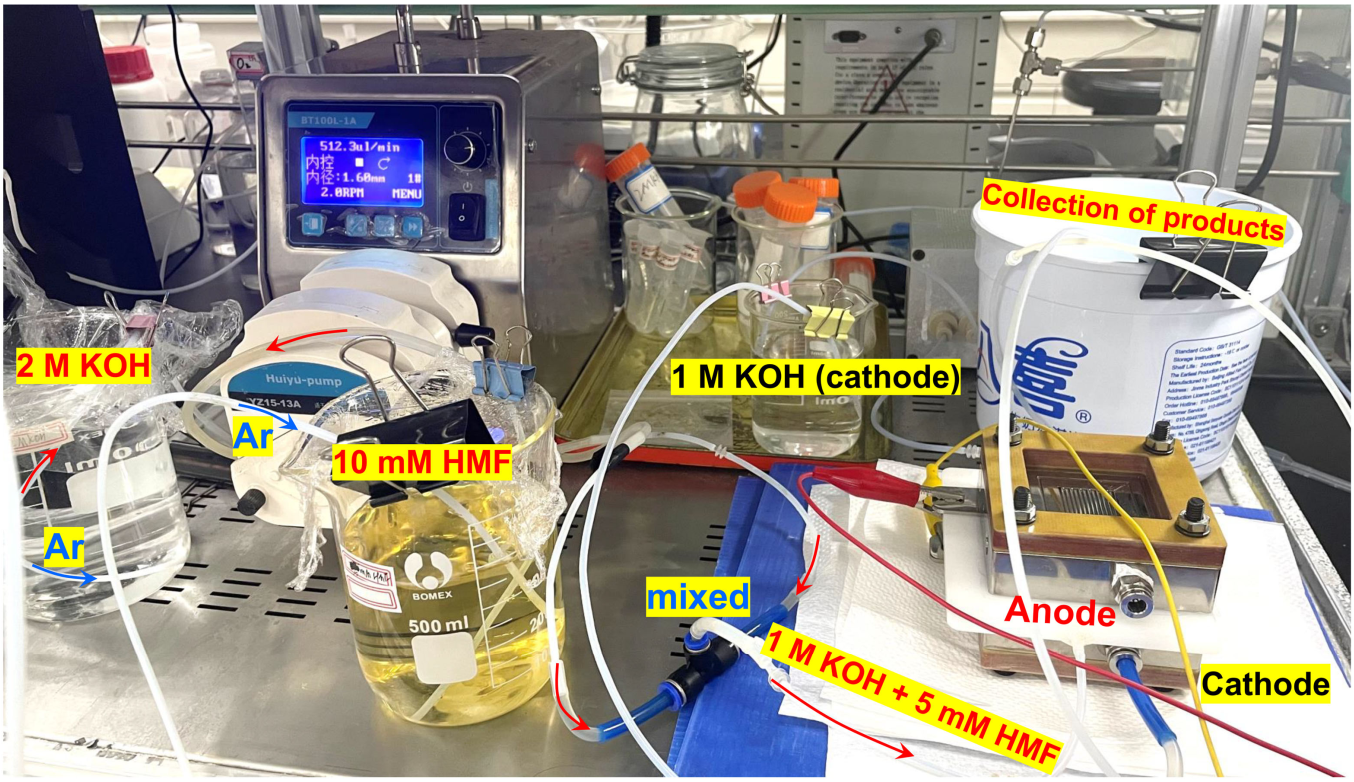

Supplementary Fig. S31 | The photo of the two-electrode flow cell reactor.

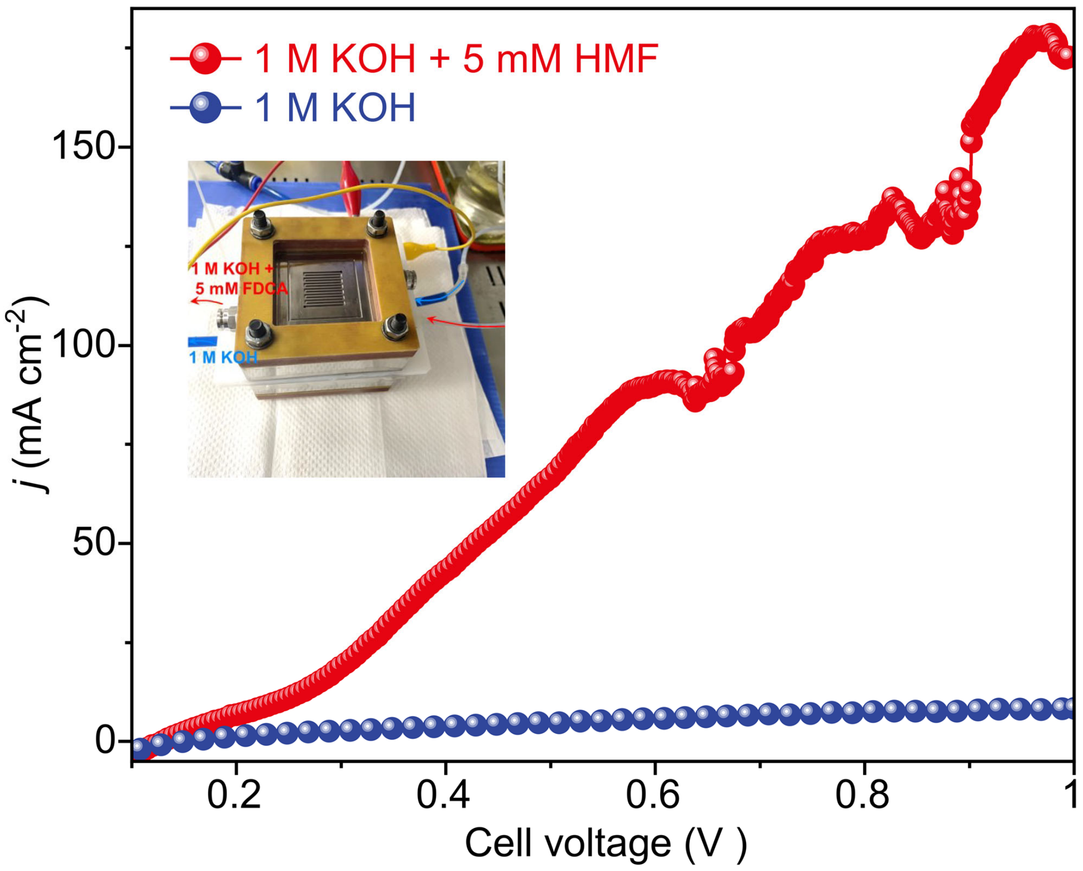

Supplementary Fig. S32 | Linear sweep voltammetry measurement in the two-electrode flow cell. The $Pd/Ni(OH)_2$ catalyst showed good performance for the electrooxidation of 5 mM HMF in the flow cell reactor with low cell voltages (< 1.0 V), the LSV curves were 95% iR corrected, and double-layer capacity deducted.

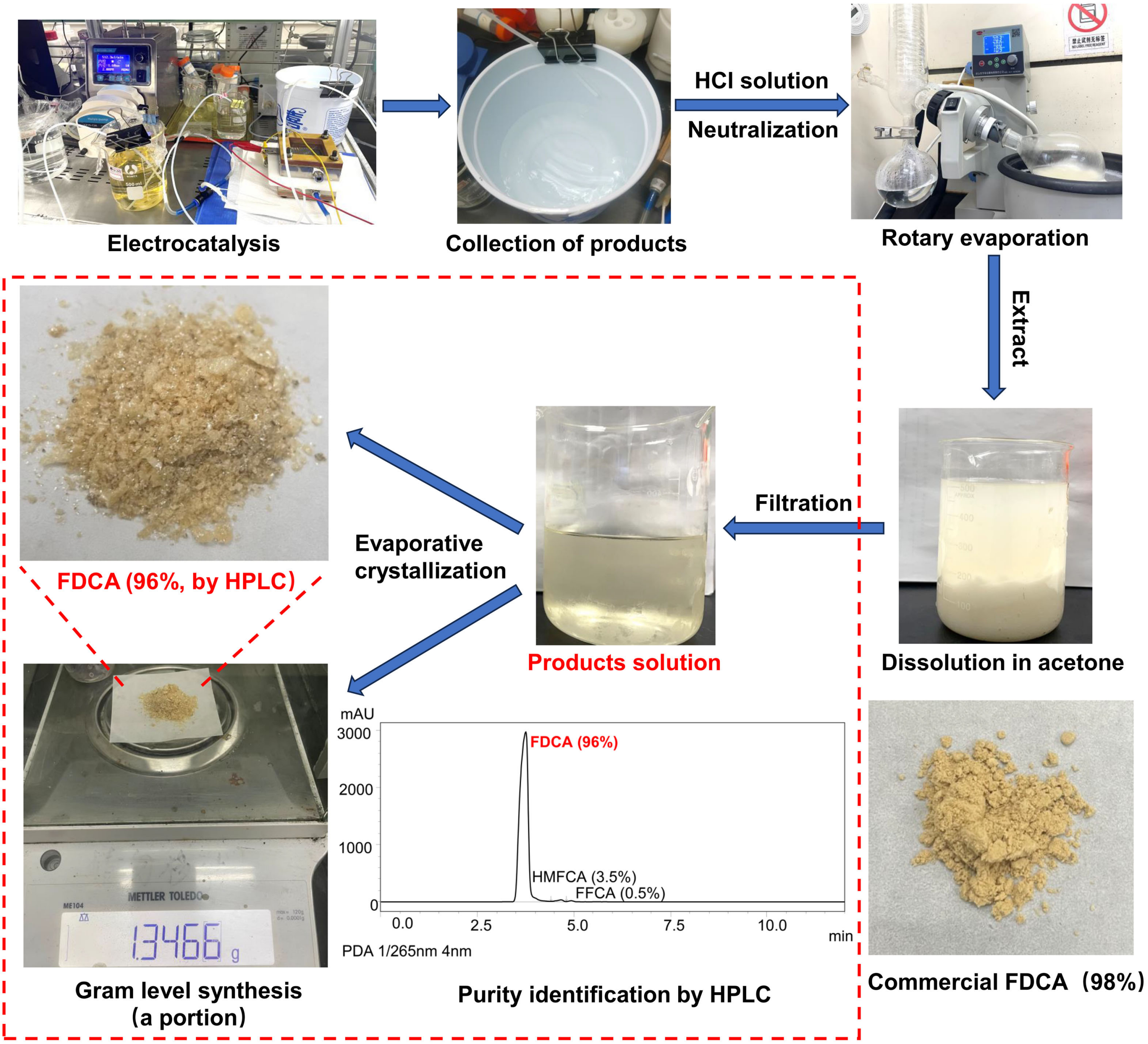

Supplementary Fig. S33 | The photographs of the electrocatalytic upgrading process of HMF to FDCA. The electrocatalytic upgrading process of HMF to FDCA was conducted in a two-electrode flow cell reactor. To avoid HMF self-condensation in alkaline conditions, 10 mM HMF aqueous solution was mixed with 2.0 M KOH with a volume ratio of 1:1 before pumping into the anode chamber with a flow rate of 1 mL/min. The voltage of the flow cell reactor was fixed at 0.85 V to drive both the cathodic HER and anodic HMFOR reactions. The electrolyte pumping out from the anode was collected and then neutralized to pH=7 by adding hydrochloric acid. The water in the electrolyte was removed by rotary evaporation at 70 °C, leading to a mixture of KCl and FDCA. The product of FDCA was simply extracted from the mixture by acetone. The purity of the obtained FDCA was analyzed by HPLC (up to 96% selectivity).

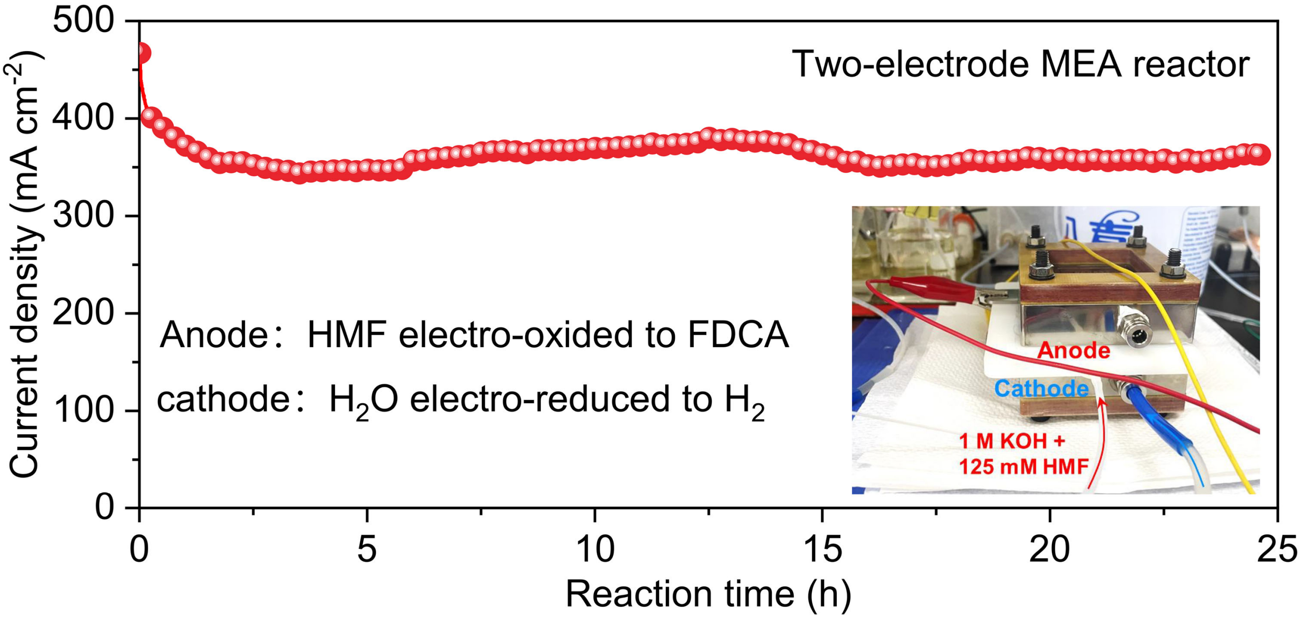

Supplementary Fig. S34 | Stability test of Pd/$Ni(OH)_2$ catalyst in the two-electrode flow cell reactor under a high current density. Reaction conditions: Commercial 20 wt% Pt/C (10.0 mg) sprayed on carbon paper (1.5×1.5 cm^2, 4.5 mg/cm^2) was employed as the cathode. 1.0 M KOH electrolyte was circulated through the cathode chamber by a peristaltic pump with a flow rate of 40 mL/min. 20.0 mg Pd/$Ni(OH)_2$ catalyst loaded on a 0.25 cm^2 (0.5×0.5 cm^2) carbon felt was used as the anode. The flow cell voltage was fixed at 1.05 V with 95% iR corrected to drive the cathodic HER and anodic HMFOR. The anodic electrolyte was 1.0 M KOH + 125 mM HMF with a flow rate of 2.5 mL/min.

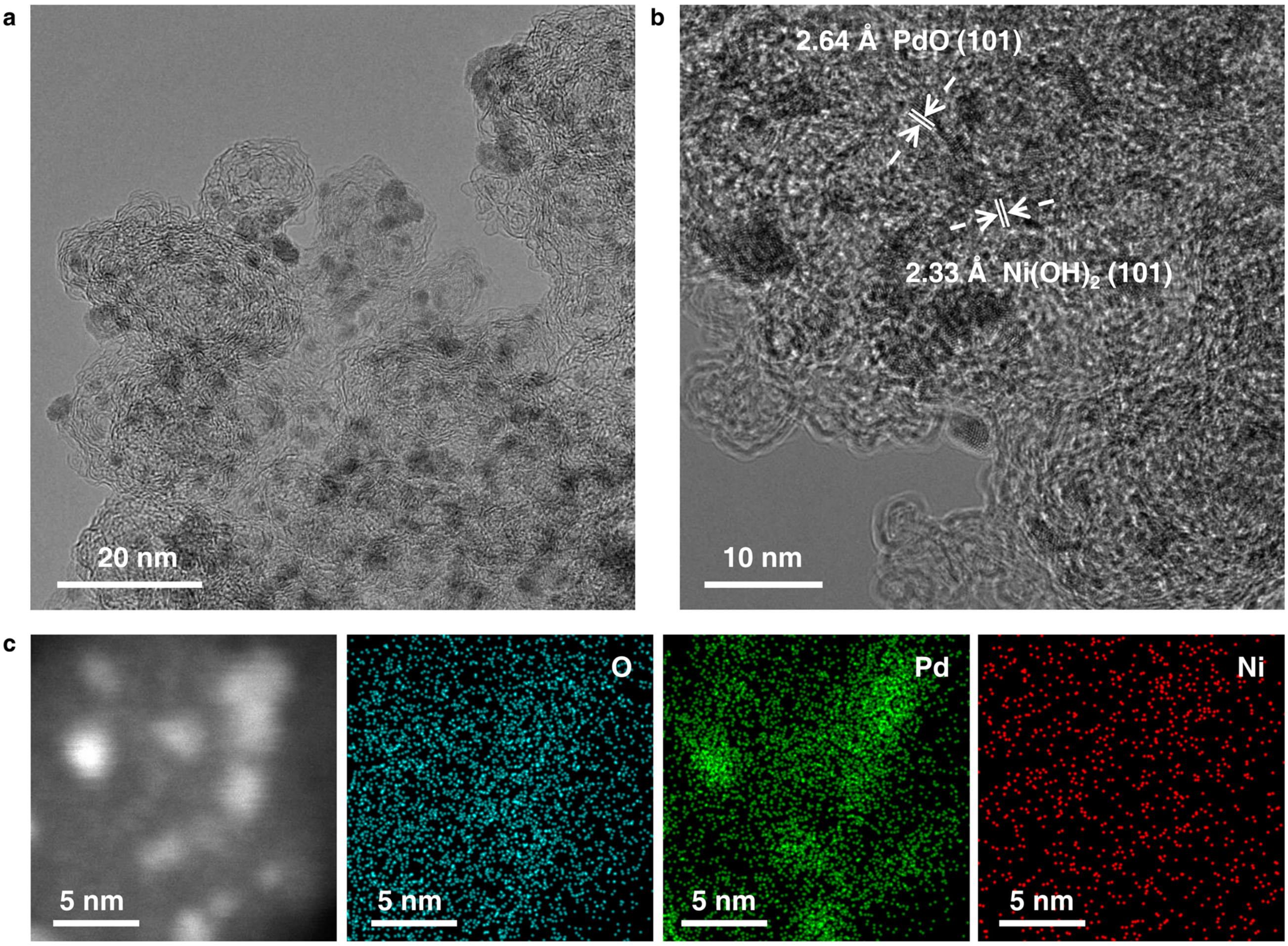

Supplementary Fig. S35 | Structural characterizations of $Pd^{2+}/Ni(OH)_2$ catalyst. a, Representative TEM image. **b,** HRTEM image of the $Pd^{2+}/Ni(OH)_2$ catalyst. **c,** STEM image and corresponding EDS mapping of the $Pd^{2+}/Ni(OH)_2$ catalyst.

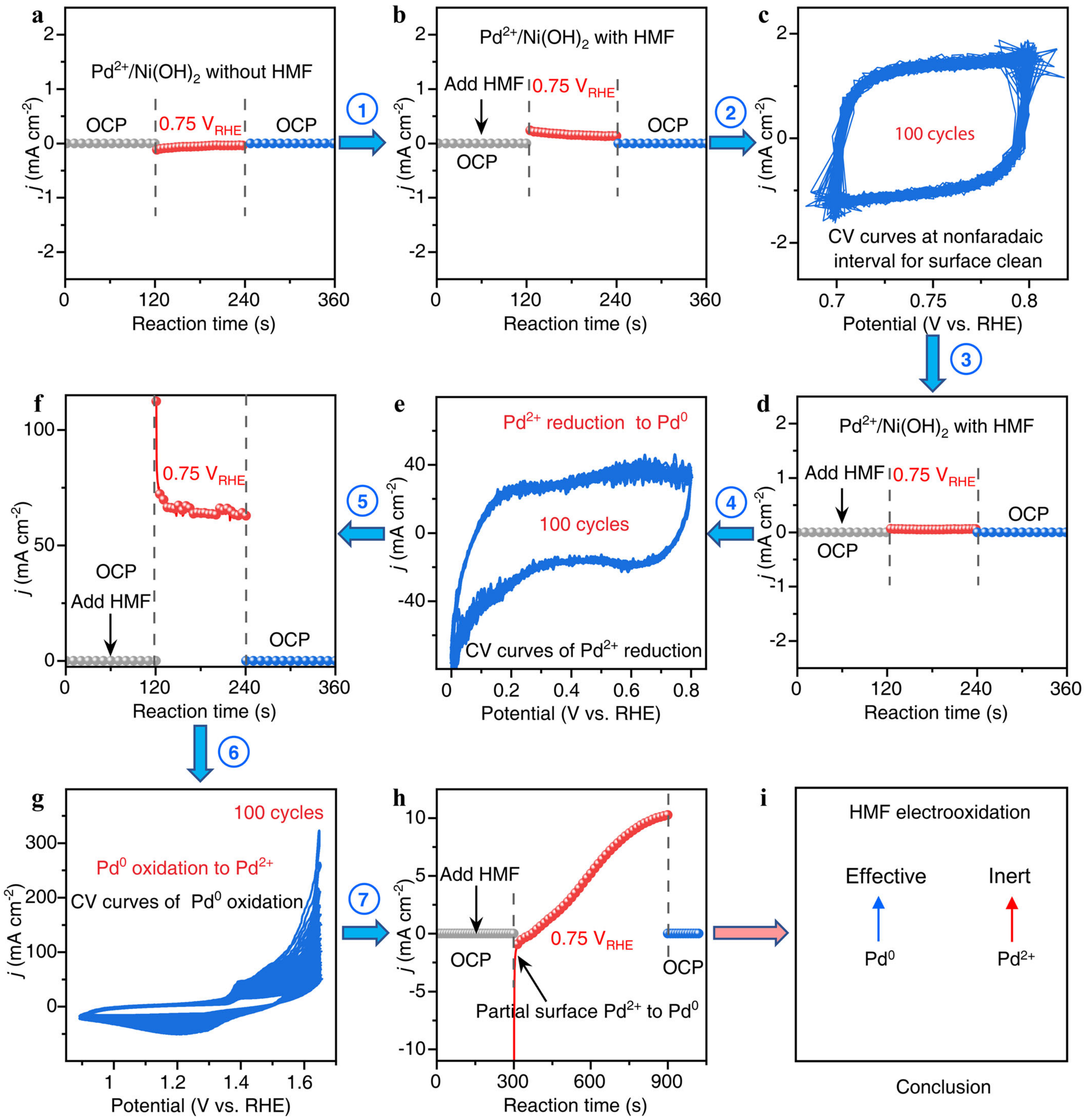

Supplementary Fig. S36 | The valence state of Pd for HMFOR. a,**b**, The potential step chronoamperometry experiments on the $Pd^{2+}/Ni(OH)_2$ catalyst without (**a**) and with (**b**) HMF in the electrolytes, respectively. **c**, The CV measurement on $Pd^{2+}/Ni(OH)_2$ catalyst with voltage between 0.7 ~ 0.8 V *vs*. RHE). **d**, The potential step chronoamperometry experiment on the $Pd^{2+}/Ni(OH)_2$ catalyst after CV measurement (**c**) with HMF in the electrolyte. **e**, The CV measurement on $Pd^{2+}/Ni(OH)_2$ catalyst with voltage between 0.0 ~ 0.8 V *vs*. RHE). After this CV cycle, the $Pd^{2+}/Ni(OH)_2$ catalyst was reduced into a $Pd/Ni(OH)_2$ catalyst. **f**, The potential step chronoamperometry experiment on the $Pd^{2+}/Ni(OH)_2$ catalyst after CV measurement (**e**) with HMF in the electrolyte. **g**, The CV measurement on $Pd/Ni(OH)_2$ catalyst with voltage between 0.8 ~ 1.7 V *vs*. RHE). After this CV cycle, the $Pd/Ni(OH)_2$ catalyst was oxidized into a $Pd^{2+}/Ni(OH)_2$ catalyst again. **h**, The potential step chronoamperometry experiment on the $Pd/Ni(OH)_2$ catalyst after CV measurement (**g**) with HMF in the electrolyte. **i**, The demonstration of the active Pd oxidation state for HMFOR. The experiments above were performed to determine which Pd valence state(s) were active sites in $Pd/Ni(OH)_2$ for low-potential HMF oxidation. To investigate the valence state of Pd for efficient HMFOR, $Pd^{2+}/Ni(OH)_2$ without electrochemical reduction was directly used for HMF electrooxidation. $Pd^{2+}/Ni(OH)_2$ was inert for HMFOR until the Pd^{2+} was electro-reduced to Pd^0. Re-oxidation of Pd^0 back to Pd^{2+} made the catalyst inert again, implying that the active sites for HMFOR at low potentials (< 1.0 V versus RHE) were Pd^0 sites.

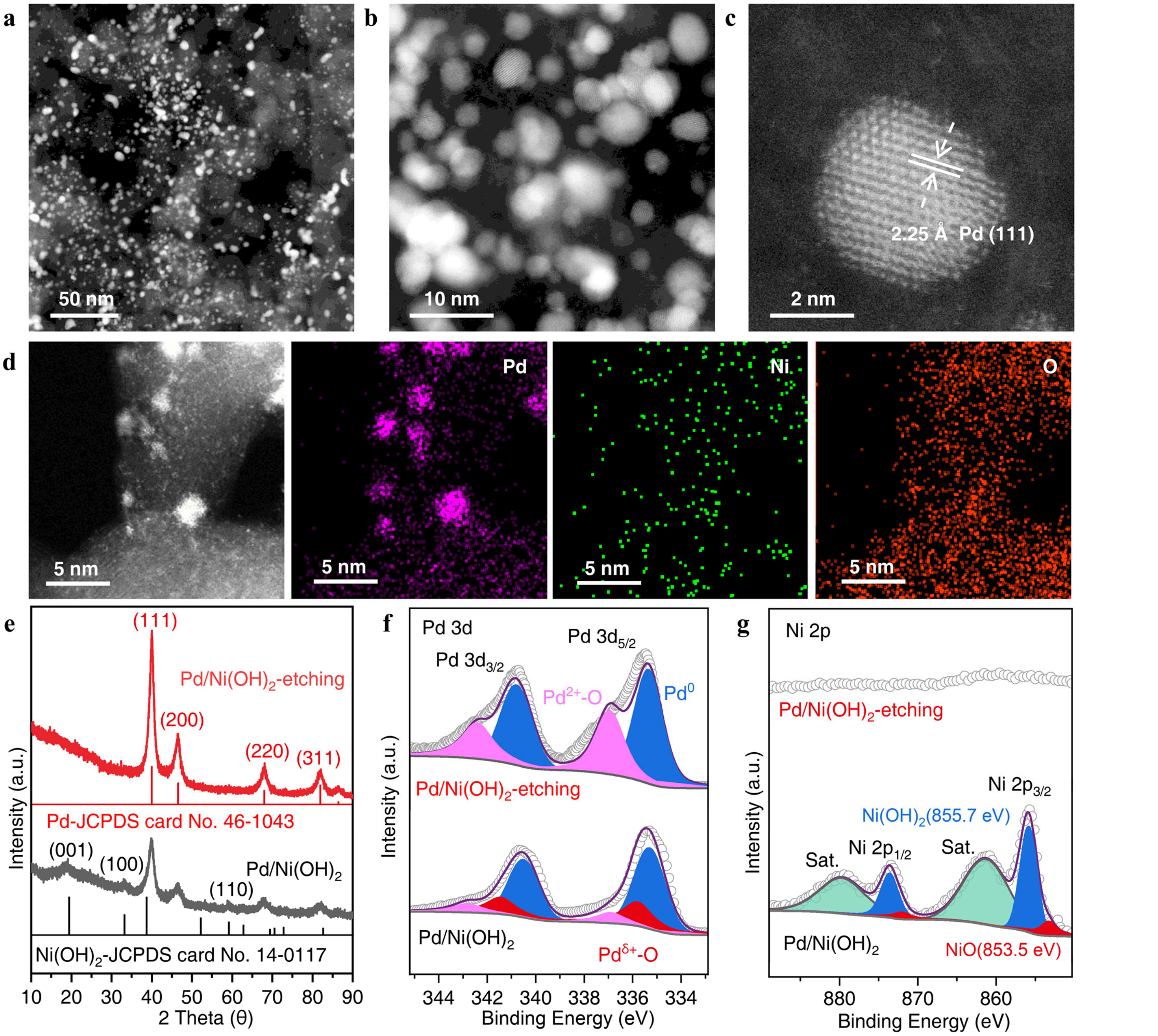

Supplementary Fig. S37 | Structural characterizations of Pd/Ni(OH)2-etching catalyst. a-c, Representative Probe-corrected STEM images of Pd/Ni(OH)2-etching in different scales. **d,** The STEM-EDS mapping of Pd/Ni(OH)2-etching. **e,** Comparison of the X-ray diffraction patterns of Pd/Ni(OH)2-etching and Pd/Ni(OH)2. **f, g,** XPS spectra of Pd 3d and Ni 2p for Pd/Ni(OH)2-etching and Pd/Ni(OH)2, respectively. The XRD patterns and STEM images of Pd/Ni(OH)2-etching showed the absence of Ni(OH)2, but the Pd NPs remained. EDS mapping and Ni 2p XPS spectra for Pd/Ni(OH)2-etching confirmed that Ni(OH)2 was largely eliminated by the acid etching.

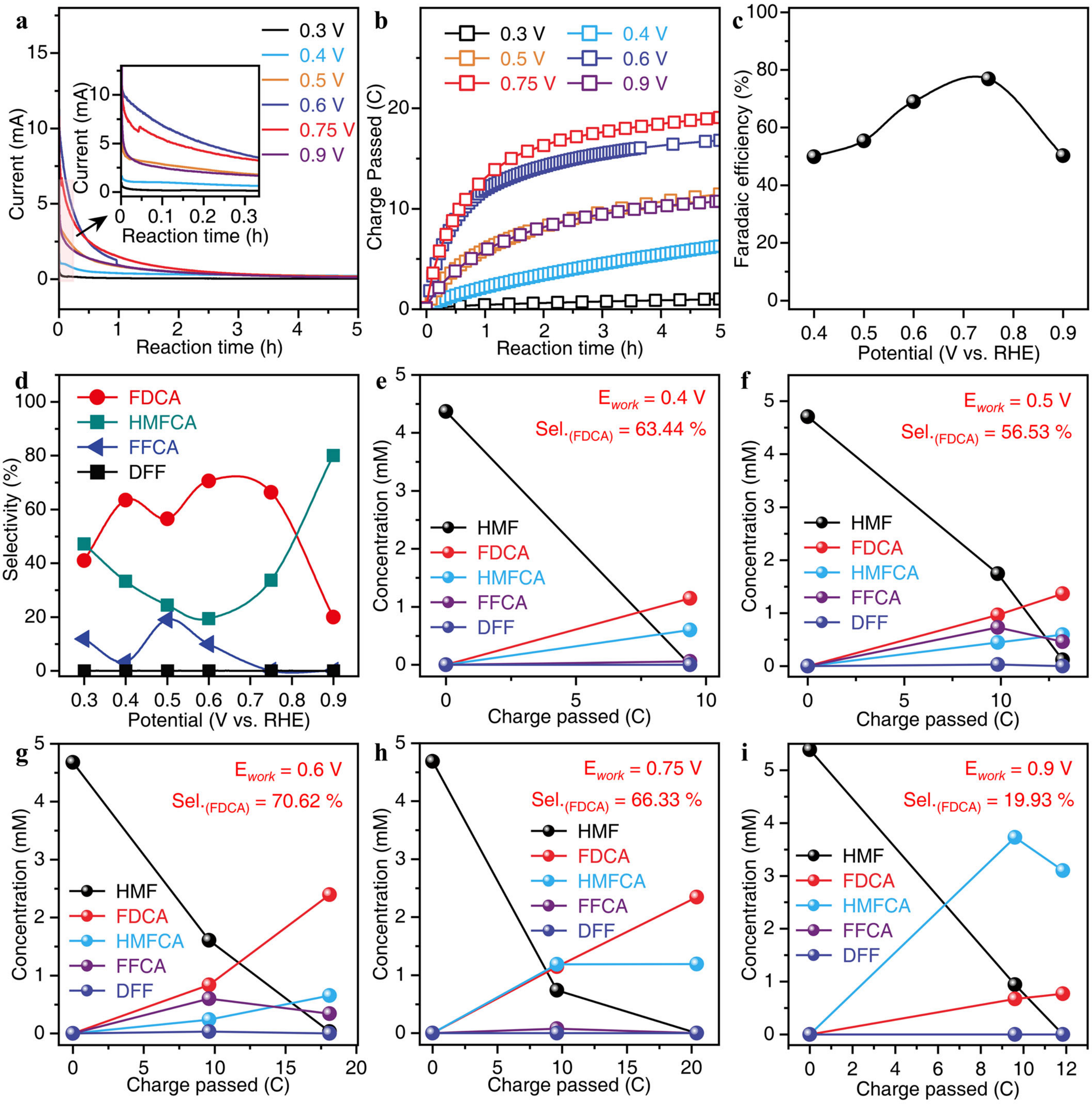

Supplementary Fig. S38 | HMFOR performance of $Pd/Ni(OH)_2$-etching at different potentials. a, The curves of current (I) versus reaction time (t) with 95% iR corrected. **b,** The curves of charge passed (Q) versus reaction time (t). **c,** The Faraday efficiency of HMFOR at each working potential. **d,** The product selectivity of $Pd/Ni(OH)_2$-etching in HMFOR measured at different potentials. **e-i,** Plots of concentration versus charge passed for HMF, FDCA, HMFCA, FFCA, and DFF during HMFOR on $Pd/Ni(OH)_2$-etching catalyst with the different potentials of (**e**) 0.4 V, (**f**) 0.5 V, (**g**) 0.6 V, (**h**) 0.75 V, (**i**) 0.9 V versus RHE.

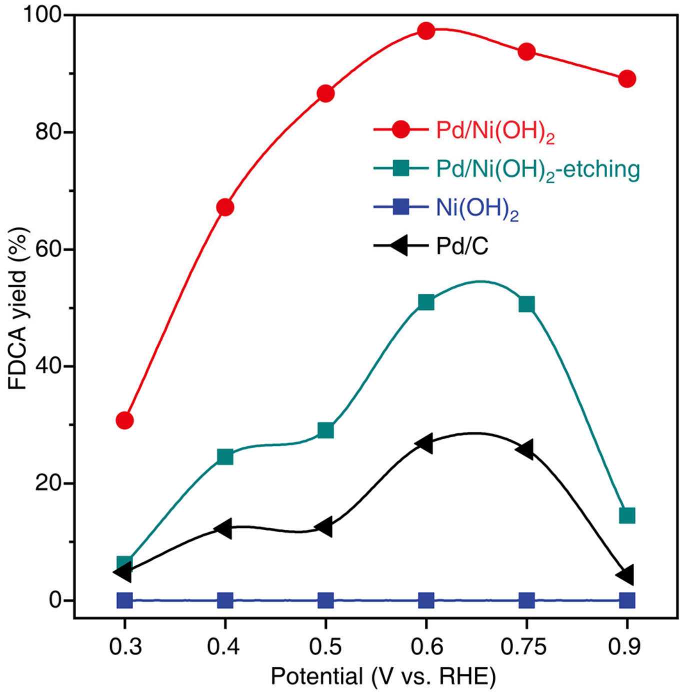

Supplementary Fig. S39 | FDCA yield versus different catalysts and potentials. The FDCA yield of $Pd/Ni(OH)_2$, $Pd/Ni(OH)_2$-etching, and Pd/C was measured at different potentials. With the Ni^{2+}-O-Pd interfaces, the $Pd/Ni(OH)_2$ catalyst demonstrated superior performance for FDCA production over $Pd/Ni(OH)_2$-etching and Pd/C catalysts.

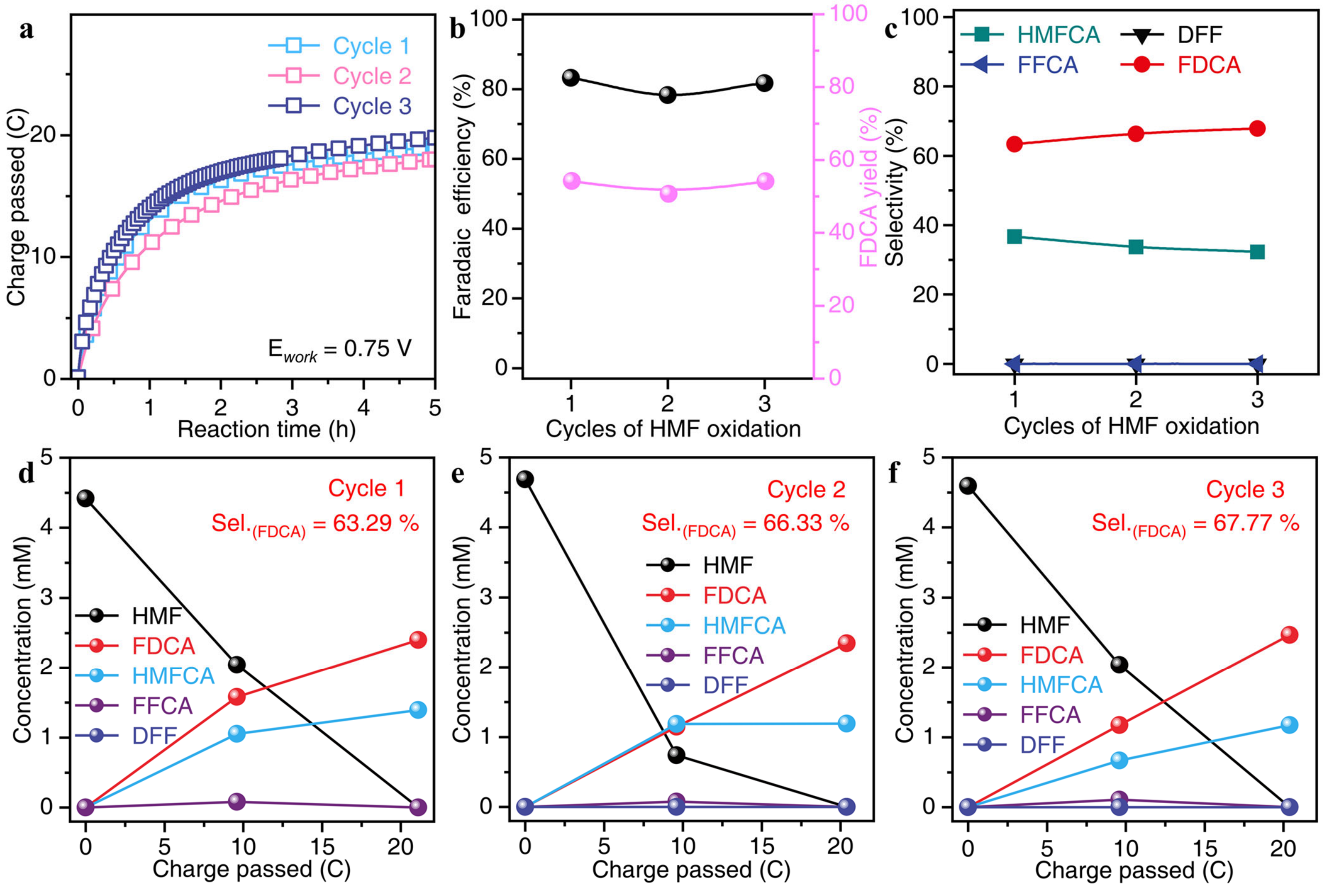

Supplementary Fig. S40 | Cycle stability of Pd/Ni(OH)$_2$-etching for HMFOR at 0.75 V versus RHE. **a,** The curves of charge passed (Q) versus reaction time (t) at each cycle. **b,** The Faraday efficiency and FDCA yield of HMFOR at each cycle. **c,** The product selectivity of Pd/Ni(OH)$_2$-etching in HMFOR measured at each cycle. **d-f,** Plots of concentration versus charge passed for HMF, FDCA, HMFCA, FFCA, and DFF during each cycle of HMFOR on Pd/Ni(OH)$_2$-etching catalyst at 0.75 V versus RHE: (**d**) Cycle 1, (**e**) Cycle 2 and (**f**) Cycle 3.

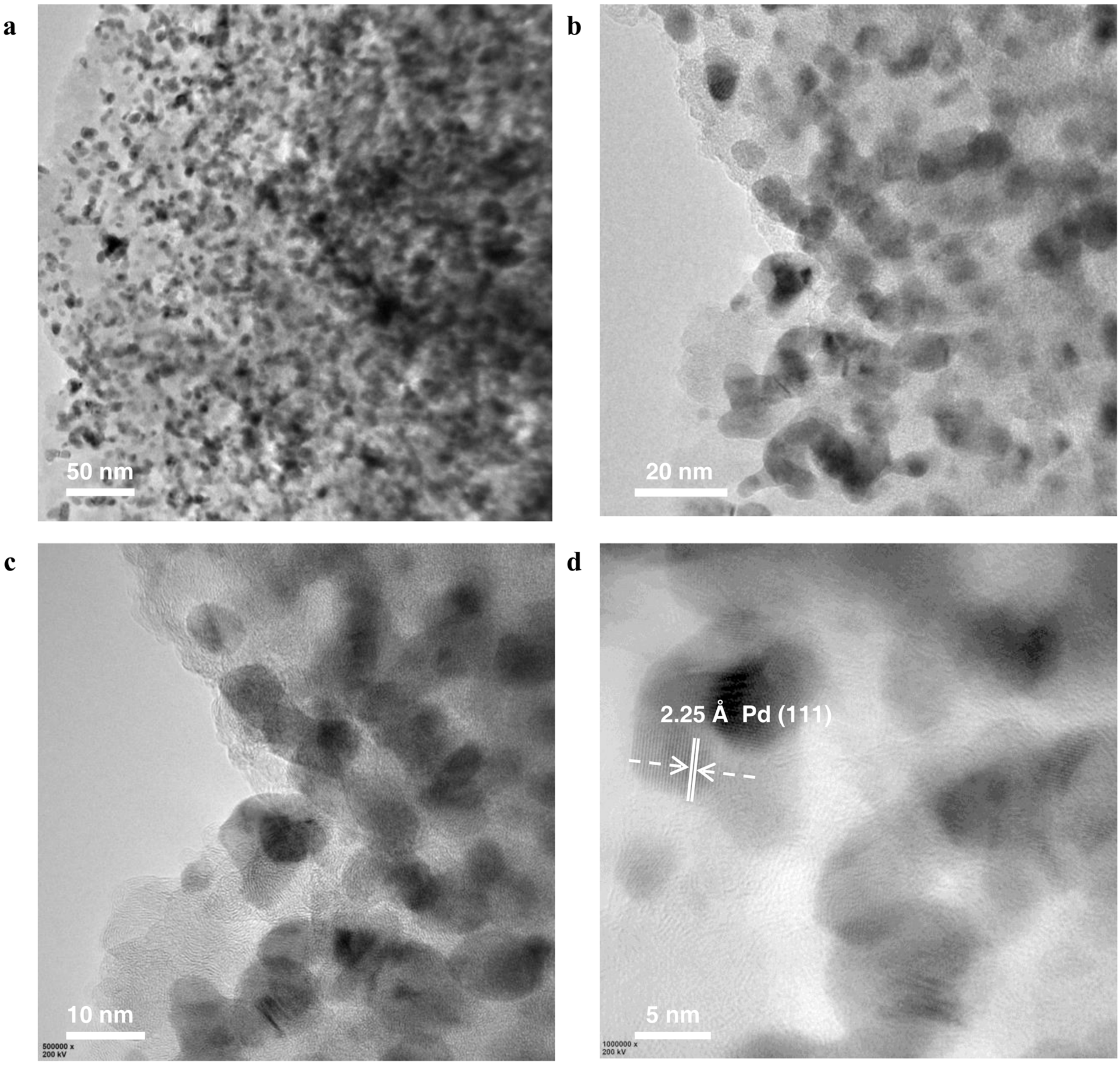

Supplementary Fig. S41 | TEM and HRTEM images of the Pd/Ni(OH)$_2$-etching catalyst after cycle stability test. a, b, Large scale TEM images of Pd/Ni(OH)$_2$-etching after reaction at 0.75 V versus RHE for the 3 cycles. **c, d,** HRTEM images of Pd/Ni(OH)$_2$-etching after the reaction. As shown in the above TEM images, the structural stability of Pd/Ni(OH)$_2$ in HMFOR decreased after acid leaching, as the obvious aggregation of Pd NPs was found after the reaction. The Ni^{2+}-O-Pd interfaces (and strong associated metal-support interaction) prevented agglomeration of Pd nanoparticles during HMFOR, thereby giving the catalyst excellent stability.

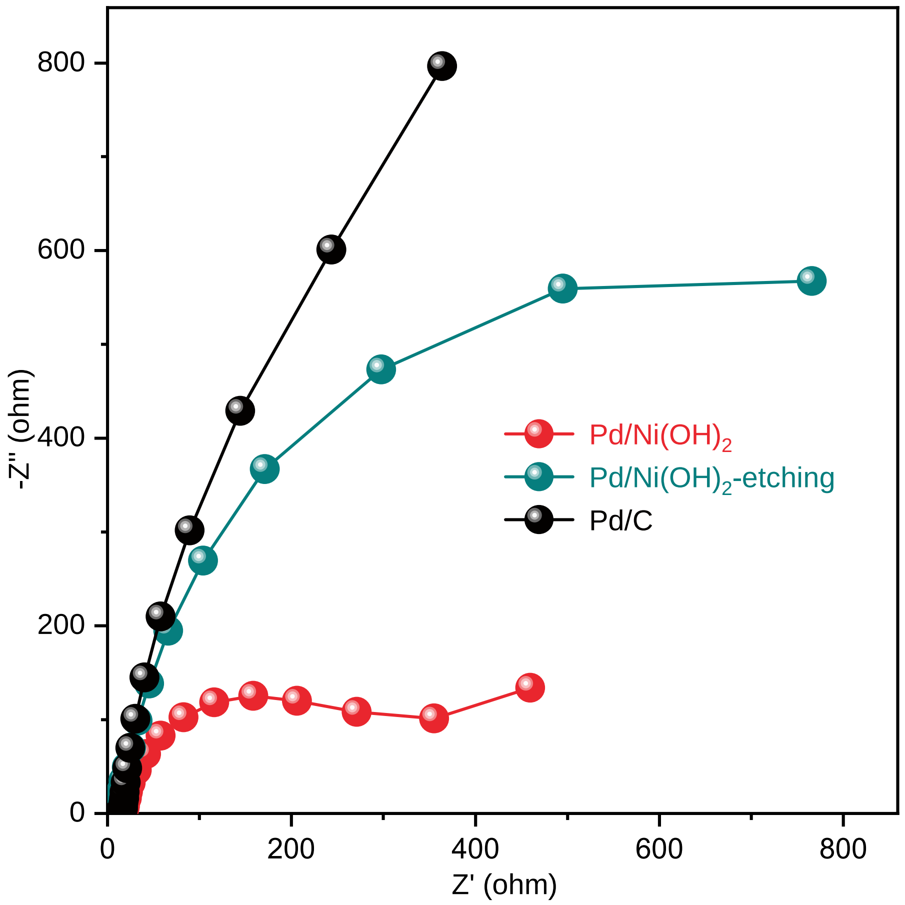

Supplementary Fig. S42 | Electrochemical impedance spectroscopy (EIS) study for different catalysts under the conditions of HMFOR.

Electrochemical impedance spectroscopy (EIS) measurements demonstrated that the $Pd/Ni(OH)_2$ catalyst showed a much smaller charge transfer resistance (R_{ct}) for HMFOR than $Pd/Ni(OH)_2$-etching and Pd/C, firmly establishing that Ni^{2+}-O-Pd interfaces were the active sites for HMFOR.

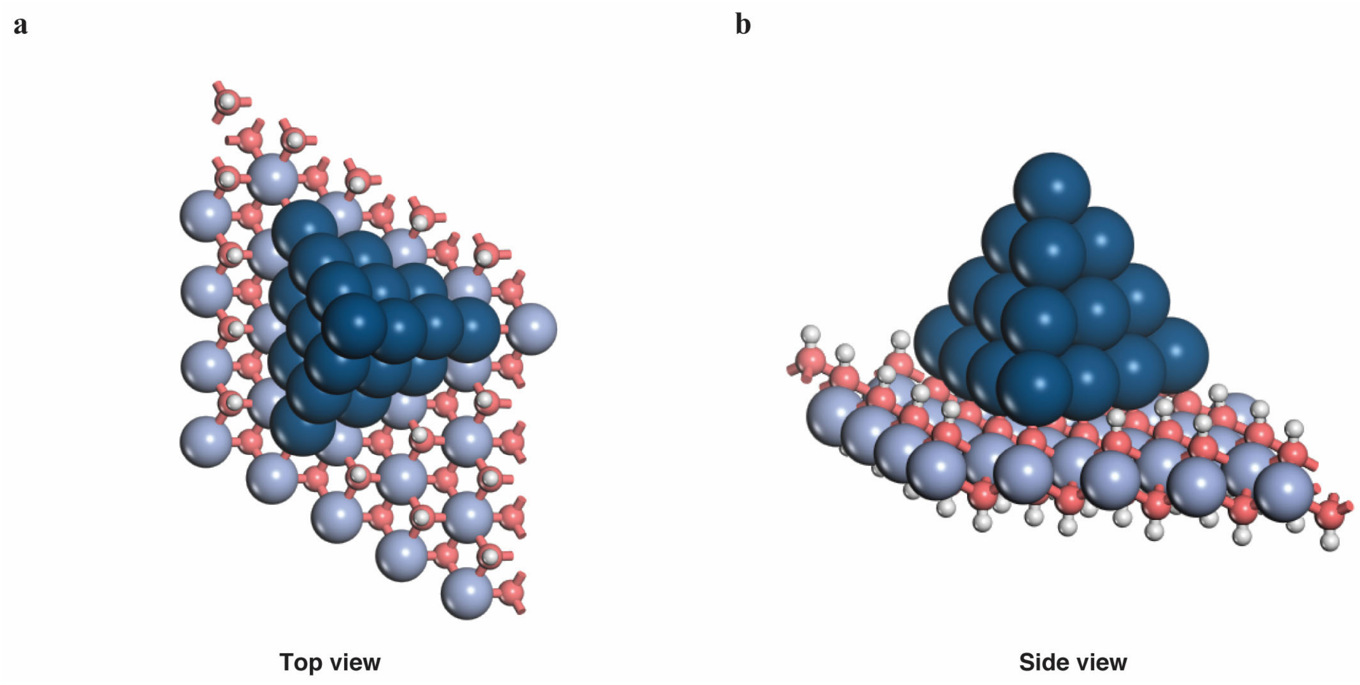

Supplementary Fig. S43 | The models of the Pd/Ni(OH)$_2$ catalyst. a, Top view. **b,** side view.

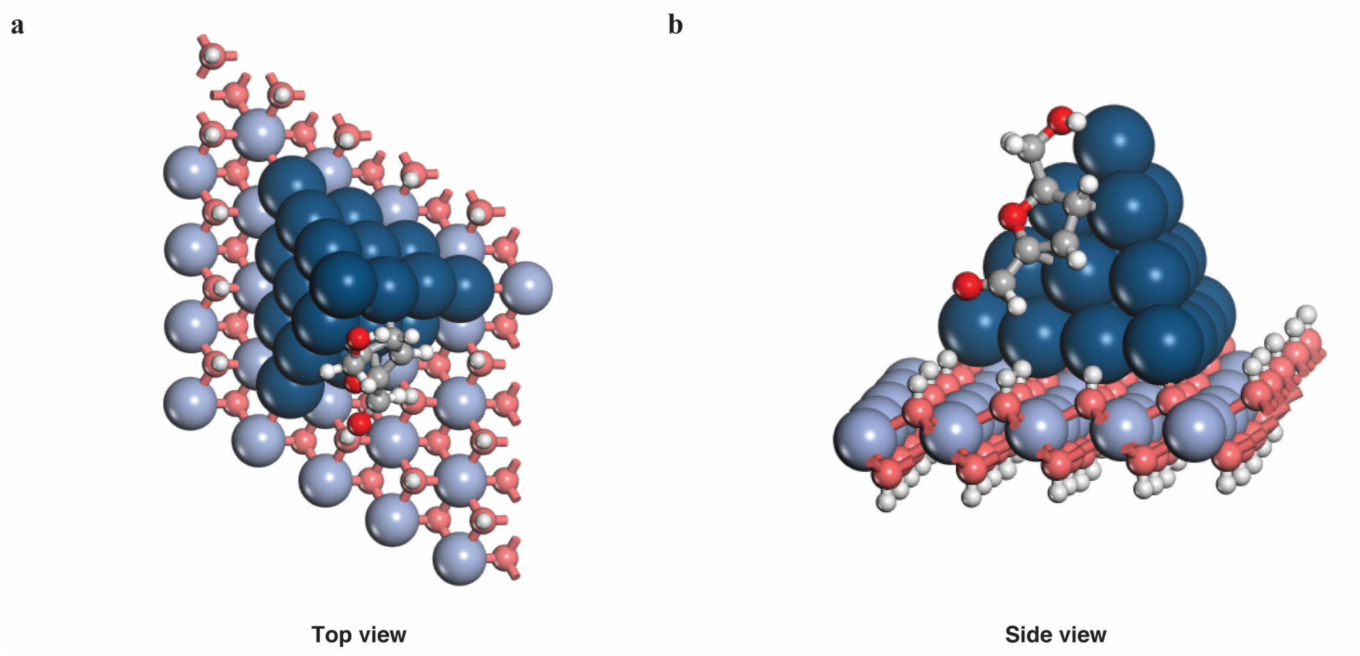

Supplementary Fig. S44 | The models of HMF adsorbed on the Pd/Ni(OH)$_2$ catalyst. a, Top view. **b,** side view.

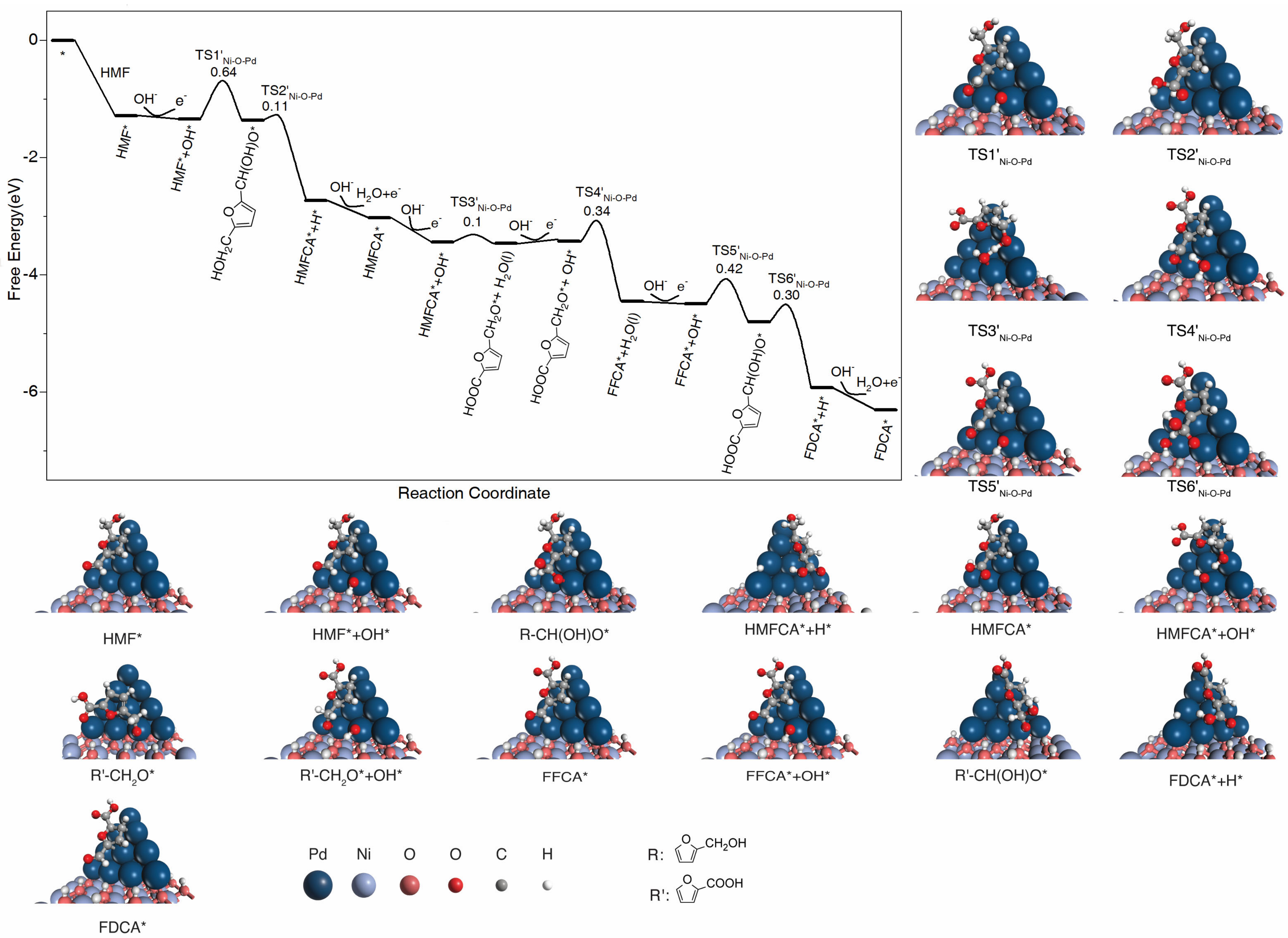

Free Energy(eV)
Reaction Coordinate
0
-2
-4
-6
*
HMF
OH-
e-
TS1'Ni-O-Pd
0.64
TS2'Ni-O-Pd
0.11
HMF*
HMF*+OH*
CH(OH)O*
HOH2C
HMFCA*+H*
OH-
H2O+e-
HMFCA*
OH-
e-
TS3'Ni-O-Pd
0.1
HMFCA*+OH*
CH2O*+ H2O(l)
HOOC
OH-
e-
CH2O*+ OH*
HOOC
TS4'Ni-O-Pd
0.34
FFCA*+H2O(l)
OH-
e-
FFCA*+OH*
TS5'Ni-O-Pd
0.42
TS6'Ni-O-Pd
0.30
CH(OH)O*
HOOC
OH-
H2O+e-
FDCA*+H*
FDCA*
TS1'Ni-O-Pd
TS2'Ni-O-Pd
TS3'Ni-O-Pd
TS4'Ni-O-Pd
TS5'Ni-O-Pd
TS6'Ni-O-Pd
HMF*
HMF*+OH*
R-CH(OH)O*
HMFCA*+H*
HMFCA*
HMFCA*+OH*
R'-CH2O*
R'-CH2O*+OH*
FFCA*
FFCA*+OH*
R'-CH(OH)O*
FDCA*+H*
FDCA*
Pd
Ni
O
O
C
H
R:
CH2OH
R':
COOH

Supplementary Fig. S45 | Mechanism of HMFOR on Pd/Ni(OH)$_2$ catalyst. DFT calculations showed that HMFOR at Ni^{2+}-O-Pd interfaces followed an optimal reaction path similar to that of the pristine Pd catalyst, namely HMF*→R-CHO-OH*→HMFCA*→R'-CH_2O*→FFCA*→R'-CHO-OH*→FDCA*. Comparing Pd(111) and the Ni^{2+}-O-Pd interface, we find that the former favors aldehyde group electrooxidation while the latter favors HMFCA electrooxidation. The RDS barriers for aldehyde group electrooxidation in HMF and FFCA on Pd(111) increased from 0.42 ($TS1_{Pd}$) to 0.64 eV ($TS1'_{Ni\text{-}O\text{-}Pd}$) and from 0.32 ($TS5_{Pd}$) to 0.42 eV ($TS5'_{Ni\text{-}O\text{-}Pd}$), respectively, when compared with the Ni^{2+}-O-Pd interface. In contrast, the RDS barrier (C-H bond activation) for the HMFCA conversion to FFCA over the Ni^{2+}-O-Pd interface (0.34 eV) was much lower than on the Pd(111) surface (0.55 eV). These results show that the oxidation of the aldehyde group of HMF will preferentially occur on the Pd^0 atoms of the Pd/Ni(OH)$_2$ catalyst. In contrast, the oxidation of the hydroxymethyl group of HMFCA will preferentially occur at Ni^{2+}-O-Pd interfaces. Our results suggest that the prepared Pd/Ni(OH)$_2$ catalyst combined the advantages of Pd^0 sites and Ni^{2+}-O-Pd interfaces, working synergetically with a tandem mechanism to produce FDCA from HMFOR selectively.

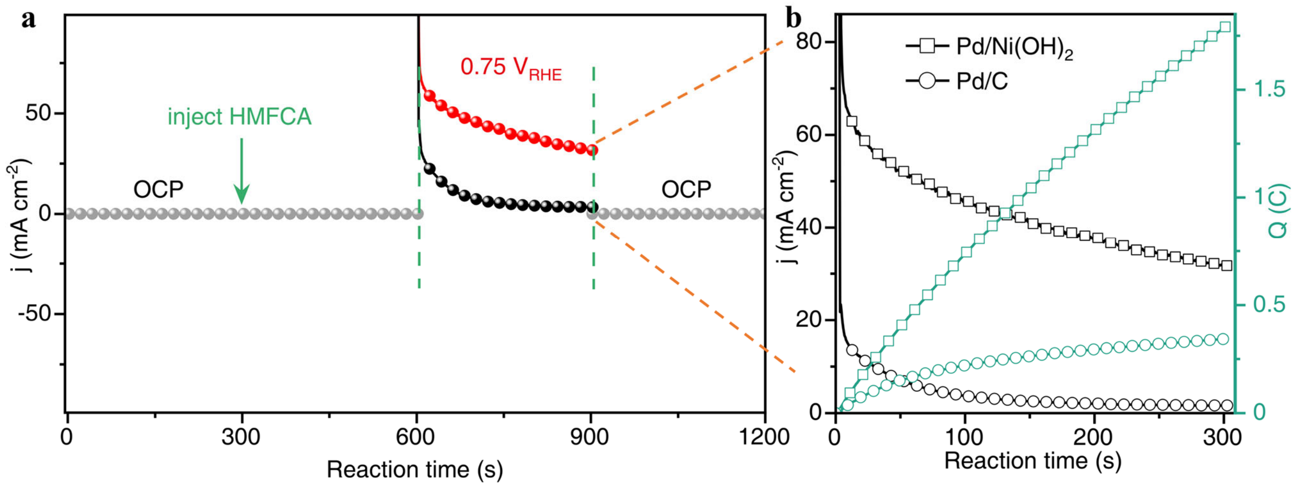

Supplementary Fig. S46 | Potential step curves of the Pd/Ni(OH)$_2$ and Pd/C electrodes for the electrooxidation of HMFCA. a, Activity of Pd/Ni(OH)$_2$ and Pd/C for HMFCA electrooxidation. **b,** The curves of charge passed (Q) versus the reaction time (t) from chronoamperometry for Pd/Ni(OH)$_2$ and Pd/C during HMFCA electrooxidation. The potential step curves demonstrated that the Pd/Ni(OH)$_2$ catalyst showed higher activity than the Pd/C catalyst for the electrooxidation of HMFCA with 95% iR corrected.

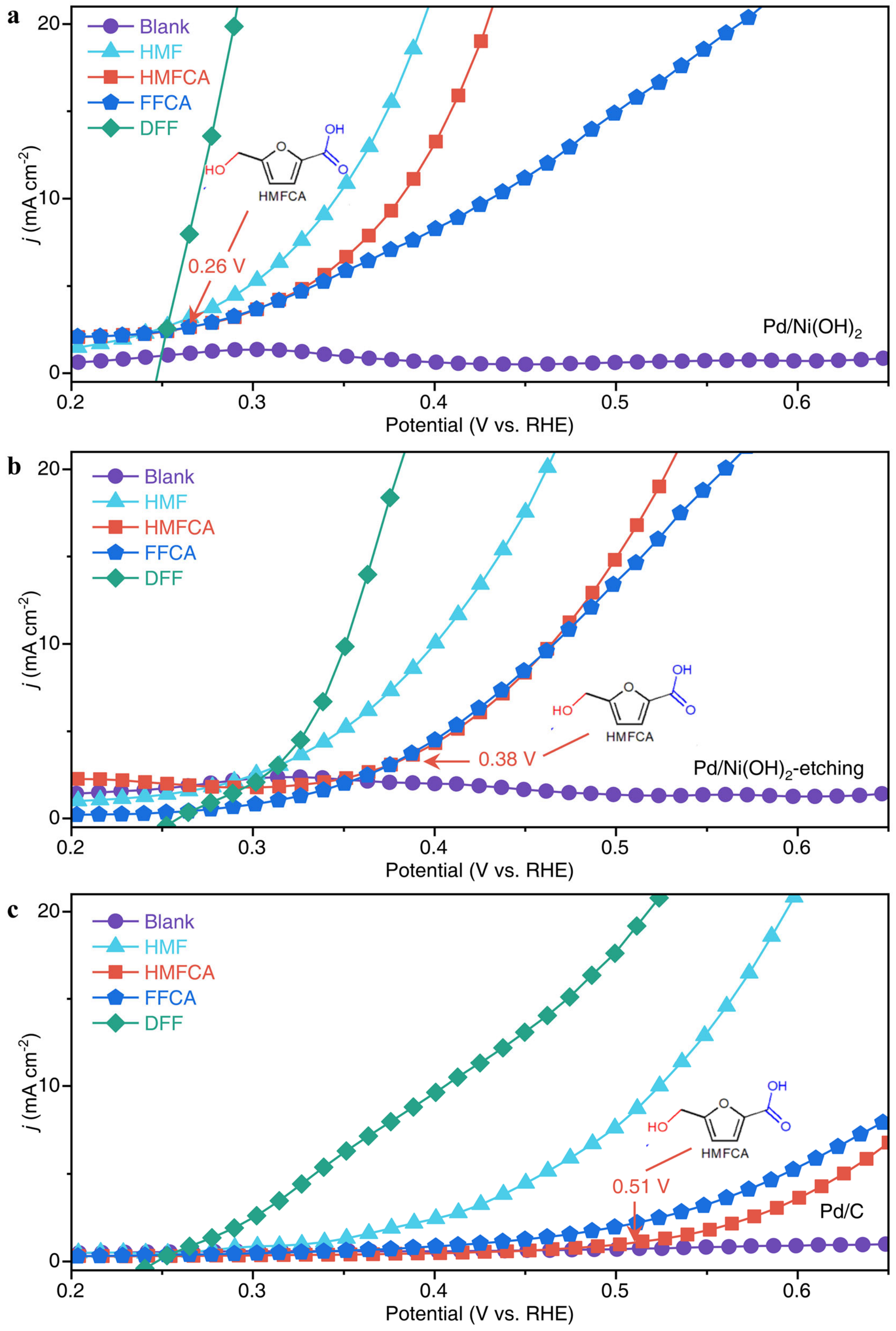

Supplementary Fig. S47 | Comparison of the onset potentials (at 1 mA/cm^2) of different catalysts for the electrooxidation of HMF and its reaction intermediates. a, Pd/Ni(OH)$_2$, **b,** Pd/Ni(OH)$_2$-etching, and **c,** Pd/C in Ar-saturated 1 M KOH + 50 mM reactant solution with 95% iR corrected. The Pd/Ni(OH)$_2$ catalyst greatly enhanced the onset potentials of HMF and its reaction intermediates when compared with the catalysts of Pd/Ni(OH)$_2$-etching and Pd/C.

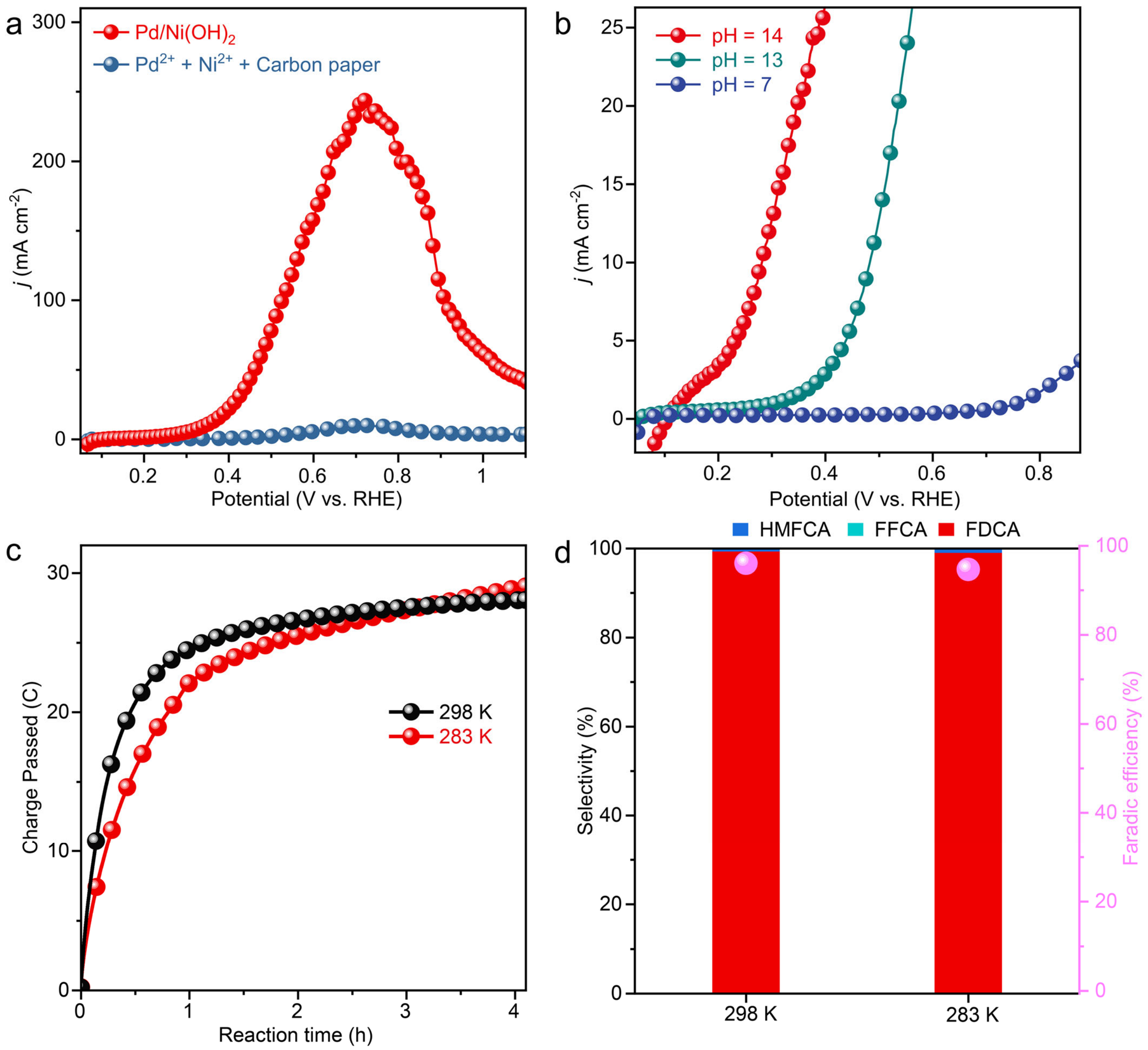

Supplementary Fig. S48 | The controlled experiments of HMFOR over Pd/Ni(OH)$_2$. a, Polarization curves collected on Pd/Ni(OH)$_2$ catalyst and carbon paper with Ni or Pd species in the electrolyte for HMFOR, respectively, with 95% iR corrected. **b,** Controlled experiments of pH dependence were conducted using electrolytes (1 M KOH, 0.1 M KOH, 1 M PBS solution) with different pH values to determine the pH dependence of HMF electrooxidation on Pd/Ni(OH)$_2$ catalyst for HMFOR. **c,** Charge consumption on Pd/Ni(OH)$_2$ catalyst under 0.75 V (vs. RHE) oxidation potentials in Ar-saturated 1 M KOH + 5 mM HMF at different reaction temperatures. **d,** Product selectivity and Faradaic efficiency at different reaction temperatures.

Note: We evaluated the HMF oxidation performance using carbon paper as the working electrode, where equivalent amounts of Ni and Pd (comparable to the loading of Ni and Pd on the working electrode in the manuscript) were dissolved in the electrolyte solution. As shown in Supplementary Fig. S48a, the performance of HMF oxidation was inferior to that of the heterogeneous electrode with Pd/Ni(OH)$_2$ catalyst loaded on the carbon paper, demonstrating that the active sites were Ni^{2+}-O-Pd interfaces rather than the Ni and Pd in the electrolyte solution. Besides, controlled experiments using electrolytes with different pH values show that high pH values facilitate HMFOR at Ni^{2+}-O-Pd active sites (Supplementary Fig. S48b). Moreover, the catalytic performance of HMF oxidation over the Pd/Ni(OH)$_2$ catalyst was evaluated at a relative temperature of 10 ℃ (283 K). Compared to the catalytic performance obtained at room temperature, the rate and product selectivity of HMF oxidation did not significantly decrease at the potential of 0.75 V vs RHE (Supplementary Fig. S48c). The selectivity and Faradaic efficiency achieved for the 6e$^-$ product of FDCA remained consistently high, with approximately 100% selectivity (99.4% FDCA selectivity and 96.1%

Faradaic efficiency at 298 K, 99.1% FDCA selectivity and 95.1% Faradaic efficiency at 283 K) (Supplementary Fig. S48d). This indicates that the rate-determining step of HMFCA oxidation is potential-dependent. Adjusting the voltage of the HMF oxidation reaction can facilitate C-H bond activation during HMFCA oxidation, resulting in a higher production rate and selectivity towards FDCA.

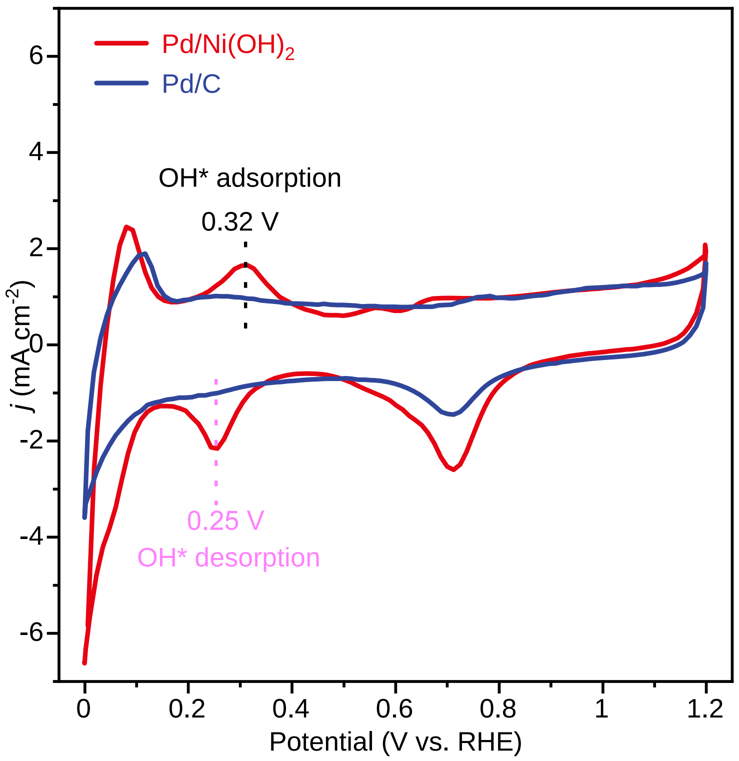

Supplementary Fig. S49 | Study of the Ni^{2+}-O-Pd interfacial effect for OH accumulation on Pd surface. Cyclic voltammetry curves for $Pd/Ni(OH)_2$ and Pd/C were collected in 1 M KOH with a scanning rate of 5 mV/s. The cyclic voltammetry curve of $Pd/Ni(OH)_2$ showed a more significant hydroxide adsorption/desorption than Pd/C catalysts, indicating that the Ni^{2+}-O-Pd interface can effectively promote the enrichment of OH on the Pd at the interface, so that *OH can be provided to HMFOR more efficiently.

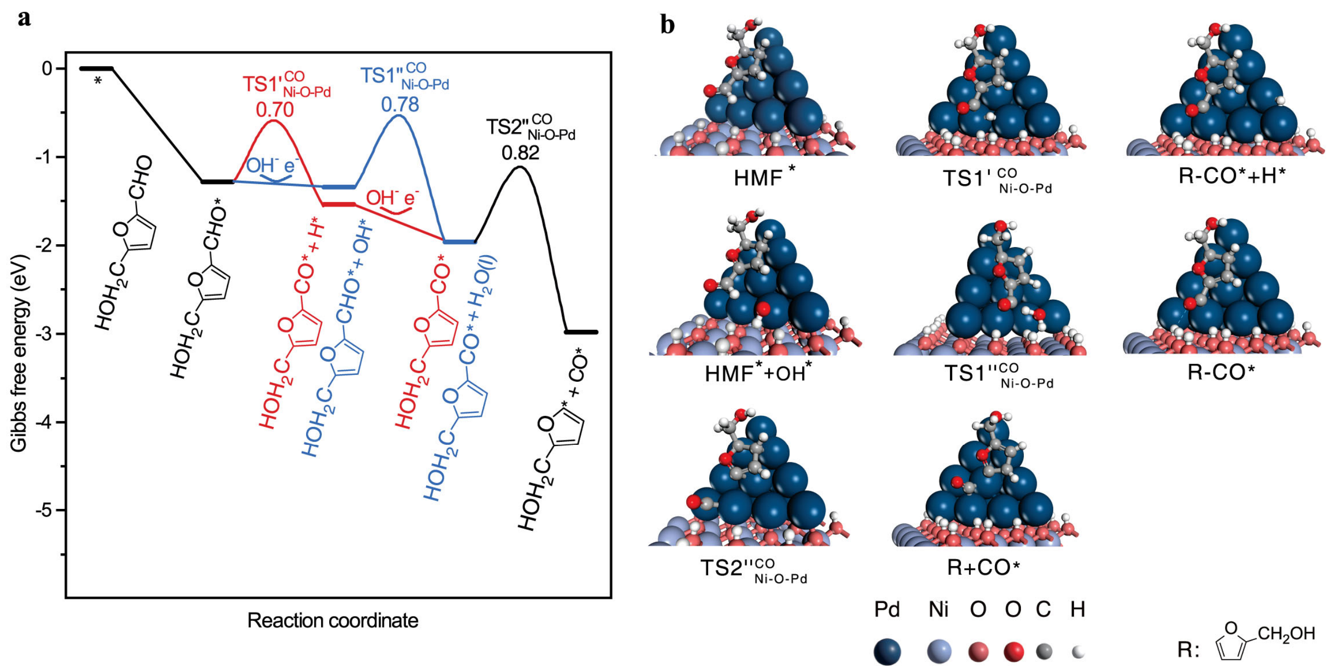

Supplementary Fig. S50 | HMF decarbonylation to generate CO on $Pd_{20}/Ni(OH)_2$. **a**, Energies of intermediates and transition states of HMF decarbonylation on $Pd_{20}/Ni(OH)_2$ from DFT calculations. **b**, The corresponding structures of the intermediates and transition states.

There are two reaction pathways for HMF decarbonylation to produce the intermediate of R-CO*, which is followed by the C-C bond breaking (decarbonylation) of R-CO* to generate CO* with an energy barrier of 0.82 eV ($TS2''^{CO}_{Ni-O-Pd}$). R-CO* can be generated from the direct dehydrogenation of R-CHO* with an energy barrier of 0.70 eV ($TS1'^{CO}_{Ni-O-Pd}$) or OH*-assisted C-H scission of R-CHO* with an energy barrier of 0.78 eV ($TS1''^{CO}_{Ni-O-Pd}$).

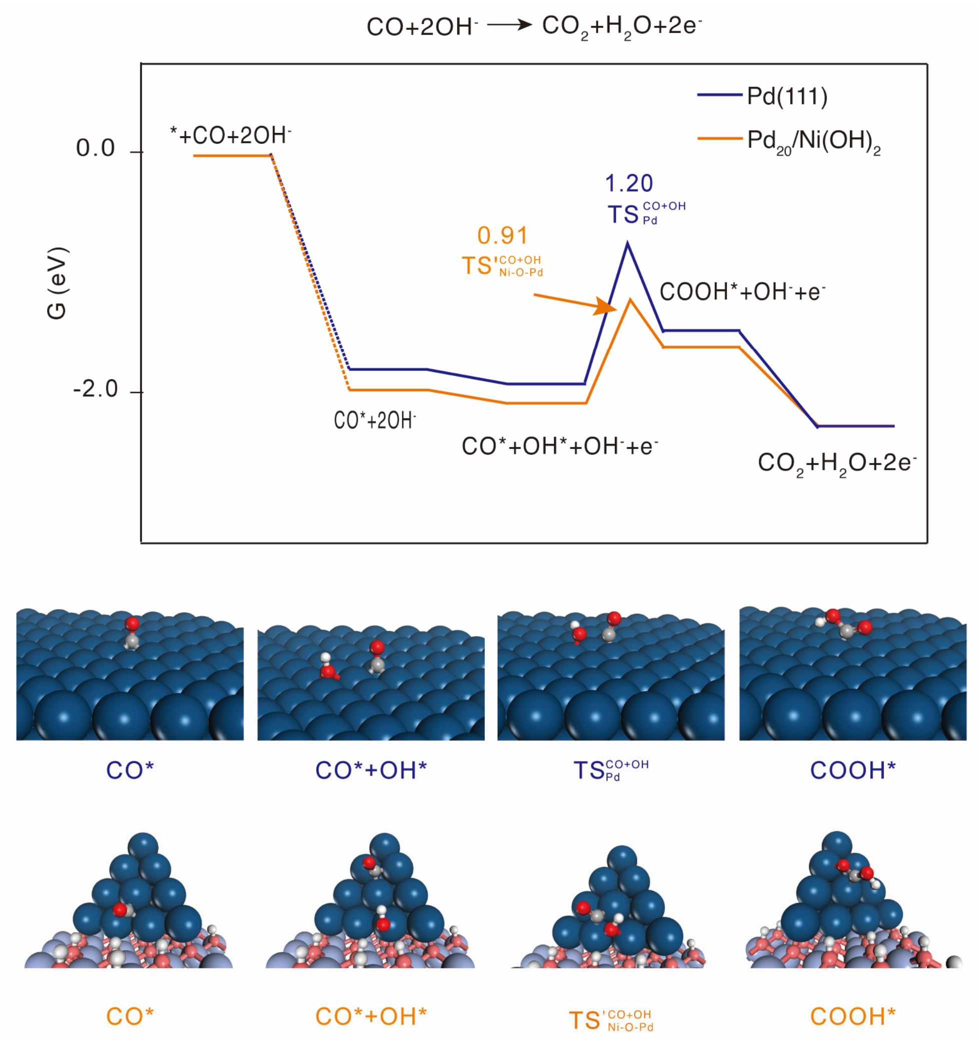

Supplementary Fig. S51 | Comparison of CO removal energy barrier on bare Pd and Pd/Ni(OH)$_2$ surfaces. The adsorbed CO* can be removed from the Pd surface by coupling with OH* to form CO_2 via a COOH* intermediate. The Ni^{2+}-O-Pd interfaces displayed a higher activity than the Pd(111) surface towards CO oxidation with lower oxidation potential (Supplementary Fig. S24) as well as the lower energy barrier (0.91 ($TS'^{CO+OH}_{Ni-O-Pd}$) vs. 1.20 eV (TS^{CO+OH}_{Pd})) (Supplementary Table S10).

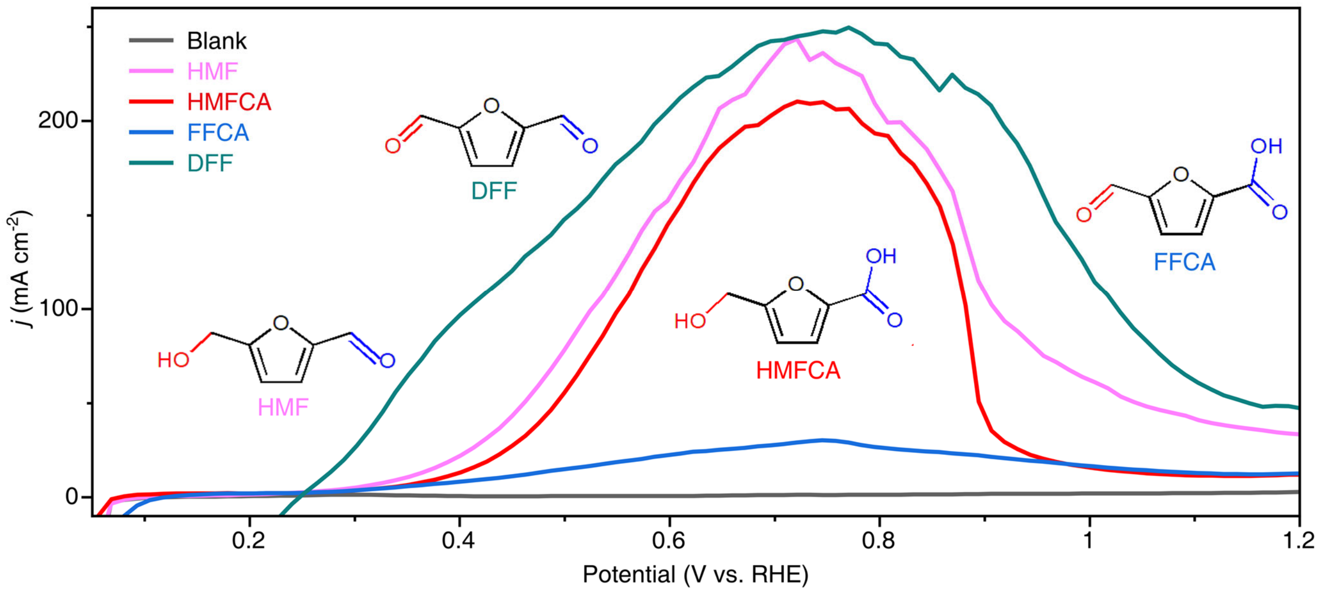

Supplementary Fig. S52 | Polarization curves for the Pd/Ni(OH)$_2$ catalyst during electrooxidation of HMF and its reaction intermediates. The activity of Pd/Ni(OH)$_2$ for electroxidation of HMF, HMFCA, FFCA, and DFF was tested in Ar-saturated 1 M KOH + 50 mM reactants solution with 95% iR corrected. The electrolyte for the controlled experiment was an Ar-saturated 1 M KOH solution.

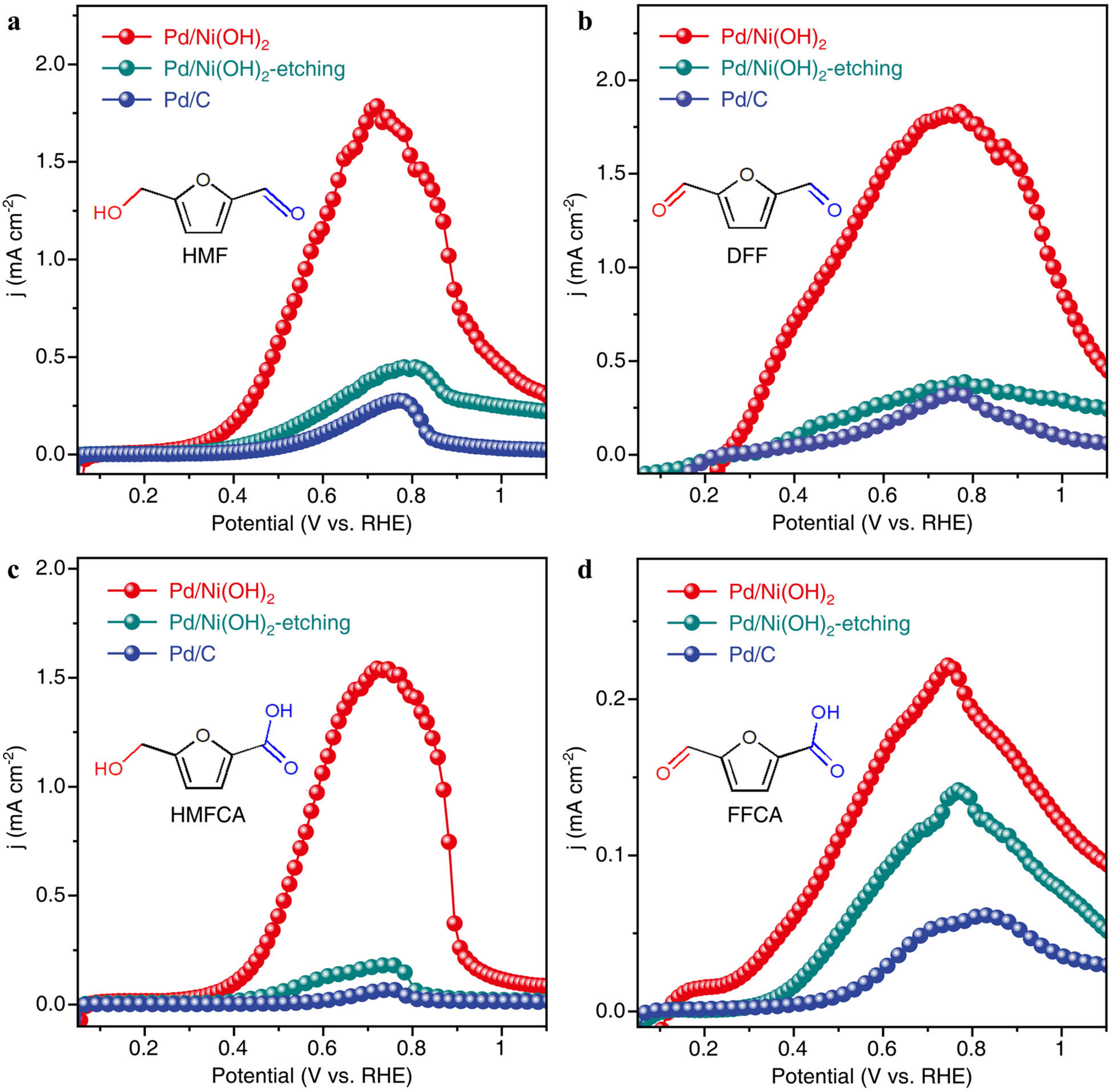

Supplementary Fig. S53 | Comparison of the specific activity on different catalysts during electrooxidation of HMF and its reaction intermediates. Polarization curves were normalized by ECSA measured from different catalysts. The specific activity comparison of Pd/Ni(OH)2, Pd/Ni(OH)2-etching, and Pd/C for electrooxidation of HMF (**a**), HMFCA (**b**), DFF (**c**), and FFCA (**d**) in Ar-saturated 1 M KOH + 50 mM reactant solution.

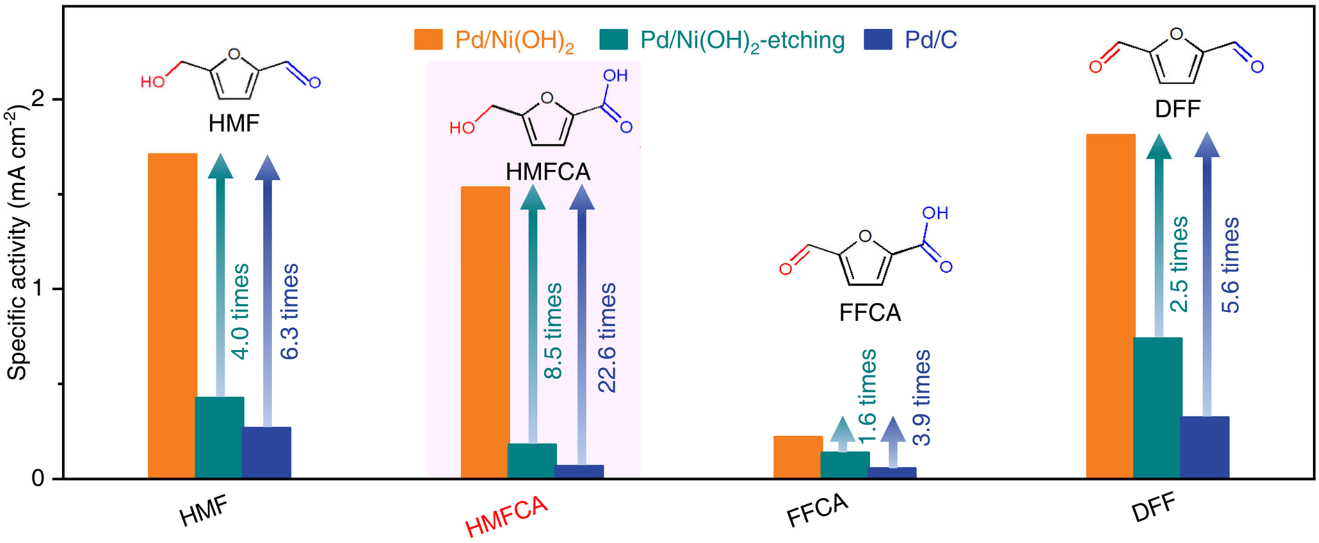

5

Supplementary Fig. S54 | Comparison of specific activity of different catalysts for the electrooxidation of HMF and its reaction intermediates at 0.75 V versus RHE.

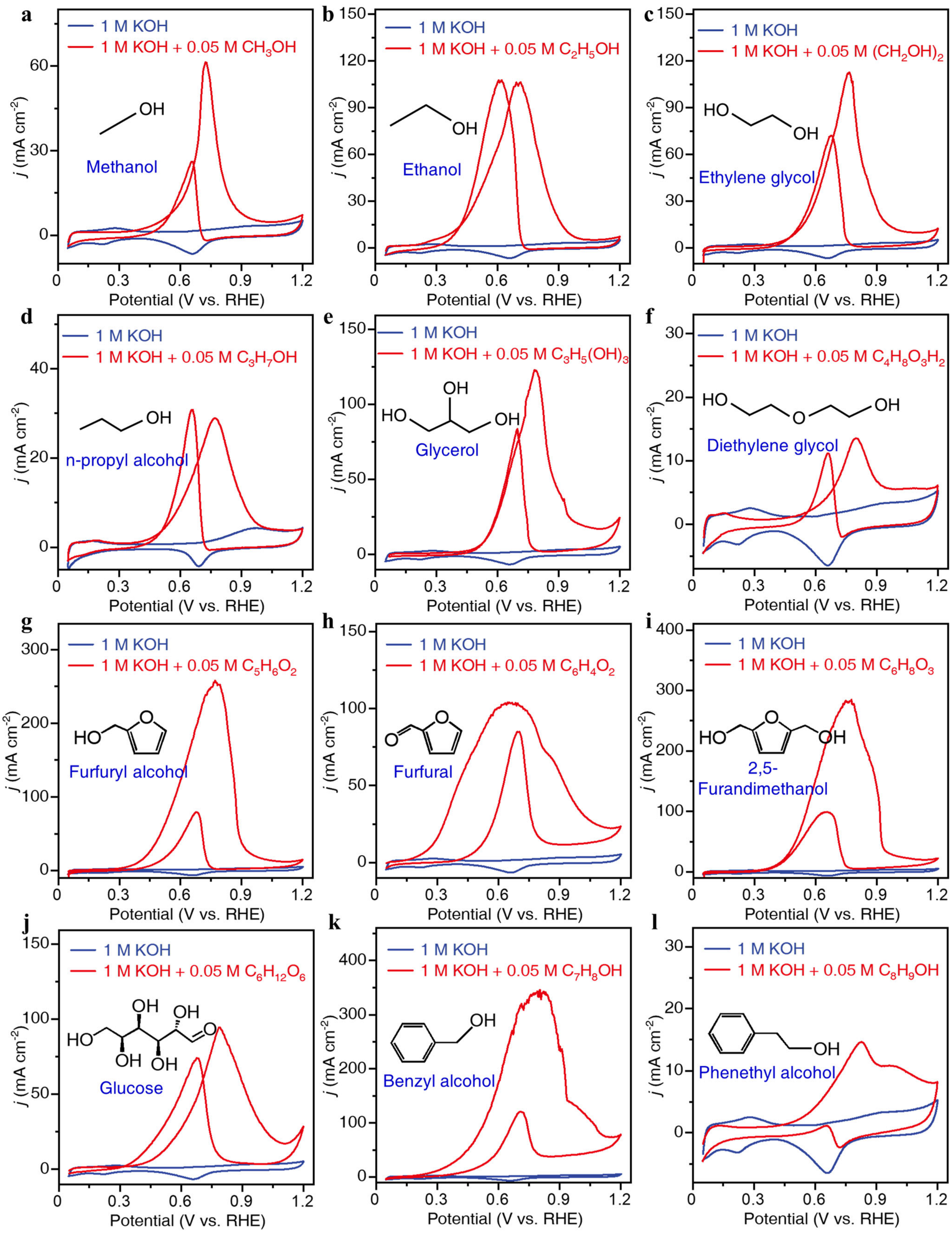

Supplementary Fig. S55 | Versatility of the Pd/Ni(OH)$_2$ catalyst over different alcohol-based or furan-based substances. As a demonstration of the versatility of the Pd/Ni(OH)$_2$ catalyst, we show that the catalyst can selectively electrooxidize many other alcohols (**a**, methanol; **b**, ethanol; **c**, ethylene glycol; **d**, n-propanol; **e**, glycerol; **f**, diethylene glycol; **g**, furfuryl alcohol; **h**, furfural; **i**, furandimethanol; **j**, glucose; **k**, benzyl alcohol; **l**, phenethyl alcohol) to high value-added products (5 mV/s, with 95% iR corrected).

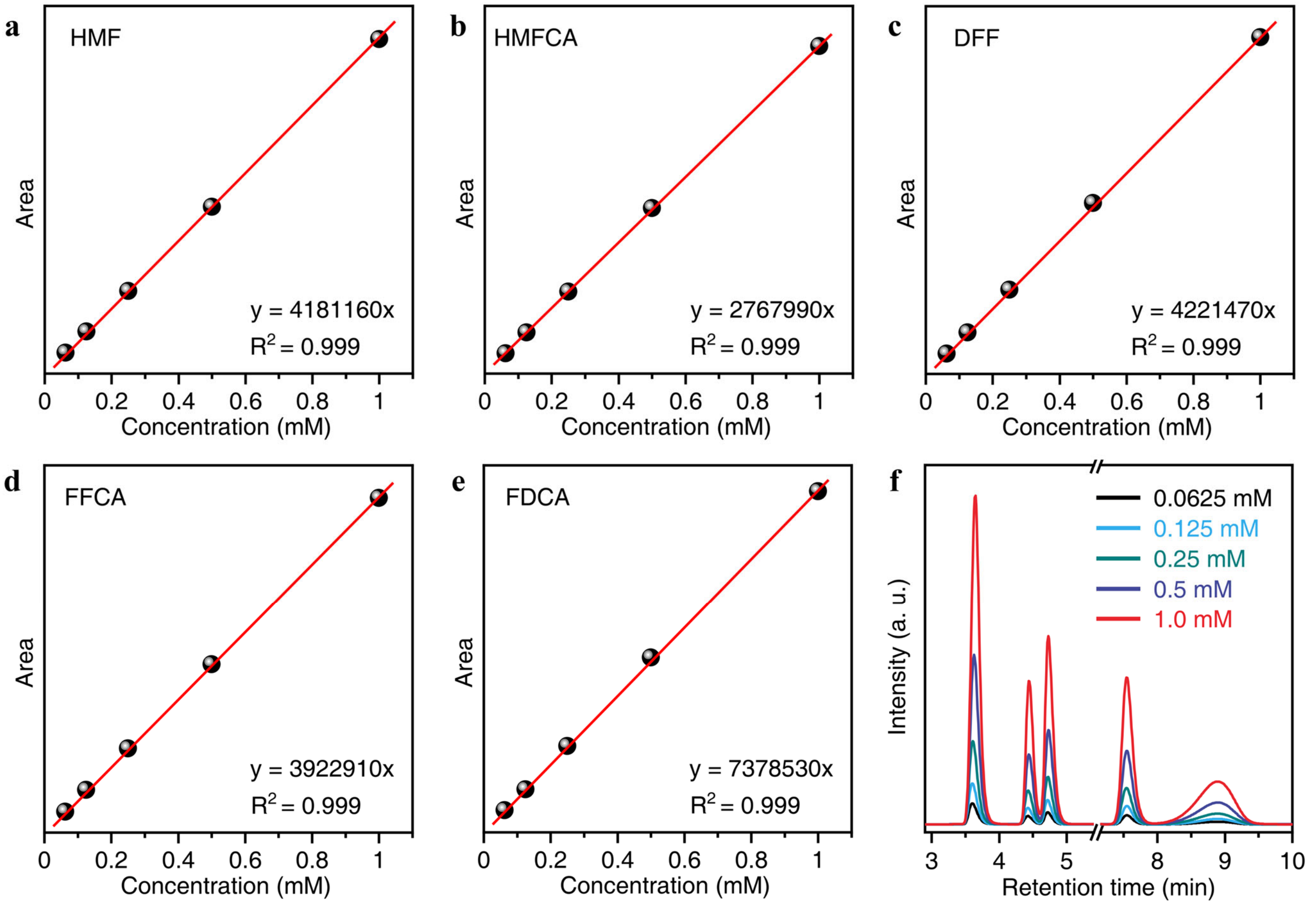

Supplementary Fig. S56 | HPLC standard curves. HPLC standard calibration curves for HMF (**a**), HMFCA (**b**), DFF (**c**), FFCA (**d**), and FDCA (**e**). **f**, The HPLC elution curves for standard mixtures with different concentrations.

Supplementary Table S1 | The main pathways of the electrooxidation of HMF to FDCA.

Intermediates/TS	Pd(111)	Intermediates/TS	$Pd_{20}/Ni(OH)_2$
HMF*	-1.91	HMF*	-1.28
HMF*+OH*	-1.97	HMF*+OH*	-1.34
$TS1_{Pd}$	-1.55	$TS1'_{Ni\text{-}O\text{-}Pd}$	-0.70
R-C(OH)HO*	-1.83	R-C(OH)HO*	-1.36
$TS2_{Pd}$	-1.61	$TS2'_{Ni\text{-}O\text{-}Pd}$	-1.25
HMFCA*+H*	-3.02	HMFCA*+H*	-2.73
HMFCA*	-3.48	HMFCA*	-3.02
HMFCA*+OH*	-3.85	HMFCA*+OH*	-3.44
$TS3_{Pd}$	-3.83	$TS3'_{Ni\text{-}O\text{-}Pd}$	-3.34
R′-CH_2O*+H_2O(l)	-4.03	R′-CH_2O*+H_2O(l)	-3.46
R′-CH_2O*+OH*	-3.95	R′-CH_2O*+OH*	-3.42
$TS4_{Pd}$	-3.40	$TS4'_{Ni\text{-}O\text{-}Pd}$	-3.08
FFCA*+H_2O(l)	-4.76	FFCA*+H_2O(l)	-4.45
FFCA*+OH*	-4.82	FFCA*+OH*	-4.49
$TS5_{Pd}$	-4.50	$TS5'_{Ni\text{-}O\text{-}Pd}$	-4.07
R′-C(OH)HO*	-4.69	R′-C(OH)HO*	-4.80
$TS6_{Pd}$	-4.40	$TS6'_{Ni\text{-}O\text{-}Pd}$	-4.50
FDCA*+H*	-5.95	FDCA*+H*	-5.92
FDCA*	-6.34	FDCA*	-6.30

Supplementary Table S2 | The other pathways of the electrooxidation of HMF to FDCA on Pd(111).

Intermediates/TS	Pd(111)	Intermediates/TS	Pd(111)
R-C(OH)HO*+OH*	-1.97	HMFCA*+OH*	-3.52
TS2′$_{Pd}$	-1.63	TS3′$_{Pd}$	-2.75
HMFCA*+H_2O(l)	-3.48	R′-CH(OH)*+H_2O(l)	-4.22
TS1′$_{Pd}$	-1.25	R′-CH(OH)*+OH*	-4.59
R-CO*+H*	-2.00	TS4″$_{Pd}$	-4.55
R-CO*	-2.36	R′-C(OH)HO*+OH*	-4.80
TS2″$_{Pd}$	-1.90	TS6′$_{Pd}$	-4.44
R*+CO*	-2.99	TS5′$_{Pd}$	-4.16
RCO*+OH*	-2.49	R′-CO+H*	-5.15
TS2‴$_{Pd}$	-2.03	TS5″$_{Pd}$	-4.16
TS1‴$_{Pd}$	-1.27	R′-CO+H_2O(l)	-5.51
TS4′$_{Pd}$	-3.34	R′-CO*+OH*	-5.48
FFCA*+H*	-4.29	TS6″$_{Pd}$	-5.05

Supplementary Table S3 | The other pathways of the electrooxidation of HMF to FDCA on $Pd_{20}/Ni(OH)_2$.

Intermediates/TS	$Pd_{20}/Ni(OH)_2$
$TS1'_{Ni\text{-}O\text{-}Pd}$	-0.56
R-CO*+H_2O(l)	-2.00
R-C(OH)HO*+OH*	-1.43
$TS2'_{Ni\text{-}O\text{-}Pd}$	-0.86
R`-C(OH)HO*+OH*	-4.45
$TS6'_{Ni\text{-}O\text{-}Pd}$	-4.20

Supplementary Table S4 | HMF and FFCA decarbonylation to generate CO on pristine Pd(111) surface.

Intermediates/TS	Pd(111)	Intermediates/TS	Pd(111)
HMF*	-1.91	FFCA*	-4.76
$TS1'^{CO}_{Pd}$	-1.25	$TS5'^{CO}_{Pd}$	-4.23
R-CO*+H*	-2.00	R′-CO*+H*	-5.15
HMF*+OH*	-1.97	FFCA*+OH*	-4.82
$TS1''^{CO}_{Pd}$	-1.27	$TS5''^{CO}_{Pd}$	-4.16
R-CO*+H_2O	-2.36	R′-CO*+H_2O	-5.51
$TS2''^{CO}_{Pd}$	-1.90	$TS6'''^{CO}_{Pd}$	-4.75
R*+CO*	-3.02	R′*+CO*	-5.83

Supplementary Table S5 | Best-fit parameters for the Pd K-edge EXAFS data for the Pd/Ni(OH)$_2$ catalyst.

Sample	shell	CN	R(Å)	Debye-Waller factor ($\Delta\sigma^2$)	R-factor
Pd/Ni(OH)$_2$	Pd-O	1.2±0.2	2.01±0.020	0.007±0.0014	1.3*10^{-2}
	Pd-Pd	3.6±0.7	2.72±0.027	0.012±0.0024	

Supplementary Table S6 | Best-fit parameters for the Ni K-edge EXAFS data for the Pd/Ni(OH)$_2$ catalyst.

Sample	shell	CN	R(Å)	Debye-Waller factor ($\Delta\sigma^2$)	R-factor
Pd/Ni(OH)$_2$	Ni-O	2.1±0.4	1.83±0.018	0.013±0.0026	7*10^{-3}
	Ni-OH	3.9±0.8	2.04±0.020	0.005±0.001	

Supplementary Table S7 | ECSA of different catalysts measured by CO-stripping.

Entry	Catalyst	ECSA of Catalysts ($m^2\ g_{Pd}^{-1}$)	Pd mass Loading (cm^2/mg_{Pd})	Electrode area (cm^2)	ECSA of electrodes (cm^2)
1	Pd/Ni(OH)$_2$	30.3	0.9	0.5	136.4
2	Pd/Ni(OH)$_2$-etching	56.1	1.0	0.5	274.7
3	Pd/C	38.9	1.0	0.5	201.7

Supplementary Table S8 | The performance of $Pd/Ni(OH)_2$ catalysts for the electrooxidation of HMF to FDCA.

Catalysts	Electrolyte used for LSV	Scan rate of LSV (mV/s)	E_{Onset} (V vs. RHE)	E_j = 10 mA cm^{-2}/20 mA cm^{-2} (V vs RHE)	Volume/Electrolyte for chronoamperometry	potential for FDCA production (V vs. RHE)	FDCA selectivity/yield (%)	FE[a] (%)
						**0.4**	**87.1/67.2**	**93.1**
**$Pd/Ni(OH)_2$**	**1.0 M KOH+50 mM HMF**	**5**	**0.22**	**0.34/0.39**	**10 mL /1.0 M KOH+5 mM HMF**	**0.5**	**95.9/86.6**	**94.1**
						**0.6**	**~100/97.3**	**93.9**

[a] FE was the Faradaic efficiency of HMFOR. In addition, the potentials for FDCA production are with 95% iR corrected.

Note: An electrochemical workstation (BioLogic EC-Lab) with a traditional three-electrode H-cell was used for electrochemical measurements. HMFOR performance test was conducted in a three-electrode H-type Cell, with a Pt-foil as the counter electrode and Hg/HgO as the reference electrode, and the voltages were 95% iR-corrected. Catalyst inks were prepared by dispersing a catalyst $Pd/Ni(OH)_2$-5.0 mg in a mixture of isopropanol (0.5 mL) and pure water (0.45 mL) and Nafion (0.05 mL) followed by sonication for 0.5 h. Subsequently, 300 μL of catalyst ink was dropped onto the carbon paper (1×0.5 cm^2, 3.0 mg cm^2). After drying at room temperature, the catalyst-covered carbon paper was utilized as the working electrode in HMFOR measurements. The pH of the electrolytes of 1 M KOH+ 50 mM HMF solution was 13.8 ± 0.2 (determined by pH meter). Chronoamperometry tests were performed at different working potentials in an Ar-saturated 1 M KOH+ 5 mM HMF solution (10 mL). The cathode and anode were separated by a proton exchange membrane (PEM, Nafion 117). PEM is suitable for a wide pH range (0-14), and we found through controlled experiments that it is more effective than anion exchange membranes (AEM) in preventing HMF and products from crossing the membrane to the cathode. Moreover, the theoretical maximum transfer coulomb amount was 28.95 C and would not cause a change in pH value. The curves of charge consumption (Q) versus reaction time (t) at each working potential were collected until the reaction finished. An inert gas (Ar) was purged through the electrolyte during the chronoamperometry test to avoid the side reaction of oxygen reduction reaction (ORR). The volume of the reaction solution was measured and collected after the performance test.

Supplementary Table S9 | Bader charges analysis of Pd atoms at $Pd_{20}/Ni(OH)_2(001)$ interface by DFT Calculation.

Pd_{20}	Valence state
Bottom layer (4th layer)	0.35, 0.35, 0.22, 0.23, 0.22, 0.24, 0.21, 0.14, 0.14, 0.14
3rd layer	0.00, 0.02, -0.11, -0.11, 0.00, -0.10
2nd layer	-0.01, -0.03, -0.01
1st layer	-0.06

Supplementary Table S10 | HMF decarbonylation to generate CO on $Pd_{20}/Ni(OH)_2$.

Intermediates/TS	$Pd_{20}/Ni(OH)_2$
HMF*	-1.28
$TS1'^{CO}_{Ni-O-Pd}$	-0.58
R-CO*+H*	-1.54
HMF*+OH*	-1.34
$TS1''^{CO}_{Ni-O-Pd}$	-0.56
R-CO*+H_2O	-1.96
$TS2''^{CO}_{Ni-O-Pd}$	-1.14
R*+CO*	-2.98

Supplementary Table S11 | Comparison of the reaction energy barriers for the electrooxidation of HMF via decarbonylation pathway and carboxylate pathways.

Intermediates/TS	Pd(111)	$Pd_{20}/Ni(OH)_2$
$CO^{*}+2OH^{-}$	-1.80	-1.98
$CO^{*}+OH^{*}+OH^{-}+e^{-}$	-1.93	-2.09
TS	-0.73	-1.18
$COOH^{*}+OH^{-}+e^{-}$	-1.48	-1.62

Supplementary Table S12 | Metal contents in different catalysts measured by ICP-OES.

Entry	Catalysts	Metal loading (wt.%)	
		Pd/%	Ni/%
1	$Pd/Ni(OH)_2$	29.98	11.32
2	$Pd/Ni(OH)_2$-etching	32.61	2.29
3	Pd/C	15.52	-
4	$Pd/Ni(OH)_2$-after stability test	31.46	12.01
