# Supplementary material for: Enhanced electrocatalytic biomass oxidation at low voltage by Ni2+-O-Pd interfaces
Source: Nat Commun. 2024 Jul 13;15:5899. doi: 10.1038/s41467-024-50325-w (PMC11246419; doi:10.1038/s41467-024-50325-w)
Supplement: Supplementary file 2 — Peer Review File [file 41467_2024_50325_MOESM2_ESM.pdf]

nature portfolio

Peer Review File

Enhanced electrocatalytic biomass oxidation at low voltage by Ni2+-O-Pd interfaces

Open Access This file is licensed under a Creative Commons Attribution 4.0 International License, which permits use, sharing, adaptation, distribution and reproduction in any medium or format, as long as you give appropriate credit to the original author(s) and the source, provide a link to the Creative Commons license, and indicate if changes were made. In the cases where the authors are anonymous, such as is the case for the reports of anonymous peer reviewers, author attribution should be to 'Anonymous Referee' followed by a clear attribution to the source work. The images or other third party material in this file are included in the article's Creative Commons license, unless indicated otherwise in a credit line to the material. If material is not included in the article's Creative Commons license and your intended use is not permitted by statutory regulation or exceeds the permitted use, you will need to obtain permission directly from the copyright holder. To view a copy of this license, visit http://creativecommons.org/licenses/by/4.0/.

REVIEWER COMMENTS

Reviewer #1 (Remarks to the Author):

The submitted manuscript from Pei et al presents a novel electrocatalyst Pd/Ni(OH)2 with abundant Ni2+-O-Pd interfaces for the electrocatalytic oxidation of HMF to FDCA at lower potential than many other reported electrocatalyst could achieve. Overall, the design, synthesis, and characterization of the new catalyst were well discussed. Its improved catalytic performance was also thoroughly investigated, aided by theoretical computations. This reviewer recommends its possible acceptance by Nature Communications if the authors could address the following concerns.

1. This work can greatly benefit from additional in situ spectroscopic analysis to further elucidate the mechanisms involved in the observed phenomena. The authors only briefly mentioned the in situ IR results for CO identification. In fact, this section could be substantially expanded to provide more insights regarding the adsorption and formation of substrates, intermediates, products during various catalytic conditions.

2. The identification of products obtained in Supplementary Fig. S55 requires further clarification. Detailed analysis, possibly through advanced spectroscopic techniques, would strengthen the findings and provide a clearer understanding of the reaction pathways and product formation.

3. Lines 19-20 on page 4, "...by the increased the concentration..." should be "...by increasing the concentration..."

Reviewer #2 (Remarks to the Author):

In the submitted work, the authors carry out a combined theoretical and experimental study on the electrochemical oxidation of HMF with Pd nanoparticles deposited on Ni(OH)2 sheets. The composited materials shows an earlier onset potential and higher activity as compared to the individual components. The enhanced activity was attributed to Pd acting as active sites and interfacial OH groups at the Pd/Ni interface participating in the reaction. In all, this is a through work and stands to bring important insights to the field of electrocatalysis. I would recommend publishing this work after addressing several comments below.

The authors discuss the formation of CO and its role in potentially poisoning the catalyst surface. The show CO with GC measurements but I would be interested in seeing whether that is a significant amount of CO or not. How many CO molecules actually formed relative to the expected amount of HMF molecules that were reacted after their 45 minute experiment?

The authors propose that the HMFCA oxidation step is the limitation on Pd and support this with LSV measurements of HMF, HMFCA, FFCA and DFF reactants. As the aldehyde oxidation reactions are occurring at much lower potential, do the authors know if they actually proceed through the ‘anodic H2 generation’ mechanism as has been reported on Cu and Ag, or is this still the conventional mechanism?

This can readily be verified with H2 measurement in the anodic chamber post electrolysis.

For the samples in which the Ni components were etched away, I would recommend the authors to carry out ICP measurements (or equivalent) to verify that all of the Ni is indeed removed as opposed to remaining in small quantities.

Reviewer #3 (Remarks to the Author):

I have read the article with interest and it is a timely and relevant topic. My main aspect is that I am not convinced what the active phase is and if the contribution originates from the heterogeneous electrode surface, or that it originates from Ni or Pd which is present in the electrolyte solution. I believe that the authors should evaluate this point; but also to evaluate if at relatively low temperature the oxidation reaction can take place. The article is well-written and structured and contains a lot of new aspects, but as such I am not yet convinced that what is reported indeed stems from the electrocatalytic surface. Hence, major revisions for this work.

Reply to Reviewer #1

We appreciate all the comments from the reviewer, which are very helpful for us to improve the manuscript. According to the reviewer's suggestions, we have carefully revised our manuscript as follows:

Comment 1: The submitted manuscript from Pei et al presents a novel electrocatalyst Pd/$Ni(OH)_2$ with abundant Ni^{2+}-O-Pd interfaces for the electrocatalytic oxidation of HMF to FDCA at lower potential than many other reported electrocatalyst could achieve. Overall, the design, synthesis, and characterization of the new catalyst were well discussed. Its improved catalytic performance was also thoroughly investigated, aided by theoretical computations. This reviewer recommends its possible acceptance by Nature Communications if the authors could address the following concerns.

Response: We appreciate the reviewer for the valuable suggestions and recommendations. The manuscript has been revised carefully according to the reviewer's comments.

Comment 2: This work can greatly benefit from additional in situ spectroscopic analysis to further elucidate the mechanisms involved in the observed phenomena. The authors only briefly mentioned the *in-situ* IR results for CO identification. In fact, this section could be substantially expanded to provide more insights regarding the adsorption and formation of substrates, intermediates, products during various catalytic conditions.

Response: We appreciate the reviewer's thoughtful suggestions. To further elucidate the mechanisms involved in the HMFOR on Ni^{2+}-O-Pd interfaces, the *in-situ* IR results were expanded and further analyzed to provide more insights regarding the adsorption and formation of substrates, intermediates, and products during various catalytic conditions. By carrying out an *in-situ* infrared test of adsorption of intermediates on Pd/C and the comparison of relevant literature (*Nat. Commun.* 2023, 14, 8395; *Adv. Mater.* 2022, 34, e2204089; *Adv. Mater.* 2023, 35, 2304133; *J. Am. Chem. Soc.* 2023, 145, 32, 17577-17587; *Angew. Chem. Int. Ed.* 2023, 62, e202311696; *ACS Catal.* 2022, 12, 18, 11226-11238; *Appl. Catal. B-Environ.* 2021, 299, 120669), the attribution of different peak positions in our work was further identified.

As per the reviewer's valuable suggestions, in the revised manuscript, the section of *in-situ* FTIR was further discussed in detail: During chronoamperometry at different potentials (from 0.1 ~ 1.0 V versus RHE) (with 0.1 V intervals) in 1 M KOH solution containing HMF, *in-situ* FTIR analysis demonstrated the prodcution of CO species (1875 cm^{-1}) on both Pd/C and Pd/Ni$(OH)_2$ catalysts, indicating that the decarbonylation reaction occurred on both Pd/C and Pd/Ni$(OH)_2$ catalysts. Moreover, the adsorption of intermediates and the formation of FDCA products during different voltages were further identified. As shown in Fig. R1 (Supplementary Fig. S8) and referenced in *Nat. Commun.* 2023, 14, 8395 (Fig. R2), the adsorption of HMFCA intermediate (1217 cm^{-1}, 1350 cm^{-1}) and formation of FDCA (1590 cm^{-1}) were observed on both Pd/C and Pd/Ni$(OH)_2$ catalysts. It is worth noting that the adsorption signal of HMFCA intermediate (1217 cm^{-1}, 1350 cm^{-1}) was firstly observed at a lower voltage of 0.3 V on Pd/Ni$(OH)_2$ catalyst than on Pd/C catalyst (begin at 0.4 V), illustrating the higher HMFOR efficient on Pd/Ni$(OH)_2$ catalyst than Pd/C catalyst. Additionally, the product signal of FDCA (1590 cm^{-1}) also emerged at 0.3 V on Pd/Ni$(OH)_2$ catalyst, indicating that the oxidation of HMFCA was more facile compared to Pd/C, a finding consistent with the results obtained from HPLC analysis. The signal intensity of HMFCA and FDCA were enhanced by increasing HMFOR voltage, illustrating that HMFOR was a potential dependent electrooxidation reaction. The absence of the FFCA intermediate detection, consistent with the HPLC findings, can be attributed to its susceptibility to oxidation on the Pd catalyst during HMFOR.

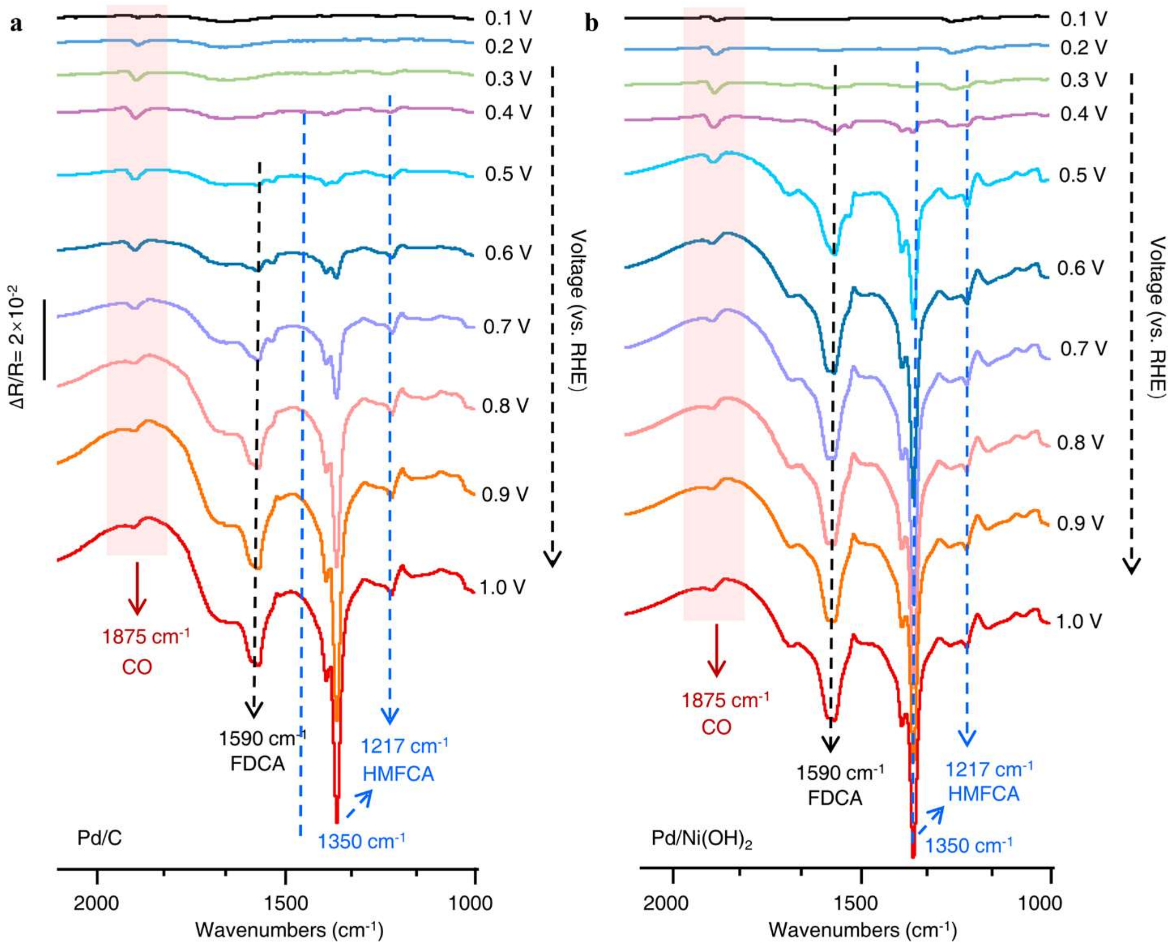

Fig. R1 (Supplementary Fig. S8). Electrochemical in-situ FTIR studies on Pd/Ni(OH)$_2$ and Pd/C catalysts during HMFOR.

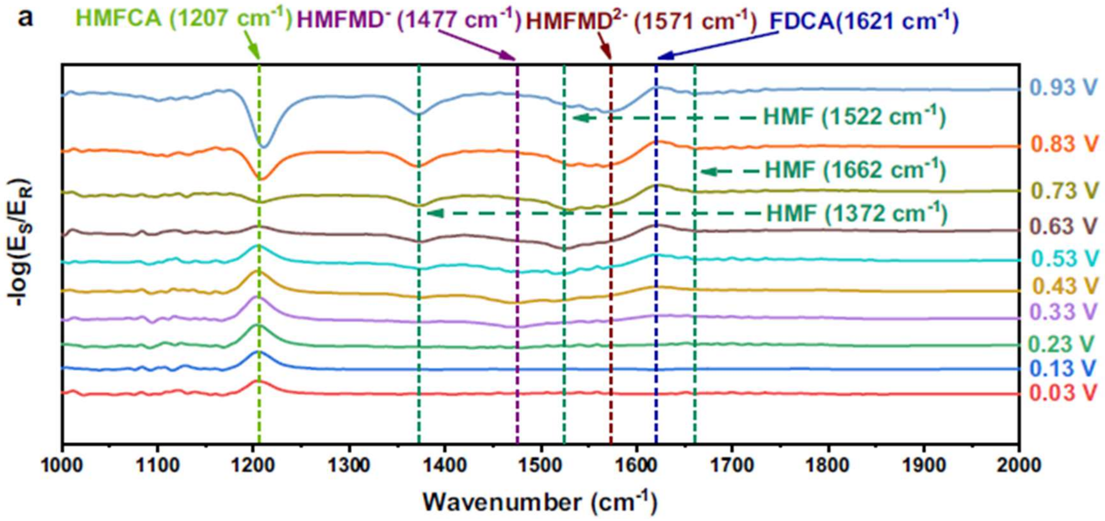

Fig. R2. Electrochemical *in-situ* FTIR spectra of HMF oxidation reactions on gold. (*Nat. Commun.* 14, 8395, 2023)

Comment 3: The identification of products obtained in Supplementary Fig. S55 requires further clarification. Detailed analysis, possibly through advanced spectroscopic techniques, would strengthen the findings and provide a clearer understanding of the reaction pathways and product formation.

Response: We appreciate the reviewer for the comments. In order to strengthen the findings of this investigation, chronoamperometry tests of the different reaction substrates depicted in Supplementary Fig. S55 were conducted. Advanced spectroscopic methodologies, including high-performance liquid chromatography (HPLC) and nuclear magnetic resonance spectroscopy (H-NMR), were utilized to offer a more comprehensive understanding of the reaction pathway and product formation through detailed product analysis. As shown in Fig. R3 and Fig. R4, the versatility of the Pd/Ni(OH)$_2$ catalyst over different alcohol-based substances under low oxidation potential (0.75 V vs. RHE) was evaluated. Quantitative product analysis revealed that the electrochemical oxidation of different alcohol-based substrates on Pd/Ni(OH)$_2$ catalysts predominantly yield corresponding high-value acids instead of undergoing carbon-carbon bond cleavage (except for methanol oxidation). Detailed information can be found in Fig. R3 and Fig. R4. The identification of products closely aligns with the conclusion presented in the manuscript, emphasizing the selective electrooxidation of HMF to yield FDCA. This also enhances our understanding of substrate universality in selective oxidation under low potentials. This finding is conducive to advancing the development of efficient catalysts in the field of selective alcohol oxidation at low potentials, offering promising avenues for the design and optimization of catalysts tailored for various alcohol oxidation reactions (< 1.0 V).

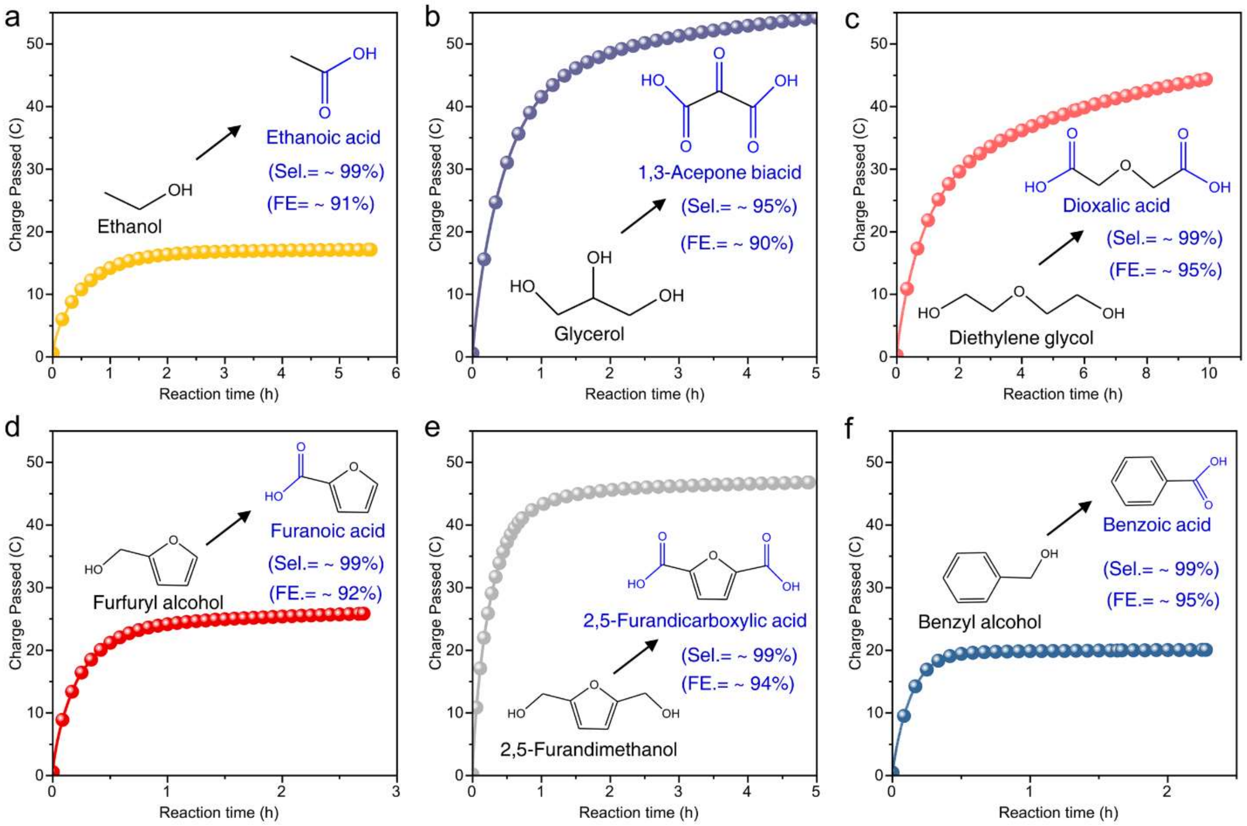

Fig. R3. Versatility of the Pd/Ni(OH)$_2$ catalyst over different alcohol-based substances. The performances on Pd/Ni(OH)$_2$ catalyst on different alcohol-based substances were carried out at the potential of 0.75 V (vs. RHE) with 5 mM substances (10 mL) in Ar-saturated 1 M KOH. The curves of charge passed (Q) versus reaction time (t) of each reaction were recorded till the end of the reaction.

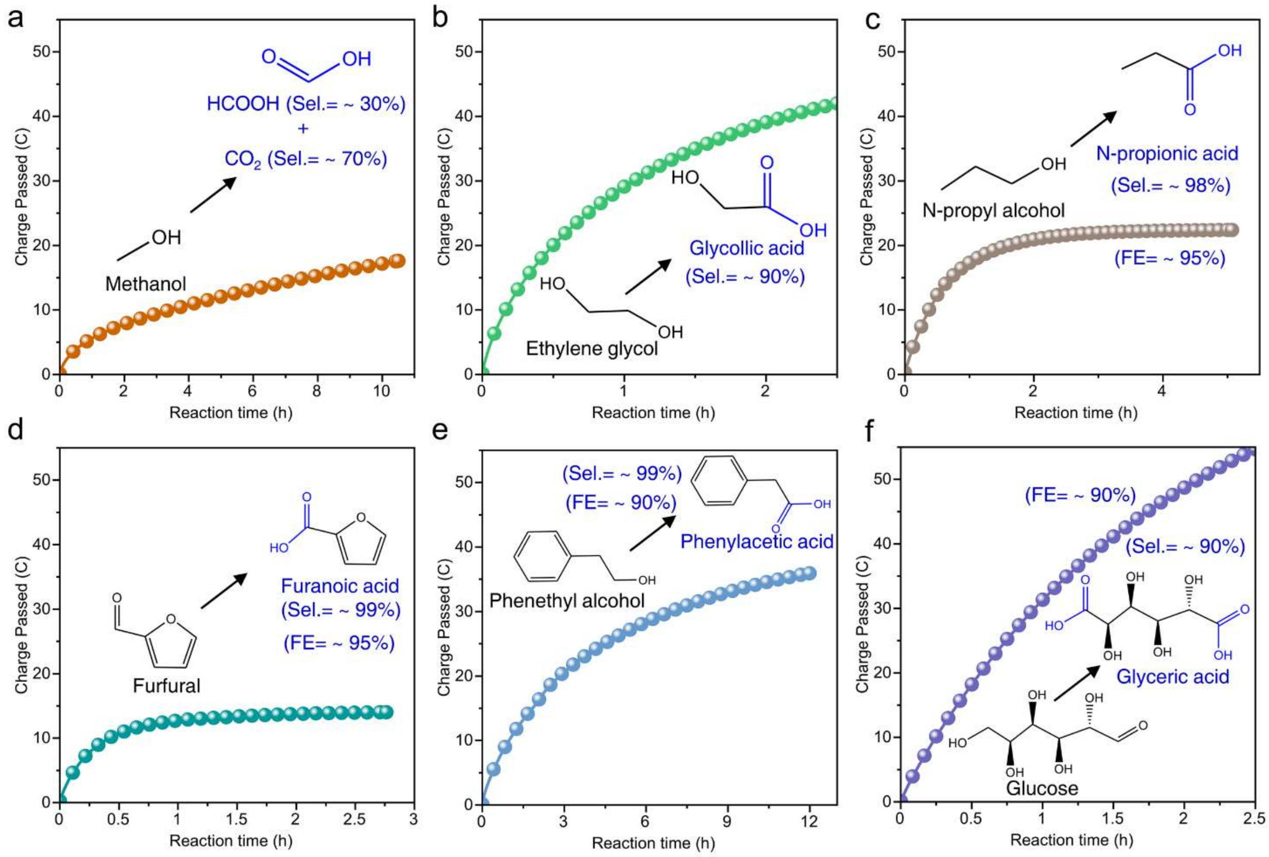

Fig. R4. Versatility of the Pd/Ni(OH)2 catalyst over different alcohol-based substances. The performances on Pd/Ni(OH)2 catalyst on different alcohol-based substances were carried out at the potential of 0.75 V (vs. RHE) with 5 mM substances (10 mL) in Ar-saturated 1 M KOH. The curves of charge passed (Q) versus reaction time (t) of each reaction were recorded till the end of the reaction.

Comment 4: Lines 19-20 on page 4, "...by the increased the concentration..." should be "...by increasing the concentration..."

Response: We appreciate the reviewer's suggestion. The sentence mentioned by the reviewer has been revised in accordance with the reviewer's comments as follow:

"Moreover, the Pd/Ni(OH)2 catalyst can also work efficiently and stably for more than 24 hours without deactivation under the current density of ~380 mA/cm^2 in a flow cell reactor (flow cell voltage 1.05 V) by increasing the concentration of HMF (125 mM HMF in 1 M KOH) and the flow rate (2.5 mL/min) of the electrolyte."

Reply to Reviewer #2

We appreciate all the comments from the reviewer, which are very helpful for us to improve the manuscript. According to the reviewer's suggestions, we have carefully revised our manuscript as follows:

Comment 1: In the submitted work, the authors carry out a combined theoretical and experimental study on the electrochemical oxidation of HMF with Pd nanoparticles deposited on $Ni(OH)_2$ sheets. The composited materials show an earlier onset potential and higher activity as compared to the individual components. The enhanced activity was attributed to Pd acting as active sites and interfacial OH groups at the Pd/Ni interface participating in the reaction. In all, this is a through work and stands to bring important insights to the field of electrocatalysis. I would recommend publishing this work after addressing several comments below.

Response：We appreciate the reviewer for the valuable suggestions and recommendations. The manuscript has been revised carefully according to the reviewer's comments.

Comment 2：The authors discuss the formation of CO and its role in potentially poisoning the catalyst surface. The show CO with GC measurements but I would be interested in seeing whether that is a significant amount of CO or not. How many CO molecules actually formed relative to the expected amount of HMF molecules that were reacted after their 45-minute experiment?

Response: We appreciate the reviewer for the comments. The generation of CO suggested that decarbonylation reaction occurred during HMFOR on the Pd/C catalyst, potentially leading to the poisoning of the Pd catalyst surface by CO. To ascertain the Faraday efficiency of CO, indicating the actual number of CO molecules formed relative to the expected amount of HMF molecules during the HMFOR experiment, in-situ gas chromatography detection was conducted at different potentials to detect CO. Additionally, the presence of H_2 was also evaluated. Background measurements were initially taken, where no CO was detected during chronoamperometry in a solution of 1 M KOH with 0 mM HMF. In contrast, the intensity of CO signal increased significantly in the electrolyte containing 1 M KOH with 50 mM HMF, as shown

in Fig. R5. As shown in Fig. R5a-c, the generation of both CO and H_2 were *in-situ* detected by GC during running chronoamperometry (CA) at 0.40 and 0.75 V versus RHE in 1 M KOH solution + 50 mM HMF for HMFOR on Pd/C and Pd/Ni(OH)$_2$ catalysts. The Faradaic efficiency of both CO and H_2 at 0.40 V is higher than at 0.75 V in a solution of 1 M KOH + 50 mM HMF for HMFOR on both Pd/C and Pd/Ni(OH)$_2$ catalyst. It is noteworthy that the Faradic efficiency of CO generation reaction on Pd/Ni(OH)$_2$ ($FE_{(CO)}$=0.89% at 0.4 V, $FE_{(CO)}$=0.20% at 0.75 V) catalyst was much lower than that on Pd/C ($FE_{(CO)}$=7.88% at 0.4 V, $FE_{(CO)}$=0.53% at 0.75 V). This demonstrates that the CO generation reaction on Pd was suppressed by the formation of the Ni^{2+}-O-Pd interface, as depicted in Figures R5d-f.

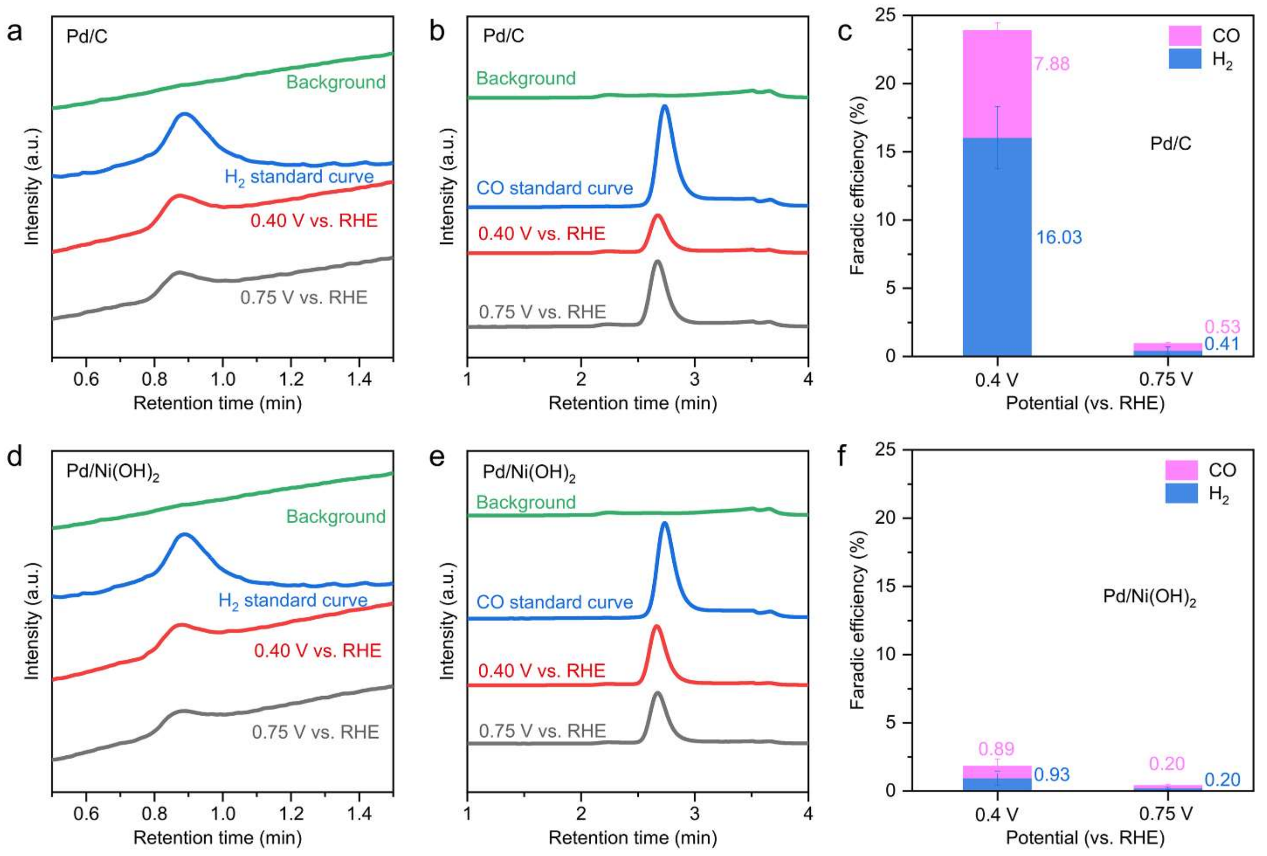

Fig. R5 (Supplementary Fig. S7). *In-situ* detection of CO and H_2 during HMFOR on Pd/C and Pd/Ni(OH)$_2$ catalysts. The generation of CO and H_2 was detected *in-situ* by gas chromatography during running chronoamperometry (CA) at 0.40 and 0.75 V versus RHE in 1 M KOH solution + 50 mM HMF for HMFOR on both Pd/C and Pd/Ni(OH)$_2$ catalyst. **a**, the GC signal of H_2 from Pd/C catalyst. **b**, the GC signal of CO from Pd/C catalyst. **c**, the FE of both CO and H_2 from Pd/C catalyst

under different potentals. **d**, the GC signal of H_2 from $Pd/Ni(OH)_2$ catalyst. **e**, the GC signal of CO from $Pd/Ni(OH)_2$ catalyst. **f**, the FE of both CO and H_2 from $Pd/Ni(OH)_2$ catalyst under different potentals.

Comment 3: The authors propose that the HMFCA oxidation step is the limitation on Pd and support this with LSV measurements of HMF, HMFCA, FFCA and DFF reactants. As the aldehyde oxidation reactions are occurring at much lower potential, do the authors know if they actually proceed through the 'anodic H_2 generation' mechanism as has been reported on Cu and Ag, or is this still the conventional mechanism? This can readily be verified with H_2 measurement in the anodic chamber post electrolysis.

Response: We appreciate the reviewer for the comments. After conducting H_2 measurements with gas chromatorgrahpy (GC) in the anodic chamber post electrolysis (under different potentials of 0.4 and 0.75 V vs RHE, respectively), we did observed the H_2 signal, as demonstrated in Fig. R5 above. The quantity of H_2 produced through HMF dehydrogenation at 0.4 V exceeded that generated at 0.75 V. This implies that the anodic H_2 generation mechanism observed with Cu and Ag also occurred on the surface of Pd catalysts, especially under low oxidation potentials. Nevertheless, the produced H_2 was minimal, and its concentration remained within the range of ppm with the relatively low H_2 Faradic efficiency of 16.3% at 0.4 V and 0.41% at 0.75 V on Pd/C. The suppressed H_2 Faradic efficiency on $Pd/Ni(OH)_2$ catalyst (0.93% at 0.4 V and 0.20% at 0.75 V) further suggests that the predominant mechanism was direct oxidation proposed in the this work rather than the mechanism with anodic H_2 generation.

Comment 4: For the samples in which the Ni components were etched away, I would recommend the authors to carry out ICP measurements (or equivalent) to verify that all of the Ni is indeed removed as opposed to remaining in small quantities.

Response: We appreciate the reviewer's comments. We apologize for the insufficient coverage of the acid etching process and ICP measurement in the manuscript. However, we have already conducted the measurements of ICP-OES, XPS, and EDS-mapping over the $Pd/Ni(OH)_2$-etching

sample. The corresponding data were provided in Supplementary information of Table S12 and Fig. S37. Before acid etching, the Ni loading of Pd/Ni(OH)$_2$ was 11.32 wt%. After the acid etching process, most of the Ni(OH)$_2$ was removed, leaving 2.29 wt% of Ni remaining on the Pd/Ni(OH)$_2$-etching sample. The results from ICP-OES were consistent with the measurements of XPS and EDS-mapping. After the Ni components were etched away, the original abundant Ni^{2+}-O-Pd interfaces were destroyed and the Faradaic efficiency and FDCA selectivity showed a marked decline further demonstrating the key role of Ni^{2+}-O-Pd interfaces for HMFOR. Thanks again for the reviewer's valuable comments.

Supplementary Table S12 | Metal contents in different catalysts measured by ICP-OES.

Entry	Catalysts	Metal loading (wt.%)	
		Pd/%	Ni/%
1	Pd/Ni(OH)$_2$	29.98	11.32
2	Pd/Ni(OH)$_2$-etching	32.61	2.29
3	Pd/C	15.52	-
4	Pd/Ni(OH)$_2$-after stability test	31.46	12.01

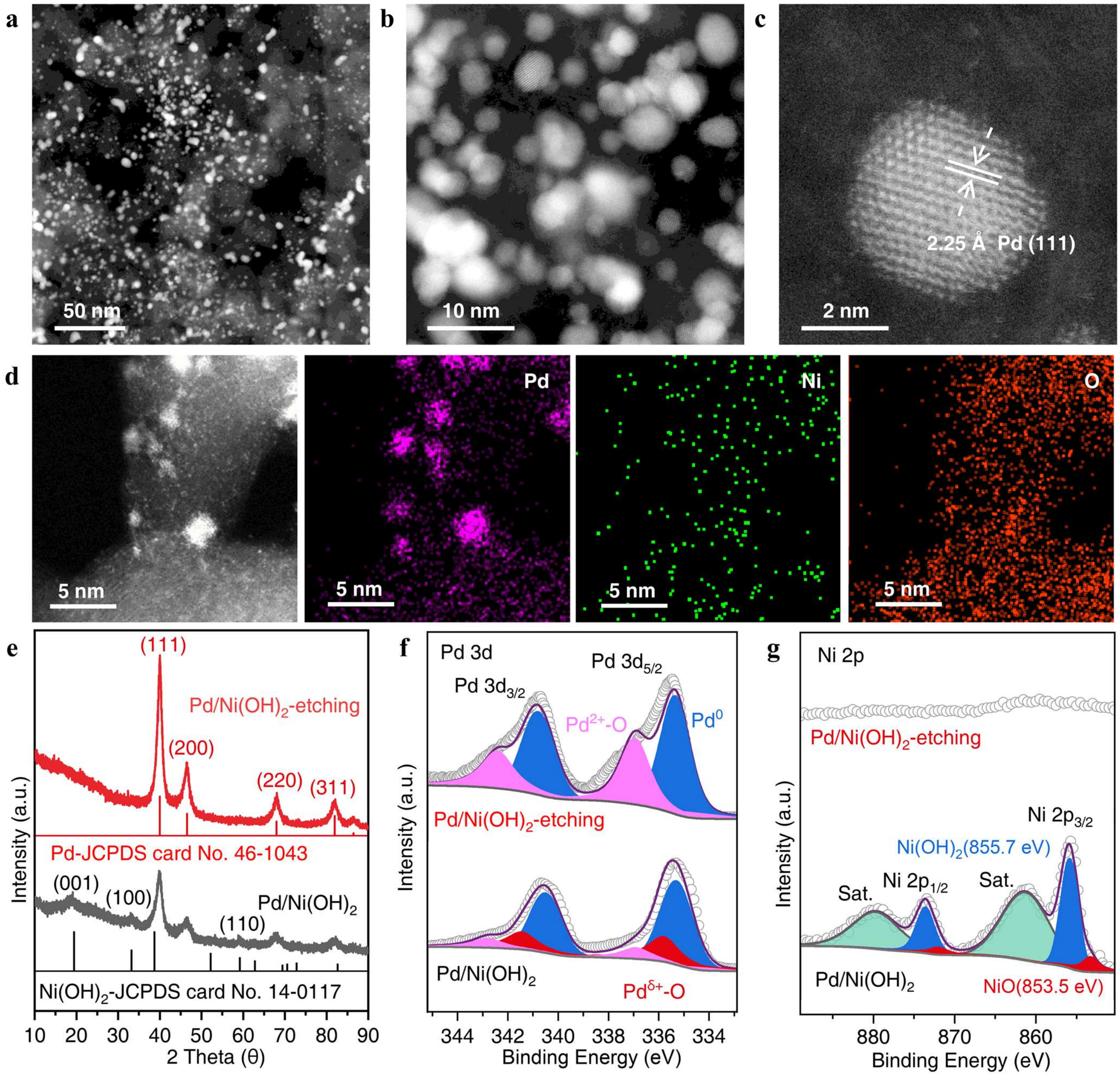

Supplementary Fig. S37 | Structural characterizations of Pd/Ni(OH)$_2$-etching catalyst.

Reply to Reviewer #3

We appreciate all the comments from the reviewer, which are very helpful for us to improve the manuscript. According to the reviewer's suggestions, we have carefully revised our manuscript as follows:

Comment 1: I have read the article with interest and it is a timely and relevant topic.

Response: We appreciate the reviewer's attentive reading and insightful suggestions. Biomass valorization is crucial for future chemical production and energy storage, warranting careful and thorough study.

Comment 2: My main aspect is that I am not convinced what the active phase is and if the contribution originates from the heterogeneous electrode surface, or that it originates from Ni or Pd which is present in the electrolyte solution. I believe that the authors should evaluate this point;

Response: We appreciate the reviewer's comments. We apologize for the lack of adequate control experiments in our study to investigate the active sites in our system. To study the impact of the Ni or Pd in the electrolyte solution, ICP-OES measurement was first performed to determine the concentration of Ni or Pd in the electrolyte solution. The concentrations of Ni and Pd in the electrolyte solution were found to be minimal, as confirmed ICP-OES, which indicated that Ni and Pd were not detected, respectively. This result suggests that the Ni or Pd species in the electrolyte were not the active species during HMF oxidation. Moreover, we also evaluated the HMF oxidation performance using carbon paper as the working electrode, where equivalent amounts of Ni and Pd (comparable to the loading of Ni and Pd on the working electrode in the manuscript) were dissolved in the electrolyte solution. As shown in Fig. R6, the performance of HMF oxidation was inferior to that of the heterogeneous electrode with Pd/$Ni(OH)_2$ catalyst loaded on the carbon paper, demonstrating that the active sites were Ni^{2+}-O-Pd interfaces rather than the Ni and Pd in the electrolyte solution.

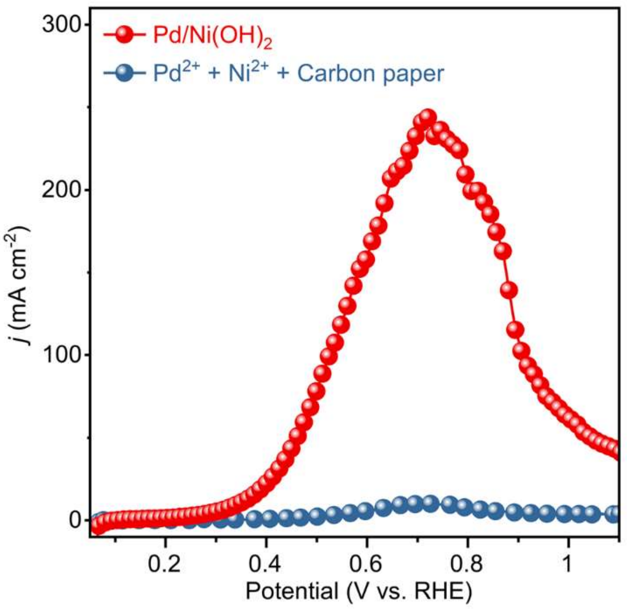

Fig. R6(Supplementary Fig. S48a). Polarization curves recorded on Pd/Ni(OH)$_2$ catalyst and carbon paper with Ni or Pd species in the electrolyte for HMFOR, respectively.

Comment 3: But also to evaluate if at relatively low temperature the oxidation reaction can take place.

Response: We appreciate the reviewer's valuable suggestion. In line with this suggestion, the catalytic performance of HMF oxidation over the Pd/Ni(OH)$_2$ catalyst was evaluated at a relative temperature of 10 °C (283 K). Compared to the catalytic performance obtained at room temperature, both the rate and product selectivity of HMF oxidation were not significantly decreased at the potential of 0.75 V vs RHE (Fig. R7). The selectivity and Faradaic efficiency achieved for the 6e$^-$ product of FDCA remained consistently high, with approximately 100% selectivity (99.4% FDCA selectivity and 96.1% Faradaic efficiency at 298 K, 99.1% FDCA selectivity and 95.1% Faradaic efficiency at 283 K) (Fig. R7). This indicates that the rate-determining step of HMFCA oxidation is potential-dependent. Adjusting the voltage of the HMF oxidation reaction can facilitate C-H bond activation during HMFCA oxidation, resulting in a higher production rate and selectivity towards FDCA. Thanks again for the reviewer's valuable comments.

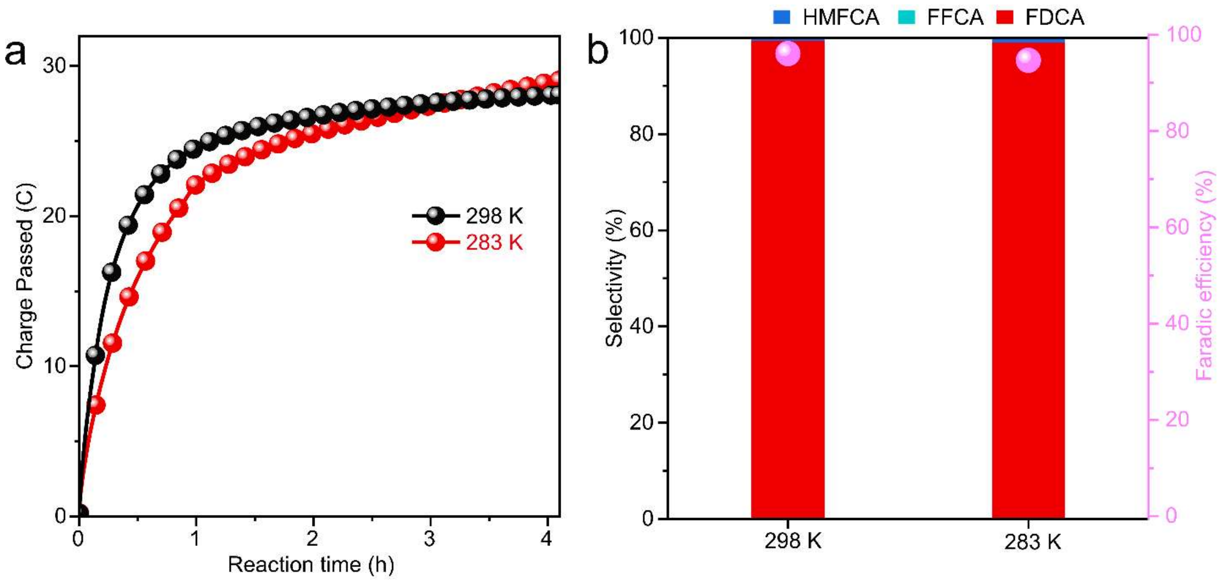

Fig. R7(Supplementary Fig. S48c,d). HMF oxidation on Pd/Ni(OH)$_2$ catalyst under different temperatures. a, Charge consumption on Pd/Ni(OH)$_2$ catalyst under 0.75 V (vs. RHE) oxidation potentials in Ar-saturated 1 M KOH + 5 mM HMF at different reaction temperatures. b, Product selectivity and Faradaic efficiency at different reaction temperatures.

Comment 4: The article is well-written and structured and contains a lot of new aspects, but as such I am not yet convinced that what is reported indeed stems from the electrocatalytic surface. Hence, major revisions for this work.

Response: We appreciate the recognition and appreciation from the reviewer for our work. We apologized that the expression in our manuscript was not clear enough. The main theme of our work is <u>to demonstrate the design and synthesis of the ultrathin Ni(OH)$_2$/Pd (< 2 nm in diameter) catalyst with abundant Ni^{2+}-O-Pd interfaces, and a deep understanding of the mechanism of how Ni^{2+}-O-Pd interfaces realized the best performance for selective HMF oxidation to FDCA, which has never been achieved and reported before and will greatly promote the industrial implementation of biomass in the future.</u> This work not only made a great contribution to the development of biomass valorization but also made a significant fundamental impact on interfacial electrocatalysis. Details are as follows:

First, as we all know, 2,5-Furandicarboxylic acid (FDCA) is a prized value-added chemical from biomass processing and is finding increasing usage as a feedstock for the manufacture of

biodegradable plastics. However, the industrial implementation of FDCA has been hampered for more than two decades due to the low selectivity of the conversion of HMF to FDCA. In this work, we overcome this challenge by developing a Pd/$Ni(OH)_2$ catalyst with abundant Ni^{2+}-O-Pd interfaces which allows electrooxidation of HMF to FDCA with an unprecedented selectivity near 100 % and FDCA yield of 97.3% at 0.6 volts (versus RHE) which is about 0.8 volts lower than state-of-the-art catalysts in 1 M KOH electrolyte under ambient conditions. This is the best performance reported so far for converting HMF to FDCA. Although relatively active and selective electrocatalysts for HMF oxidation have been reported, these catalysts were oxide and hydroxide-based catalysts (Pt/$Ni(OH)_2$, Ir_1/Co_3O_4, Ru_1/NiO, PdO/CuO), which generally follows a nucleophile oxidation reaction mechanism, in which the catalysts needed to be pre-oxidized to generate high-valence active metal species (such as Co^{4+}, Ni^{3+} and so on) at high oxidation potentials (> 1.4 V versus RHE). Unfortunately, HMF oxidation at high oxidation potentials will inevitably result in the oxidation of the carbon substrate of the catalyst/electrode, while also promoting other undesirable side reactions such as oxygen evolution reaction (OER) or the dissolution of electrode materials, hindering the industrial production of FDCA. This work paves the way for the development of biomass valorization, especially the industrial implementation of FDCA.

Second, a deep understanding of the mechanism of HMF oxidation on the surfaces and interfaces of Pd-based catalysts is essential for the achievement of superior performance for the conversion of HMF to FDCA. Although the mechanisms of the electrooxidation alcohol and aldehyde on the surfaces of Pd catalysts have been studied intensively in the area of fuel cells, the main discussions were the C1 pathway (to CO_2) to promote the breaking of the C-C bond, thereby increasing the energy efficiency of the fuel cells. In contrast, the pathways for the electrooxidation of alcohol and aldehyde to acid without C-C bond breaking are favorable in biomass valorization, especially for the conversion of HMF to FDCA. Here, for the first time, a deep understanding of the mechanism of HMF oxidation over Pd and Pd/$Ni(OH)_2$ catalysts was obtained. The rate-determining step (RDS) for HMF to FDCA on Pd is C-H bond activation in 5-hydroxymethyl-2-furancarboxylic acid (HMFCA) oxidation. It's not easy to overcome the energy barrier of C-H bond activation on the pristine Pd surface, leading to low selectivity to FDCA during HMF oxidation. The Ni^{2+}-O-Pd interfaces of the Pd/$Ni(OH)_2$ catalyst enhanced the

reaction kinetics of HMFCA oxidation by increasing OH species and lowering the energy barrier (C-H bond activation) for HMFCA oxidation. Our results show that the oxidation of the aldehyde group of HMF will preferentially occur on the Pd^0 atoms of the $Pd/Ni(OH)_2$ catalyst, while the oxidation of the hydroxylmethyl group of HMFCA will preferentially occur at Ni^{2+}-O-Pd interfaces. Our results also suggest that the prepared $Pd/Ni(OH)_2$ catalyst has combined the advantages of Pd^0 sites and Ni^{2+}-O-Pd interfaces, both of which worked synergetically with a tandem mechanism to selectively produce FDCA from HMFOR. Thanks to the reviewer's comments that the potential active species from Ni and Pd in the electrolyte solution has been addressed and excluded in the *RESPONSE TO COMMENT 2* above. In addition, the Ni^{2+}-O-Pd interfaces effectively suppressed the competing decarbonylation reaction, thereby improving the carbon balance of HMFOR and increasing the Faradaic efficiency to FDCA.

Last, this work demonstrated the superior overall performance and stability of our $Pd/Ni(OH)_2$ catalyst under practically relevant operating conditions. We carried out the performance and stability test on our $Pd/Ni(OH)_2$ catalyst under a practically relevant electrochemical flow cell system (a two-electrode system) as a Demon, which can perform HMFOR to generate high purity FDCA product continuously. The scheme and image of the flow cell system are shown in Fig. 3f and Supplementary Fig. S32. The anodic reaction of HMFOR was coupled with the cathodic reaction of hydrogen evolution reaction (HER) during the flow cell operation. As shown in Fig. 3g, with our $Pd/Ni(OH)_2$ catalyst in the flow cell device, 100% HMF conversion and > 90% FDCA selectivity were achieved with the current density remaining unchanged during a 200-h continuous HMFOR operation under a fixed cell voltage of 0.85 V and a fixed electrolyte (5 mM HMF in 1 M KOH) flow rate of 1 mL/min. To further illustrate the practical performance, our $Pd/Ni(OH)_2$ catalyst can also work efficiently and stably for more than 24 hours without degradation under the current density of ~380 mA/cm^2 in the same flow cell device with similar operation parameters except for the increased cell voltage (1.05 V), concentration of HMF (125 mM HMF in 1 M KOH) and a flow rate (2.5 mL/min) of the electrolyte (Supplementary Fig. S35). In general, our $Pd/Ni(OH)_2$ catalyst with abundant Ni^{2+}-O-Pd interfaces can substantially reduce the cell-operation voltage and improve the Faradaic efficiency and selectivity toward the biomass-based FDCA. Therefore, this work has the potential to be developed for the industrial implementation of FDCA production.

Finally, based on the above summary, we hope the reviewer get a deeper understanding of the novelty and significance of our work. Thank you for your time and contribution to our work.

REVIEWERS' COMMENTS

Reviewer #2 (Remarks to the Author):

The authors have adequetly revised the manuscript and have addressed all concerns. I have no further critiques and support the publication of this work in Nature Communications.

Reviewer #3 (Remarks to the Author):

I have now read the rebuttal letter as well as the revised article, and based on the provided information and revisions I am not supportive of the revised article for publication in Nature Communications. Hence, I believe that the article provides a new angle to this important area; but also has now more depth in terms of what is exactly responsible for the activity as well as the origin and type of products made.
